# Industry Involvement and Transparency in the Most Cited Clinical Trials, 2019-2022

**DOI:** 10.1001/jamanetworkopen.2023.43425

**Published:** 2023-11-14

**Authors:** Leonardo M. Siena, Lazaros Papamanolis, Maximilian J. Siebert, Rosa Katia Bellomo, John P. A. Ioannidis

**Affiliations:** 1 Department of Public Health and Infectious Diseases, Sapienza University of Rome, Rome, Italy; 2 Meta-Research Innovation Center at Stanford, Stanford University, Stanford, California; 3 Department of Medicine, Stanford University, Stanford, California; 4 Department of Epidemiology and Population Health, Stanford University, Stanford, California; 5 Department of Biomedical Data Science, Stanford University, Stanford, California; 6 Department of Statistics, Stanford University, Stanford, California

## Abstract

**Importance:**

Industry involvement is prominent in influential clinical trials, and commitments to transparency of trials are highly variable.

**Objective:**

To evaluate the modes of industry involvement and the transparency features of the most cited recent clinical trials across medicine.

**Design, Setting, and Participants:**

This cross-sectional study was a meta-research assessment including randomized and nonrandomized clinical trials published in 2019 or later. The 600 trials of any type of disease or setting that attracted highest number of citations in Scopus as of December 2022 were selected for analysis. Data were analyzed from March to September 2023.

**Main Outcomes and Measures:**

Outcomes of interest were industry involvement (sponsor, author, and analyst) and transparency (protocols, statistical analysis plans, and data and code availability).

**Results:**

Among 600 trials with a median (IQR) sample size of 415 (124-1046) participants assessed, 409 (68.2%) had industry funding and 303 (50.5%) were exclusively industry-funded. A total of 354 trials (59.0%) had industry authors, with 280 trials (46.6%) involving industry analysts and 125 trials (20.8%) analyzed exclusively by industry analysts. Among industry-funded trials, 364 (89.0%) reached conclusions favoring the sponsor. Most trials (478 trials [79.7%]) provided a data availability statement, and most indicated intention to share the data, but only 16 trials (2.7%) had data already readily available to others. More than three-quarters of trials had full protocols (482 trials [82.0%]) or statistical analysis plans (446 trials [74.3%]) available, but only 27 trials (4.5%) explicitly mentioned sharing analysis code (8 readily available; 19 on request). Randomized trials were more likely than nonrandomized studies to involve only industry analysts (107 trials [22.9%] vs 18 trials [13.6%]; *P* = .02) and to have full protocols (405 studies [86.5%] vs 87 studies [65.9%]; *P* < .001) and statistical analysis plans (373 studies [79.7%] vs 73 studies [55.3%]; *P* < .001) available. Almost all nonrandomized industry-funded studies (90 of 92 studies [97.8%]) favored the sponsor. Among industry-funded trials, exclusive industry funding (odds ratio, 2.9; 95% CI, 1.5-5.4) and industry-affiliated authors (odds ratio, 2.9; 95% CI, 1.5-5.6) were associated with favorable conclusions for the sponsor.

**Conclusions and Relevance:**

This cross-sectional study illustrates how industry involvement in the most influential clinical trials was prominent not only for funding, but also authorship and provision of analysts and was associated with conclusions favoring the sponsor. While most influential trials reported that they planned to share data and make both protocols and statistical analysis plans available, raw data and code were rarely readily available.

## Key Points

**Question:**

What are the ways industry gets involved in the most influential clinical trials and how do these trials fare in terms of transparency?

**Findings:**

This cross-sectional study of 600 of the most-cited clinical trials published after 2018 found that industry involvement was very common for funding, authorship, and provision of analysts and conclusions in industry-sponsored studies typically favored the sponsor. While most trials shared protocols and statistical analysis plans and promised to share data, few had readily available data and code.

**Meaning:**

These findings suggest that industry involvement was very common and multifaceted in the most influential clinical research and both trial independence and transparency can be improved.

## Teaser

This cross-sectional study evaluates the modes of industry involvement and the transparency features of the most cited recent clinical trials across medicine.

## Introduction

Industry involvement has acquired over time a dominant presence in clinical research, particularly in clinical trials.^1^ Concurrently, the commitments made by companies to transparency of trials are highly variable,^2^ and not enough attention is paid to ensuring the public availability of a priori versions of trial protocols and statistical analysis plans.^3^ While influential trials published in major journals are practically ubiquitously registered in ClinicalTrials.gov or other trials registries, such registration alone may not provide detailed enough information to make the research sufficiently transparent and reproducible. Transparency would require availability of full trial protocols and statistical analysis plans, and eventually also the raw data collected and analyzed in the trial. While there are no systematic data available, it is likely that many, perhaps most, trials sponsored by the industry are only analyzed by industry-based statistical analysts and the raw data are not shared more widely, even with the participating trialists. There have been many initiatives to enhance data sharing from clinical trials in the last decade.^4,5^ Nonetheless, the implementation of data-sharing requirements, such as data statements in journal publication policies requested by the International Committee of Medical Journal Editors,^5^ is still suboptimal and does not guarantee wide, routine access to raw data.^6^ The COVID-19 pandemic also added new challenges and further emphasized the need for transparency and sharing.^7^

Given the major impact of influential clinical trials on medical practice, it is important to evaluate the extent of industry involvement and the use of transparency practices in such studies published in the last few years. Various features, such as industry analyst involvement, as well as protocol, statistical analysis plan, and raw data availability, are essential to understand in the evolving clinical trials transparency landscape. Therefore, we aimed to explore the characteristics of the most cited clinical trials published after 2018, investigating the involvement of the industry and the transparency features in these highly influential studies.

## Methods

The cross-sectional study is a review of existing literature; therefore, no institutional review board or ethics committee approval or informed consent was necessary as is standard protocol. This study follows the Strengthening the Reporting of Observational Studies in Epidemiology (STROBE) reporting guideline for observational studies. This study is a systematic assessment of the characteristics of the most cited clinical trials published after 2018, focusing on the involvement of the industry and the transparency features in these studies. The protocol of this study has been registered on the Open Science Framework^8^ before data extraction.

### Sample of Eligible Trials

The clinical trials that have been surveyed were those that had attracted the highest number of citations as of December 2022 in the Scopus database and had been published in 2019 or later. We considered as trials the research studies in which human participants were prospectively assigned to 1 or more interventions to evaluate the effects of those interventions on health-related outcomes. Only trials written in English were considered. Studies reporting information about more than 1 trial were excluded. Both randomized and nonrandomized trials were eligible.

### Search Strategy and Screening

In the first step, a researcher screened, assessing by title and abstract, the most cited articles published in 2019 or later in the Scopus database. Articles were ranked according to how many times they were cited in Scopus. From these, the 600 clinical trials with the highest number of citations were retrieved. A full list of included studies is provided in the eReferences in Supplement 1. Preliminary screening to identify the 30 most-cited trials published in 2019 (using all 2019 publications in the screening set and ordering them by decreasing citations) revealed that all of these 30 trials would be captured by using a search string randomized OR randomised OR trial*. This means that sensitivity of this search string was 100% (95% CI, 88.4%-100%). Therefore, we used this search string to increase the specificity of the search without substantial loss of sensitivity. The screening was conducted by L.M.S. Data extraction from the full text of these publications was performed.

### Data Extraction

From the full text versions of each of the 600 eligible articles, 2 independent investigators manually extracted data on the journal, type of study, location of the trial, industry affiliation for any author, for the first author, and for the corresponding author, funding, whether the analysis was conducted by industry analysts or not, presence of the data availability statement, declared data sharing, type of declared available data, access to data, conditional data access (for these latter 3 variables, we used the same categories as in a previous evaluation^9^), full protocol availability and statistical analysis plan availability, analysis code sharing, sample size, individual or cluster design, disease studied, intervention comparison, and whether the trial conclusions favored an industry-sponsored intervention (based on the conclusion of the abstract). Favorable conclusion included language supporting superiority for superiority trials or noninferiority for noninferiority trials, either for the entire study population or for a subgroup. It also includes favorable statements on safety for trials that make no comments on effectiveness. When multiple first coauthors or corresponding coauthors were present, affiliation to industry was attributed if at least 1 of them had an industry affiliation. Availability of full trial protocol and/or statistical analysis plan was probed also through ClinicalTrials.gov and PubMed. Protocols and statistical analysis plans were perused for extracting whether the analysis was conducted by industry analyses or not, when this was unclear from the published article. External links within the data sharing statement redirecting to data sharing policies were investigated when information provided in the data sharing statement was not sufficient. We used internet searches (Google; Alphabet) to clarify the nature of institutions and funding sources whenever these were unclear. Data extraction was conducted by L.M.S., L.P., M.J.S., and R.K.B. Any remaining discrepancies were settled by discussion among the investigators. A pilot of 10 full texts was conducted to validate the extraction table.

### Key Outcomes

We assessed 7 main outcomes. Main outcomes were the proportions of the most-cited clinical trials that were funded by industry (ie, for-profit companies), that had any author affiliated with the industry, with data analyzed by industry analysts, with raw data available, with a published or otherwise readily available (nonpaywalled) protocol, with a published or otherwise readily available (nonpaywalled) statistical analysis plan, and with a conclusion favoring an industry sponsor.

### Statistical Analysis

Descriptive statistics of trial characteristics and key outcomes were reported and described by counts, percentages, mean (or median) and SD (or range), and the corresponding 95% CIs, as appropriate. We also evaluated, using Pearson χ^2^ or Fisher exact test, whether each of the key outcomes differed according to whether the trials were on COVID-19 or other conditions, randomized or not, country (US vs other), sample size tertiles, and (for non–COVID-19 trials) disease focus (cancer, cardiovascular, other). Finally, we assessed whether each of the first 6 key outcomes was associated with the proportion of most-cited clinical trials with a conclusion favoring an industry sponsor for industry-sponsored trials. Statistical analysis was performed by L.M.S. A 2-sided *P*  < .005 was considered statistically significant, and *P* < .05 was considered suggestive.^10^ All analyses were performed using Stata software version 17.0 (StataCorp). Data were analyzed from March to September 2023.

## Results

### Characteristics of the Included Studies

The 600 most highly cited trials had a median (IQR) sample size of 415 (124-1046) participants. As shown in Table 1, 300 studies (50.0%) were published in 2019, 204 studies (34.0%) in 2020, 91 studies (15.2%) in 2021, and only 5 studies (0.8%) in 2022. While these studies were published across more than 70 different journals, major general medical journals published most of the studies. More than half of the clinical trials (343 studies [57.2%]) involved collaboration among multiple countries. Almost 80% of the studies (468 studies [78.0%]) were randomized, while a smaller proportion were nonrandomized controlled (7 studies [1.2%]) or single-group studies (125 studies [20.8%]). Two-thirds of the studies were superiority randomized trials (Table 1). Almost half the studies were on oncological conditions (260 studies [43.3%]), while infectious diseases (119 studies [19.8%], of which 107 studies were on COVID-19) and cardiology (60 studies [10.0%]) trials were also common.

**Table 1.** Characteristics of the Included Studies

Variable	Studies, No. (%) (N = 600)
Year	
2019	300 (50.0)
2020	204 (34.0)
2021	91 (15.2)
2022	5 (0.8)
Journal	
* The New England Journal of Medicine*	218 (36.3)
* The Lancet*	77 (12.8)
* The Lancet Oncology*	50 (8.3)
* Journal of Clinical Oncology*	41 (6.8)
* JAMA*	30 (5.0)
* JAMA Oncology*	19 (3.2)
* Nature Medicine*	15 (2.5)
* Annals of Oncology*	12 (2.0)
Other	139 (23.1)
Location^a^	
International	343 (57.2)
United States	86 (14.3)
China	36 (6.0)
United Kingdom	29 (4.8)
France	13 (2.2)
The Netherlands	12 (2.0)
Brazil	10 (1.7)
Other	71 (11.8)
Type of study	
Randomized	468 (78.0)
Nonrandomized single arm	125 (20.8)
Nonrandomized controlled	7 (1.2)
Design	
Individual	597 (99.5)
Cluster	3 (0.5)
Intervention comparison	
Superiority	400 (66.7)
Noninferiority	39 (6.5)
Equivalence	2 (0.3)
Not applicable	159 (26.5)
Disease in study	
Oncology	260 (43.3)
Infectious diseases^b^	119 (19.8)
Cardiology	60 (10.0)
Other	161 (26.9)

^a^ Considered all the countries where the trial was conducted.

^b^ Includes 107 studies on COVID-19.

### Funding and Industry Involvement

As shown in Table 2, more than half of the trials (303 trials [50.5%]) were funded exclusively by industry and 409 trials (68.2%) had any industry funding, but a substantial number of trials were funded only by public funds or combined sources not involving the industry. While 354 trials (59.0%) had at least 1 author with industry affiliation, few trials reported industry affiliation for the first (27 studies [4.5%]) or corresponding (44 studies [7.3%]) author. Almost half of the trials (280 studies [46.6%]) involved industry analysts, and 125 trials (20.8%) were analyzed exclusively by industry analysts. Among 409 studies funded by industry, either alone or in combination with other funding sources, most reached conclusions in favor of the intervention (364 studies [89.0%]).

**Table 2.** Funding, Industry Involvement, and Transparency Features of the Included Studies

Variable	Studies, No. (%) (N = 600)
Funding	
Combinations^a^	163 (27.2)
Industry	303 (50.5)
Public	104 (17.3)
Not reported	8 (1.3)
Academic	5 (0.8)
Other	17 (2.9)
Industry affiliation among authors	
Any	354 (59.0)
First author	27 (4.5)
Corresponding author	44 (7.3)
Analysts affiliation	
Only nonindustry	271 (45.2)
Both by industry and nonindustry	155 (25.8)
Only by industry analysts	125 (20.8)
Not specified	49 (8.2)
Results favored an industry-sponsored intervention	
Yes	364 (60.6)
No	45 (7.5)
No industry-sponsored intervention involved	190 (31.7)
Not applicable	1 (0.2)
Data availability statement	478 (79.7)
Declared data sharing (n = 478)	371 (77.6)
Type of declared available data (n = 371)	
Deidentified individual-participant data	245 (66.0)
Unspecified	100 (27.0)
Partial data	26 (7.0)
Aggregate data only	0
Access to data (n = 371)	
Request to authors	114 (30.7)
Request to repository or archive	104 (28.0)
Request to company	82 (22.1)
Request to committee, group, or unit	29 (7.8)
Access unspecified	26 (7.0)
Data available to others	16 (4.4)
Conditional data access (n = 154)^b^	
Data embargo ≤1 y	60 (39.0)
Data embargo >1 to 2 y	38 (24.7)
Data embargo >2 y	11 (7.1)
Product approval	45 (29.2)
Collaboration	0
Full protocol availability	
Published in peer-reviewed journal	410 (68.3)
None	108 (18.0)
Published online	82 (13.7)
Statistical analysis plan availability	
Published in peer-reviewed journal	354 (59.0)
None	154 (25.7)
Published online	92 (15.3)
Analysis code sharing	27 (4.5)

^a^ There were 106 studies with combination funding in which at least 1 of the funders was industry.

^b^ Studies where the data, regardless of access method, would not be shared before a specific time interval or event such as approval of the study intervention were considered as conditional data access.

### Transparency Features

As shown in Table 2, 478 trials (79.8%) provided a data availability statement, and three-quarters among these (371 trials [77.6%]) indicated their intention to share the data, usually (245 trials [66.0%]) as anonymized individual-participant data. The most commonly mentioned methods for accessing the data were requesting them from the authors (114 trials [30.5%]), from a repository or archive (104 trials [28.0%]), or from the respective company (82 trials [22.1%]). However, in 109 studies (18.2%), the data were subject to an embargo of various duration, and 45 studies (7.5%) stated that the data would only be shared after the approval of the product. Data were already available to others at the time of the data extraction in only 16 studies (2.7%).

A total of 492 trials (82.0%) had a full protocol available, with most published in peer-reviewed journals (Table 2). Furthermore, 446 studies (74.3%) had accessible statistical analysis plans, again, with most published in peer-reviewed journals, but only 27 studies (4.5%) explicitly mentioned that they would share the statistical analysis code (in 8 studies, the code was already available to others, while in 19 studies, the code had to be requested). Of 600 trials, only 3 had both the data and the code already available to others.

### Factors Associated With Industry Involvement and Transparency Features

Trials exclusively funded by industry more often involved industry-affiliated authors and analysts, almost always favored the sponsor, rarely allowed access to data, and shared protocols and analysis plans more frequently than other trials (Table 3). After adjusting for disease type (COVID-19 vs other), design (randomized vs nonrandomized), and location (exclusively US vs other), trials funded by industry remained unlikely to make the data (odds ratio [OR], 0.09; 95% CI, 0.01-0.7) or the code (OR, 0.3; 95% CI, 0.1-0.9) available, but more likely to make the protocol (OR, 2.0; 95% CI, 0.9-4.3) and the analysis plan (OR, 2.3; 95% CI, 1.2-4.5) available. COVID-19 trials exhibited a lower occurrence of exclusive funding from the industry, less common presence of an author affiliated with the industry, and a higher frequency of results not favoring industry-sponsored interventions (Table 3).

**Table 3.** Key Outcomes by Funding, Disease, Randomization, and Location

Outcome	Studies, No. (%)								
	Funding		Disease		Type of study		Location	
	Industry exclusively	Other sources or combinations	COVID-19	Other	Randomized	Nonrandomized	US	Other or international
Funding								
Industry exclusively	NA	NA	24 (22.4)^a^	279 (43.4)^a^	241 (51.5)	62 (47.0)	31 (36.1)^a^	272 (52.9)^a^
Other sources or combinations	NA	NA	83 (77.6)^a^	214 (56.6)^a^	227 (48.5)	70 (53.0)	55 (63.9)^a^	242 (47.1)^a^
Industry affiliation for any author								
Yes	279 (92.1)^a^	75 (25.2)^a^	51 (47.7)^b^	303 (61.5)^b^	269 (57.5)	85 (64.4)	36 (41.9)^a^	318 (61.9)^a^
No	24 (7.9)^a^	222 (74.8)^a^	56 (52.3)^b^	190 (38.5)^b^	199 (42.5)	47 (35.6)	50 (58.1)^a^	196 (38.1)^a^
Analysts’ affiliation								
Only by industry analysts	113 (37.3)^a^	12 (4.0)^a^	21 (19.6)	104 (21.1)	107 (22.9)^b^	18 (13.6)^b^	11 (12.8)^b^	114 (22.2)^b^
Other	190 (62.7)^a^	285 (96.0)^a^	86 (80.4)	389 (78.9)	361 (77.1)^b^	114 (86.4)^b^	75 (87.2)^b^	400 (77.8)^b^
Access to data								
Data are available to others	1 (0.3)^a^	15 (5.0)^a^	2 (1.9)	14 (2.8)	11 (2.4)	5 (3.8)	8 (9.3)^a^	8 (1.6)^a^
No access or other ways to access	302 (99.7)^a^	282 (95.0)^a^	105 (98.1)	479 (97.2)	457 (97.6)	127 (96.2)	78 (90.7)^a^	506 (98.4)^a^
**Full protocol availability**									
Yes	278 (91.8)^a^	214 (72.1)^a^	83 (77.6)	409 (83.0)	405 (86.5)^a^	87 (65.9)^a^	65 (75.6)	427 (83.1)
No	25 (8.2)^a^	83 (27.9)^a^	24 (22.4)	84 (17.0)	63 (13.5)^a^	45 (34.1)^a^	21 (24.4)	87 (16.9)
**Statistical analysis plan availability**									
Yes	262 (86.5)^a^	184 (62.0)^a^	69 (64.5)	377 (76.5)	373 (79.7)^a^	73 (55.3)^a^	56 (65.1)^b^	390 (75.9)^b^
No	41 (13.5)^a^	113 (38.0)^a^	38 (35.5)	116 (23.5)	95 (20.3)^a^	59 (44.7)^a^	30 (34.9)^b^	124 (24.1)^b^
**Results favoring sponsor (for industry-funded trials, n = 409)**									
Yes	279 (92.1)^a^	85 (80.2)^a^	38 (79.2)^b^	326 (90.3)^b^	274 (86.4)^a^	90 (97.8)^a^	44 (97.8)^b^	320 (87.9)^b^
No	24 (7.9)^a^	21 (19.8)^a^	10 (20.8)^b^	35 (9.7)^b^	43 (13.6)^a^	2 (2.2)^a^	1 (2.2)^b^	44 (12.1)^b^

Abbreviation: NA, not applicable.

^a^ *P* < .005.

^b^ *P* < .05.

Randomized trials, compared with nonrandomized studies, were more often analyzed exclusively by analysts affiliated with industry (107 trials [22.9%] vs 18 trials [13.6%]; *P* = .02) and demonstrated a greater availability of protocols (405 studies [86.5%] vs 87 studies [65.9%]; *P* < .001) and statistical analysis plans (373 studies [79.7%] vs 73 studies [55.3%]; *P* < .001). Conversely, nonrandomized studies exhibited more favorable results for industry-sponsored interventions than randomized trials (90 studies [97.8%] vs 274 studies [86.4%]; *P* = .002) (Table 3).

Trials exclusively conducted in the US exhibited a lower likelihood of being solely funded by industry, were less likely to have at least 1 author affiliated with industry, and were less frequently analyzed exclusively by industry analysts (Table 3). Of 16 studies that made their data accessible to others, half were exclusively conducted in the US (8 studies [9.3%] vs 8 studies [1.6%]; *P* < .001), and this group of trials shared the statistical analysis plan more frequently. Notably, almost all US-based clinical trials favored an industry-sponsored intervention (Table 3).

Trials belonging to the first tertile of sample size demonstrated a lower likelihood of being solely funded by industry (first tertile, 82 studies [41.0%]; second tertile, 117 studies [58.2%]; third tertile, 104 studies [52.3%]; *P* = .002) (Figure, A). Additionally, these trials less frequently had at least 1 author affiliated with the industry. Among 16 trials that made their data accessible to others, 11 fell into the smaller tertile of sample size. Conversely, availability of full protocols (first tertile, 136 studies [68.0%]; second tertile, 173 studies [86.1%]; third tertile, 183 studies [92.0%]; *P* < .001) and statistical analysis plans (first tertile, 114 studies [57.0%]; second tertile, 159 studies [79.1%]; third tertile, 173 studies [86.9%]; *P* < .001) increased as the number of study participants increased.

**Figure.**
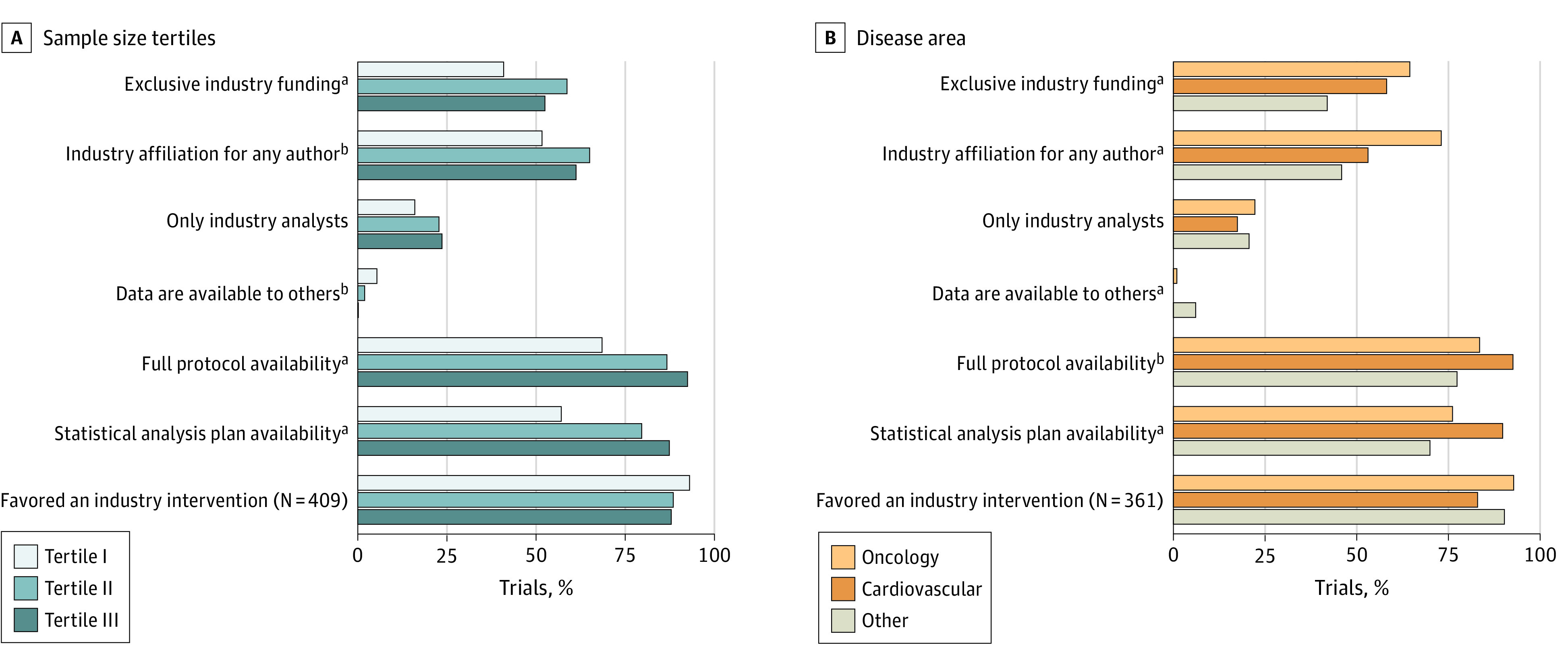
Key Outcomes by Sample Size Tertiles and by Disease Area Tertile 1 indicates the smallest sample sizes; tertile 3, the largest. ^a^*P* < .005. ^b^*P* < .05.

Among non–COVID-19 trials (Figure, B), oncology trials exhibited the highest frequency of exclusive industry sponsorship and involvement of at least 1 author affiliated with the industry. Trials focusing on cardiovascular disease were more frequently sharing the protocol and had a higher availability of the statistical analysis plan compared with trials in other disease areas. Trials in other disease areas were more likely to make their data accessible to others compared with oncology and cardiovascular studies (Table 3).

### Factors Associated With Results Favoring the Industry Intervention

Studies with favorable results for industry-sponsored interventions exhibited a higher prevalence, compared with others, of being exclusively sponsored by the industry (OR, 2.9; 95% CI, 1.5-5.4; *P* = .001). These studies were also more likely to have at least 1 author affiliated with the industry (OR, 2.9; 95% CI, 1.5-5.6; *P* = .001) (Table 4).

**Table 4.** Key Outcomes by Whether Conclusions Favored Industry Intervention in Industry-Sponsored Trials

Variable	Results favored sponsor No./total No. (%) (N = 409)	OR (95% CI)
Funding		
Industry exclusively	279/303 (92.1)^a^	2.9 (1.5-5.4)
Combinations including industry	85/106 (80.1)^a^	1 [Reference]
Industry affiliation for any author		
Yes	301/329 (91.5)^a^	2.9 (1.5-5.6)
No	63/80 (78.8)^a^	1 [Reference]
Analysts’ affiliation		
Only by industry analysts	114/123 (92.7)	1.8 (0.9-3.9)
Other	250/286 (87.4)	1 [Reference]
Access to data		
Data available to others	5/5 (100.0)	1.4 (0.1-25)
No access or other ways to access	359/404 (88.9)	1 [Reference]
Full protocol availability		
Yes	322/359 (89.7)	1.7 (0.7-3.8)
No	42/50 (84.0)	1 [Reference]
Statistical analysis plan availability		
Yes	300/336 (89.3)	1.2 (0.5-2.6)
No	64/73 (87.7)	1 [Reference]

Abbreviation: OR, odds ratio.

^a^ *P* < .005.

## Discussion

This cross-sectional study of the 600 most-cited clinical trials published in the last 4 years found that industry involvement in these extremely influential studies was prominent not only at the level of funding but extended also to very frequent inclusion of industry-affiliated authors and of industry-affiliated analysts. While industry-affiliated authors were rarely the first or corresponding author, it was common for industry analysts to be the only analysts involved in the trial. Industry involvement was associated with higher chances of the results favoring the sponsor. Most of these highly influential trials planned to share data, and they made both protocols and statistical analysis plans available. Protocols and statistical analysis codes were particularly commonly available for randomized trials. Nonrandomized studies had less transparency in this regard, and they almost always reached conclusions that favored the sponsor. Although most data statements promised eventually some data availability, immediate unobstructed access to both raw data and code was rare, hindering timely reproducibility checks and independent reuse of raw data for secondary analyses and meta-analyses.

The prominence of industry funding in influential trials is unsurprising.^11^ Industry mobilizes resources, expertise, and infrastructure for clinical trials, while public funding is limited.^12^ Industry trials may thus have competitive advantages in quality and timeliness.^13,14^ Nevertheless, what is less recognized is the high penetration of industry authors and analysts in industry-funded trials. Previous work had suggested that there was a high rate of ghost authorship in clinical trials.^15^ Compared with this misleading practice that hides and diverts accountability, it is preferable to have industry scientists appear as regular authors, although it is unknown whether additional ghost authors may exist. The frequent involvement of industry-based analysts (often without any nonindustry analyst being involved) is also of some concern. It may be best to ensure analytical independence for these influential studies. Nonindustry investigators and institutions should strive to control analyses. Analytical independence would diminish perceptions of bias. This change would require strengthening data science and biostatistics resources in academic and public institutions. The high rates of sponsor-favorable conclusions align with previous evidence showing industry-sponsored research reaches favorable conclusions more often than non–industry-funded studies.^16^ The difference is more at the level of study design (eg, industry-funded research being more likely to choose placebo or strawman comparators for industry-sponsored interventions) and of interpretation (favorable spin).^17,18^ Our results do not necessarily prove industry bias. However, they suggest that enhanced analytical and overall independence in the conduct and reporting of these influential studies and maximal transparency are needed to alleviate concerns for bias. This applies not only to randomized trials, but even more so to nonrandomized studies. In our sample, almost all nonrandomized studies funded by industry ended up favoring the sponsor. Nonrandomized studies are becoming increasingly common in major licensing decisions.^19^ They also tend to be less transparent than randomized trials, which makes the possibility of bias even more likely.

The high rates of promised data sharing and of readily available full protocols and statistical analysis plans observed in this cohort of studies are a welcome observation. Data sharing statements have become highly popular and are routinely used, especially by journals that are likely to publish influential clinical research.^6^ Moreover, empirical studies have shown the increasing use of full protocols and of statistical analysis plans.^20^ Among publications in the most influential medical journals, mean availability of these items has surpassed 80% for protocols and 50% for analysis plans.^3^ However, it is uncommon to find a priori registered versions of these documents,^3^ and unexplained discrepancies in the statistical methods are common.^21^ It was disappointing that raw data and code were rarely available immediately and without additional obstacles, eg, embargo periods or required approvals. Previous work has shown similar patterns on data sharing for trials published by influential general medical journals in 2018 to 2020.^9^ Sharing data and codes that work outside their original environment is essential to maximizing trust for the most influential studies, since these studies largely shape the rest of the medical and scientific literature.^22^

### Limitations

This study has some limitations. The focus on recent trials published after 2018 meant that a substantial number of trials in the sample were on COVID-19, given the immense attention given to the pandemic in 2020 to 2021.^23^ However, this allowed us to examine the features of these trials compared with other medical fields and specialties, which have traditionally been characterized by different levels of transparency and industry involvement.

Some studies used ambiguous wording regarding the exact individual contributions of the authors, which made it difficult to discern who were the analysts of the trial, and in 49 studies, information on the analysts was not reported. Regarding data sharing, in some trials where multiple types of data were shared with varying access conditions, strict classification based on the predefined data extraction protocol was challenging.

Protocol and statistical data analysis availability does not guarantee that these documents are complete and thorough, let alone that the presented results faithfully follow them.^24,25^ However, having such documents readily available does allow interested readers and reviewers to appraise the fidelity of the publications, something that goes beyond the scope of our work.

## Conclusions

Our cross-sectional study maps the extent and forms of industry involvement and the state of transparency indicators in the most influential recent clinical trials across medicine. Several areas of improvement are identified that should be discussed among diverse stakeholders, including industry, trialists, other funders, and institutions.

## Notes

Supplement 1.
eReferences

Supplement 2.
Data Sharing Statement
