# Supplementary material for: Industry Involvement and Transparency in the Most Cited Clinical Trials, 2019-2022
Source: JAMA Netw Open. 2023 Nov 14;6(11):e2343425. doi: 10.1001/jamanetworkopen.2023.43425 (PMC10646728; doi:10.1001/jamanetworkopen.2023.43425)
Supplement: Supplement 1. — eReferences [file jamanetwopen-e2343425-s001.pdf]

Supplemental Online Content

Siena LM, Papamanolis L, Siebert MJ, Bellomo RK, Ioannidis JPA. Industry involvement and transparency in the most cited clinical trials, 2019-2022. *JAMA Netw Open*. 2023;6(11):e2343425. doi:10.1001/jamanetworkopen.2023.43425

eReferences

This supplemental material has been provided by the authors to give readers additional information about their work.

© 2023 Siena LM et al. *JAMA Network Open.*

eReferences

1. Mehra MR, Uriel N, Naka Y, et al. A Fully Magnetically Levitated Left Ventricular Assist Device — Final Report. N Engl J Med. 2019;380(17):1618-1627. doi:10.1056/nejmoa1900486

2. Claassens DMF, Vos GJA, Bergmeijer TO, et al. A Genotype-Guided Strategy for Oral P2Y 12 Inhibitors in Primary PCI . N Engl J Med. 2019;381(17):1621-1631. doi:10.1056/nejmoa1907096

3. Newsome PN, Buchholtz K, Cusi K, et al. A Placebo-Controlled Trial of Subcutaneous Semaglutide in Nonalcoholic Steatohepatitis. N Engl J Med. 2021;384(12):1113-1124. doi:10.1056/nejmoa2028395

4. Loibl S, Untch M, Burchardi N, et al. A randomised phase II study investigating durvalumab in addition to an anthracycline taxane-based neoadjuvant therapy in early triple-negative breast cancer: Clinical results and biomarker analysis of GeparNuevo study. Ann Oncol. 2019;30(8):1279-1288. doi:10.1093/annonc/mdz158

5. Simonovich VA, Burgos Pratx LD, Scibona P, et al. A Randomized Trial of Convalescent Plasma in Covid-19 Severe Pneumonia. N Engl J Med. 2021;384(7):619-629. doi:10.1056/nejmoa2031304

6. Hajek P, Phillips-Waller A, Przulj D, et al. A Randomized Trial of E-Cigarettes versus Nicotine-Replacement Therapy. N Engl J Med. 2019;380(7):629-637. doi:10.1056/nejmoa1808779

7. Boulware DR, Pullen MF, Bangdiwala AS, et al. A Randomized Trial of Hydroxychloroquine as Postexposure Prophylaxis for Covid-19. N Engl J Med. 2020;383(6):517-525. doi:10.1056/nejmoa2016638

8. Mulangu S, Dodd LE, Davey RT, et al. A Randomized, Controlled Trial of Ebola Virus Disease Therapeutics. N Engl J Med. 2019;381(24):2293-2303. doi:10.1056/nejmoa1910993

9. Cao B, Wang Y, Wen D, et al. A Trial of Lopinavir–Ritonavir in Adults Hospitalized with Severe Covid-19. N Engl J Med. 2020;382(19):1787-1799. doi:10.1056/nejmoa2001282

10. Hilf N, Kuttruff-Coqui S, Frenzel K, et al. Actively personalized vaccination trial for newly diagnosed glioblastoma. Nature. 2019;565(7738):240-245. doi:10.1038/s41586-018-0810-y

11. Kelly RJ, Ajani JA, Kuzdzal J, et al. Adjuvant Nivolumab in Resected Esophageal or Gastroesophageal Junction Cancer. N Engl J Med. 2021;384(13):1191-1203. doi:10.1056/nejmoa2032125

12. Tutt ANJ, Garber JE, Kaufman B, et al. Adjuvant Olaparib for Patients with BRCA1 - or BRCA2 -Mutated Breast Cancer . N Engl J Med. 2021;384(25):2394-2405. doi:10.1056/nejmoa2105215

13. André F, Ciruelos E, Rubovszky G, et al. Alpelisib for PIK3CA -Mutated, Hormone Receptor–Positive Advanced Breast Cancer . N Engl J Med. 2019;380(20):1929-1940. doi:10.1056/nejmoa1813904

14. Herold KC, Bundy BN, Long SA, et al. An Anti-CD3 Antibody, Teplizumab, in Relatives at Risk for Type 1 Diabetes. N Engl J Med. 2019;381(7):603-613. doi:10.1056/nejmoa1902226

15. Jackson LA, Anderson EJ, Rouphael NG, et al. An mRNA Vaccine against SARS-CoV-2 — Preliminary Report. N Engl J Med. 2020;383(20):1920-1931. doi:10.1056/nejmoa2022483

16. Velazquez EJ, Morrow DA, DeVore AD, et al. Angiotensin–Neprilysin Inhibition in Acute Decompensated Heart Failure. N Engl J Med. 2019;380(6):539-548. doi:10.1056/nejmoa1812851

17. Solomon SD, McMurray JJV, Anand IS, et al. Angiotensin–Neprilysin Inhibition in Heart Failure with Preserved Ejection Fraction. N Engl J Med. 2019;381(17):1609-1620. doi:10.1056/nejmoa1908655

© 2023 Siena LM et al. *JAMA Network Open.*

18. Raje N, Berdeja J, Lin Y, et al. Anti-BCMA CAR T-Cell Therapy bb2121 in Relapsed or Refractory Multiple Myeloma. N Engl J Med. 2019;380(18):1726-1737. doi:10.1056/nejmoa1817226

19. Lopes RD, Heizer G, Aronson R, et al. Antithrombotic Therapy after Acute Coronary Syndrome or PCI in Atrial Fibrillation. N Engl J Med. 2019;380(16):1509-1524. doi:10.1056/nejmoa1817083

20. Matulonis UA, Shapira-Frommer R, Santin AD, et al. Antitumor activity and safety of pembrolizumab in patients with advanced recurrent ovarian cancer: results from the phase II KEYNOTE-100 study. Ann Oncol. 2019;30(7):1080-1087. doi:10.1093/annonc/mdz135

21. Chi KN, Agarwal N, Bjartell A, et al. Re: Apalutamide for metastatic, castration-sensitive prostate cancer. J Urol. 2019;202(4):661. doi:10.1056/NEJMoa1903307

22. Agnelli G, Becattini C, Meyer G, et al. Apixaban for the Treatment of Venous Thromboembolism Associated with Cancer. N Engl J Med. 2020;382(17):1599-1607. doi:10.1056/nejmoa1915103

23. Carrier M, Abou-Nassar K, Mallick R, et al. Apixaban to Prevent Venous Thromboembolism in Patients with Cancer. N Engl J Med. 2019;380(8):711-719. doi:10.1056/nejmoa1814468

24. Armstrong AJ, Szmulewitz RZ, Petrylak DP, et al. Arches: A randomized, phase III study of androgen deprivation therapy with enzalutamide or placebo in men with metastatic hormone-sensitive prostate cancer. J Clin Oncol. 2019;37(32):2974-2986. doi:10.1200/JCO.19.00799

25. Fendler WP, Calais J, Eiber M, et al. Assessment of 68Ga-PSMA-11 PET Accuracy in Localizing Recurrent Prostate Cancer: A Prospective Single-Arm Clinical Trial. JAMA Oncol. 2019;5(6):856-863. doi:10.1001/jamaoncol.2019.0096

26. Marabelle A, Fakih M, Lopez J, et al. Association of tumour mutational burden with outcomes in patients with advanced solid tumours treated with pembrolizumab: prospective biomarker analysis of the multicohort, open-label, phase 2 KEYNOTE-158 study. Lancet Oncol. 2020;21(10):1353-1365. doi:10.1016/S1470-2045(20)30445-9

27. Herbst RS, Giaccone G, de Marinis F, et al. Atezolizumab for First-Line Treatment of PD-L1–Selected Patients with NSCLC. N Engl J Med. 2020;383(14):1328-1339. doi:10.1056/nejmoa1917346

28. West H, McCleod M, Hussein M, et al. Atezolizumab in combination with carboplatin plus nab-paclitaxel chemotherapy compared with chemotherapy alone as first-line treatment for metastatic non-squamous non-small-cell lung cancer (IMpower130): a multicentre, randomised, open-label, phase 3 trial. Lancet Oncol. 2019;20(7):924-937. doi:10.1016/S1470-2045(19)30167-6

29. Reck M, Mok TS, Nishio M, et al. Atezolizumab plus bevacizumab and chemotherapy in non-small-cell lung cancer (IMpower150): key subgroup analyses of patients with EGFR mutations or baseline liver metastases in a randomised, open-label phase 3 trial. Lancet Respir Med. 2019;7(5):387-401. doi:10.1016/S2213-2600(19)30084-0

30. Finn RS, Qin S, Ikeda M, et al. Atezolizumab plus Bevacizumab in Unresectable Hepatocellular Carcinoma. N Engl J Med. 2020;382(20):1894-1905. doi:10.1056/nejmoa1915745

31. Rini BI, Powles T, Atkins MB, et al. Atezolizumab plus bevacizumab versus sunitinib in patients with previously untreated metastatic renal cell carcinoma (IMmotion151): a multicentre, open-label, phase 3, randomised controlled trial. Lancet. 2019;393(10189):2404-2415. doi:10.1016/S0140-6736(19)30723-8

32. Schmid P, Rugo HS, Adams S, et al. Atezolizumab plus nab-paclitaxel as first-line treatment for unresectable, locally advanced or metastatic triple-negative breast cancer (IMpassion130): updated efficacy

© 2023 Siena LM et al. *JAMA Network Open.*

results from a randomised, double-blind, placebo-controlled, phase 3 trial. Lancet Oncol. 2020;21(1):44-59. doi:10.1016/S1470-2045(19)30689-8

33. Powles T, Park SH, Voog E, et al. Avelumab Maintenance Therapy for Advanced or Metastatic Urothelial Carcinoma. N Engl J Med. 2020;383(13):1218-1230. doi:10.1056/nejmoa2002788

34. Motzer RJ, Penkov K, Haanen J, et al. Avelumab plus Axitinib versus Sunitinib for Advanced Renal-Cell Carcinoma. N Engl J Med. 2019;380(12):1103-1115. doi:10.1056/nejmoa1816047

35. DiNardo CD, Jonas BA, Pullarkat V, et al. Azacitidine and Venetoclax in Previously Untreated Acute Myeloid Leukemia. N Engl J Med. 2020;383(7):617-629. doi:10.1056/nejmoa2012971

36. Cohen AD, Garfall AL, Stadtmauer EA, et al. B cell maturation antigen–specific CAR T cells are clinically active in multiple myeloma. J Clin Invest. 2019;129(6):2210-2221. doi:10.1172/JCI126397

37. Kalil AC, Patterson TF, Mehta AK, et al. Baricitinib plus Remdesivir for Hospitalized Adults with Covid-19. N Engl J Med. 2021;384(9):795-807. doi:10.1056/nejmoa2031994

38. Lonial S, Lee HC, Badros A, et al. Belantamab mafodotin for relapsed or refractory multiple myeloma (DREAMM-2): a two-arm, randomised, open-label, phase 2 study. Lancet Oncol. 2020;21(2):207-221. doi:10.1016/S1470-2045(19)30788-0

39. Moreau P, Attal M, Hulin C, et al. Bortezomib, thalidomide, and dexamethasone with or without daratumumab before and after autologous stem-cell transplantation for newly diagnosed multiple myeloma (CASSIOPEIA): a randomised, open-label, phase 3 study. Lancet. 2019;394(10192):29-38. doi:10.1016/S0140-6736(19)31240-1

40. Horwitz S, O'Connor OA, Pro B, et al. Brentuximab vedotin with chemotherapy for CD30-positive peripheral T-cell lymphoma (ECHELON-2): a global, double-blind, randomised, phase 3 trial. Lancet. 2019;393(10168):229-240. doi:10.1016/S0140-6736(18)32984-2

41. de Wit R, de Bono J, Sternberg CN, et al. Cabazitaxel versus Abiraterone or Enzalutamide in Metastatic Prostate Cancer. N Engl J Med. 2019;381(26):2506-2518. doi:10.1056/nejmoa1911206

42. Perkovic V, Jardine MJ, Neal B, et al. Canagliflozin and Renal Outcomes in Type 2 Diabetes and Nephropathy. N Engl J Med. 2019;380(24):2295-2306. doi:10.1056/nejmoa1811744

43. Primrose JN, Neoptolemos J, Palmer DH, et al. Capecitabine compared with observation in resected biliary tract cancer (BILCAP): a randomised, controlled, multicentre, phase 3 study. Lancet Oncol. 2019;20(5):663-673. doi:10.1016/S1470-2045(18)30915-X

44. Scully M, Cataland SR, Peyvandi F, et al. Caplacizumab Treatment for Acquired Thrombotic Thrombocytopenic Purpura. N Engl J Med. 2019;380(4):335-346. doi:10.1056/nejmoa1806311

45. Wolf J, Seto T, Han JY, et al. Capmatinib in MET Exon 14–Mutated or MET -Amplified Non–Small-Cell Lung Cancer . N Engl J Med. 2020;383(10):944-957. doi:10.1056/nejmoa2002787

46. Packer M, Anker SD, Butler J, et al. Cardiovascular and Renal Outcomes with Empagliflozin in Heart Failure. N Engl J Med. 2020;383(15):1413-1424. doi:10.1056/nejmoa2022190

47. Cannon CP, Pratley R, Dagogo-Jack S, et al. Cardiovascular Outcomes with Ertugliflozin in Type 2 Diabetes. N Engl J Med. 2020;383(15):1425-1435. doi:10.1056/nejmoa2004967

48. Bhatt DL, Steg PG, Miller M, et al. Cardiovascular Risk Reduction with Icosapent Ethyl for Hypertriglyceridemia. N Engl J Med. 2019;380(1):11-22. doi:10.1056/nejmoa1812792

© 2023 Siena LM et al. *JAMA Network Open.*

49. Wilson MR, Sample HA, Zorn KC, et al. Clinical Metagenomic Sequencing for Diagnosis of Meningitis and Encephalitis. N Engl J Med. 2019;380(24):2327-2340. doi:10.1056/nejmoa1803396

50. Nidorf SM, Fiolet ATL, Mosterd A, et al. Colchicine in Patients with Chronic Coronary Disease. N Engl J Med. 2020;383(19):1838-1847. doi:10.1056/nejmoa2021372

51. Grein J, Ohmagari N, Shin D, et al. Compassionate Use of Remdesivir for Patients with Severe Covid-19. N Engl J Med. 2020;382(24):2327-2336. doi:10.1056/nejmoa2007016

52. Mehta SR, Wood DA, Storey RF, et al. Complete Revascularization with Multivessel PCI for Myocardial Infarction. N Engl J Med. 2019;381(15):1411-1421. doi:10.1056/nejmoa1907775

53. Agarwal A, Mukherjee A, Kumar G, Chatterjee P, Bhatnagar T, Malhotra P. Convalescent plasma in the management of moderate covid-19 in adults in India: Open label phase II multicentre randomised controlled trial (PLACID Trial). BMJ. 2020;371. doi:10.1136/bmj.m3939

54. Sahin U, Muik A, Derhovanessian E, et al. COVID-19 vaccine BNT162b1 elicits human antibody and TH1 T cell responses. Nature. 2020;586(7830):594-599. doi:10.1038/s41586-020-2814-7

55. Gillmore JD, Gane E, Taubel J, et al. CRISPR-Cas9 In Vivo Gene Editing for Transthyretin Amyloidosis. N Engl J Med. 2021;385(6):493-502. doi:10.1056/nejmoa2107454

56. Diener HC, Sacco RL, Easton JD, et al. Dabigatran for Prevention of Stroke after Embolic Stroke of Undetermined Source. N Engl J Med. 2019;380(20):1906-1917. doi:10.1056/nejmoa1813959

57. Wiviott SD, Raz I, Bonaca MP, et al. Dapagliflozin and Cardiovascular Outcomes in Type 2 Diabetes. N Engl J Med. 2019;380(4):347-357. doi:10.1056/nejmoa1812389

58. Heerspink HJL, Stefánsson B V., Correa-Rotter R, et al. Dapagliflozin in Patients with Chronic Kidney Disease. N Engl J Med. 2020;383(15):1436-1446. doi:10.1056/nejmoa2024816

59. McMurray JJV, Solomon SD, Inzucchi SE, et al. Dapagliflozin in Patients with Heart Failure and Reduced Ejection Fraction. N Engl J Med. 2019;381(21):1995-2008. doi:10.1056/NEJMoa1911303

60. Facon T, Kumar S, Plesner T, et al. Daratumumab plus Lenalidomide and Dexamethasone for Untreated Myeloma. N Engl J Med. 2019;380(22):2104-2115. doi:10.1056/nejmoa1817249

61. Fizazi K, Shore N, Tammela TL, et al. Re: Darolutamide in nonmetastatic, castration-resistant prostate cancer. J Urol. 2019;202(4):660-661. doi:10.1056/NEJMoa1815671

62. Dexamethasone in Hospitalized Patients with Covid-19. N Engl J Med. 2021;384(8):693-704. doi:10.1056/nejmoa2021436

63. Villar J, Ferrando C, Martínez D, et al. Dexamethasone treatment for the acute respiratory distress syndrome: a multicentre, randomised controlled trial. Lancet Respir Med. 2020;8(3):267-276. doi:10.1016/S2213-2600(19)30417-5

64. Gerstein HC, Colhoun HM, Dagenais GR, et al. Dulaglutide and cardiovascular outcomes in type 2 diabetes (REWIND): a double-blind, randomised placebo-controlled trial. Lancet. 2019;394(10193):121-130. doi:10.1016/S0140-6736(19)31149-3

65. Paz-Ares L, Dvorkin M, Chen Y, et al. Durvalumab plus platinum–etoposide versus platinum–etoposide in first-line treatment of extensive-stage small-cell lung cancer (CASPIAN): a randomised, controlled, open-label, phase 3 trial. Lancet. 2019;394(10212):1929-1939. doi:10.1016/S0140-6736(19)32222-6

© 2023 Siena LM et al. *JAMA Network Open.*

66. Libster R, Pérez Marc G, Wappner D, et al. Early High-Titer Plasma Therapy to Prevent Severe Covid-19 in Older Adults. N Engl J Med. 2021;384(7):610-618. doi:10.1056/nejmoa2033700

67. Early Neuromuscular Blockade in the Acute Respiratory Distress Syndrome. N Engl J Med. 2019;380(21):1997-2008. doi:10.1056/nejmoa1901686

68. Kirchhof P, Camm AJ, Goette A, et al. Early Rhythm-Control Therapy in Patients with Atrial Fibrillation. N Engl J Med. 2020;383(14):1305-1316. doi:10.1056/nejmoa2019422

69. Gupta A, Gonzalez-Rojas Y, Juarez E, et al. Early Treatment for Covid-19 with SARS-CoV-2 Neutralizing Antibody Sotrovimab. N Engl J Med. 2021;385(21):1941-1950. doi:10.1056/nejmoa2107934

70. Pittock SJ, Berthele A, Fujihara K, et al. Eculizumab in Aquaporin-4–Positive Neuromyelitis Optica Spectrum Disorder. N Engl J Med. 2019;381(7):614-625. doi:10.1056/nejmoa1900866

71. Vranckx P, Valgimigli M, Eckardt L, et al. Edoxaban-based versus vitamin K antagonist-based antithrombotic regimen after successful coronary stenting in patients with atrial fibrillation (ENTRUST-AF PCI): a randomised, open-label, phase 3b trial. Lancet. 2019;394(10206):1335-1343. doi:10.1016/S0140-6736(19)31872-0

72. Watanabe H, Domei T, Morimoto T, et al. Effect of 1-Month Dual Antiplatelet Therapy Followed by Clopidogrel vs 12-Month Dual Antiplatelet Therapy on Cardiovascular and Bleeding Events in Patients Receiving PCI: The STOPDAPT-2 Randomized Clinical Trial. JAMA - J Am Med Assoc. 2019;321(24):2414-2427. doi:10.1001/jama.2019.8145

73. Al Kaabi N, Zhang Y, Xia S, et al. Effect of 2 Inactivated SARS-CoV-2 Vaccines on Symptomatic COVID-19 Infection in Adults: A Randomized Clinical Trial. JAMA - J Am Med Assoc. 2021;326(1):35-45. doi:10.1001/jama.2021.8565

74. Hernández G, Ospina-Tascón GA, Damiani LP, et al. Effect of a Resuscitation Strategy Targeting Peripheral Perfusion Status vs Serum Lactate Levels on 28-Day Mortality among Patients with Septic Shock: The ANDROMEDA-SHOCK Randomized Clinical Trial. JAMA - J Am Med Assoc. 2019;321(7):654-664. doi:10.1001/jama.2019.0071

75. Xia S, Duan K, Zhang Y, et al. Effect of an Inactivated Vaccine Against SARS-CoV-2 on Safety and Immunogenicity Outcomes: Interim Analysis of 2 Randomized Clinical Trials. JAMA - J Am Med Assoc. 2020;324(10):951-960. doi:10.1001/jama.2020.15543

76. Gottlieb RL, Nirula A, Chen P, et al. Effect of bamlanivimab as monotherapy or in combination with etesevimab on viral load in patients with mild to moderate COVID-19: A randomized clinical trial. JAMA - J Am Med Assoc. 2021;325(7):632-644. doi:10.1001/jama.2021.0202

77. Entrenas Castillo M, Entrenas Costa LM, Vaquero Barrios JM, et al. "Effect of calcifediol treatment and best available therapy versus best available therapy on intensive care unit admission and mortality among patients hospitalized for COVID-19: A pilot randomized clinical study." J Steroid Biochem Mol Biol. 2020;203. doi:10.1016/j.jsbmb.2020.105751

78. Packer DL, Mark DB, Robb RA, et al. Effect of Catheter Ablation vs Antiarrhythmic Drug Therapy on Mortality, Stroke, Bleeding, and Cardiac Arrest among Patients with Atrial Fibrillation: The CABANA Randomized Clinical Trial. JAMA - J Am Med Assoc. 2019;321(13):1261-1274. doi:10.1001/jama.2019.0693

79. Li L, Zhang W, Hu Y, et al. Effect of Convalescent Plasma Therapy on Time to Clinical Improvement in Patients with Severe and Life-threatening COVID-19: A Randomized Clinical Trial. JAMA - J Am Med Assoc. 2020;324(5):460-470. doi:10.1001/jama.2020.10044

© 2023 Siena LM et al. *JAMA Network Open.*

80. Kato ET, Silverman MG, Mosenzon O, et al. Effect of Dapagliflozin on Heart Failure and Mortality in Type 2 Diabetes Mellitus. Circulation. 2019;139(22):2528-2536. doi:10.1161/CIRCULATIONAHA.119.040130

81. Tomazini BM, Maia IS, Cavalcanti AB, et al. Effect of Dexamethasone on Days Alive and Ventilator-Free in Patients with Moderate or Severe Acute Respiratory Distress Syndrome and COVID-19: The CoDEX Randomized Clinical Trial. JAMA - J Am Med Assoc. 2020;324(13):1307-1316. doi:10.1001/jama.2020.17021

82. Costello SP, Hughes PA, Waters O, et al. Effect of Fecal Microbiota Transplantation on 8-Week Remission in Patients with Ulcerative Colitis: A Randomized Clinical Trial. JAMA - J Am Med Assoc. 2019;321(2):156-164. doi:10.1001/jama.2018.20046

83. Bakris GL, Agarwal R, Anker SD, et al. Effect of Finerenone on Chronic Kidney Disease Outcomes in Type 2 Diabetes. N Engl J Med. 2020;383(23):2219-2229. doi:10.1056/nejmoa2025845

84. Borba MGS, Val FFA, Sampaio VS, et al. Effect of High vs Low Doses of Chloroquine Diphosphate as Adjunctive Therapy for Patients Hospitalized With Severe Acute Respiratory Syndrome Coronavirus 2 (SARS-CoV-2) Infection: A Randomized Clinical Trial. JAMA Netw open. 2020;3(4):e208857. doi:10.1001/jamanetworkopen.2020.8857

85. Angus DC, Derde L, Al-Beidh F, et al. Effect of Hydrocortisone on Mortality and Organ Support in Patients with Severe COVID-19: The REMAP-CAP COVID-19 Corticosteroid Domain Randomized Clinical Trial. JAMA - J Am Med Assoc. 2020;324(13):1317-1329. doi:10.1001/jama.2020.17022

86. Effect of Hydroxychloroquine in Hospitalized Patients with Covid-19. N Engl J Med. 2020;383(21):2030-2040. doi:10.1056/nejmoa2022926

87. Williamson JD, Pajewski NM, Auchus AP, et al. Effect of Intensive vs Standard Blood Pressure Control on Probable Dementia: A Randomized Clinical Trial. JAMA - J Am Med Assoc. 2019;321(6):553-561. doi:10.1001/jama.2018.21442

88. Sadeghipour P, Talasaz AH, Rashidi F, et al. Effect of Intermediate-Dose vs Standard-Dose Prophylactic Anticoagulation on Thrombotic Events, Extracorporeal Membrane Oxygenation Treatment, or Mortality among Patients with COVID-19 Admitted to the Intensive Care Unit: The INSPIRATION Randomized Clinical Trial. JAMA - J Am Med Assoc. 2021;325(16):1620-1630. doi:10.1001/jama.2021.4152

89. Yu J, Huang C, Sun Y, et al. Effect of Laparoscopic vs Open Distal Gastrectomy on 3-Year Disease-Free Survival in Patients with Locally Advanced Gastric Cancer: The CLASS-01 Randomized Clinical Trial. JAMA - J Am Med Assoc. 2019;321(20):1983-1992. doi:10.1001/jama.2019.5359

90. Rosenstock J, Perkovic V, Johansen OE, et al. Effect of Linagliptin vs Placebo on Major Cardiovascular Events in Adults with Type 2 Diabetes and High Cardiovascular and Renal Risk: The CARMELINA Randomized Clinical Trial. JAMA - J Am Med Assoc. 2019;321(1):69-79. doi:10.1001/jama.2018.18269

91. Reardon DA, Brandes AA, Omuro A, et al. Effect of Nivolumab vs Bevacizumab in Patients with Recurrent Glioblastoma: The CheckMate 143 Phase 3 Randomized Clinical Trial. JAMA Oncol. 2020;6(7):1003-1010. doi:10.1001/jamaoncol.2020.1024

92. Theelen WSME, Peulen HMU, Lalezari F, et al. Effect of Pembrolizumab after Stereotactic Body Radiotherapy vs Pembrolizumab Alone on Tumor Response in Patients with Advanced Non-Small Cell Lung Cancer: Results of the PEMBRO-RT Phase 2 Randomized Clinical Trial. JAMA Oncol. 2019;5(9):1276-1282. doi:10.1001/jamaoncol.2019.1478

93. Spinner CD, Gottlieb RL, Criner GJ, et al. Effect of Remdesivir vs Standard Care on Clinical Status at 11 Days in Patients with Moderate COVID-19: A Randomized Clinical Trial. JAMA - J Am Med Assoc. 2020;324(11):1048-1057. doi:10.1001/jama.2020.16349

© 2023 Siena LM et al. *JAMA Network Open.*

94. Salvarani C, Dolci G, Massari M, et al. Effect of Tocilizumab vs Standard Care on Clinical Worsening in Patients Hospitalized with COVID-19 Pneumonia: A Randomized Clinical Trial. JAMA Intern Med. 2021;181(1):24-31. doi:10.1001/jamainternmed.2020.6615

95. Hermine O, Mariette X, Tharaux PL, Resche-Rigon M, Porcher R, Ravaud P. Effect of Tocilizumab vs Usual Care in Adults Hospitalized with COVID-19 and Moderate or Severe Pneumonia: A Randomized Clinical Trial. JAMA Intern Med. 2021;181(1):32-40. doi:10.1001/jamainternmed.2020.6820

96. Fowler AA, Truwit JD, Hite RD, et al. Effect of Vitamin C Infusion on Organ Failure and Biomarkers of Inflammation and Vascular Injury in Patients with Sepsis and Severe Acute Respiratory Failure: The CITRIS-ALI Randomized Clinical Trial. JAMA - J Am Med Assoc. 2019;322(13):1261-1270. doi:10.1001/jama.2019.11825

97. Xu X, Han M, Li T, et al. Effective treatment of severe COVID-19 patients with tocilizumab. Proc Natl Acad Sci U S A. 2020;117(20):10970-10975. doi:10.1073/pnas.2005615117

98. Duan K, Liu B, Li C, et al. Effectiveness of convalescent plasma therapy in severe COVID-19 patients. Proc Natl Acad Sci U S A. 2020;117(17):9490-9496. doi:10.1073/pnas.2004168117

99. Mosenzon O, Wiviott SD, Cahn A, et al. Effects of dapagliflozin on development and progression of kidney disease in patients with type 2 diabetes: an analysis from the DECLARE–TIMI 58 randomised trial. Lancet Diabetes Endocrinol. 2019;7(8):606-617. doi:10.1016/S2213-8587(19)30180-9

100. Roberts I, Shakur-Still H, Aeron-Thomas A, et al. Effects of tranexamic acid on death, disability, vascular occlusive events and other morbidities in patients with acute traumatic brain injury (CRASH-3): A randomised, placebo-controlled trial. Lancet. 2019;394(10210):1713-1723. doi:10.1016/S0140-6736(19)32233-0

101. Tanriover MD, Doğanay HL, Akova M, et al. Efficacy and safety of an inactivated whole-virion SARS-CoV-2 vaccine (CoronaVac): interim results of a double-blind, randomised, placebo-controlled, phase 3 trial in Turkey. Lancet. 2021;398(10296):213-222. doi:10.1016/S0140-6736(21)01429-X

102. Bachert C, Han JK, Desrosiers M, et al. Efficacy and safety of dupilumab in patients with severe chronic rhinosinusitis with nasal polyps (LIBERTY NP SINUS-24 and LIBERTY NP SINUS-52): results from two multicentre, randomised, double-blind, placebo-controlled, parallel-group phase 3 trials. Lancet. 2019;394(10209):1638-1650. doi:10.1016/S0140-6736(19)31881-1

103. Tardif JC, Kouz S, Waters DD, et al. Efficacy and Safety of Low-Dose Colchicine after Myocardial Infarction. N Engl J Med. 2019;381(26):2497-2505. doi:10.1056/nejmoa1912388

104. Hanley DF, Thompson RE, Rosenblum M, et al. Efficacy and safety of minimally invasive surgery with thrombolysis in intracerebral haemorrhage evacuation (MISTIE III): a randomised, controlled, open-label, blinded endpoint phase 3 trial. Lancet. 2019;393(10175):1021-1032. doi:10.1016/S0140-6736(19)30195-3

105. Yau T, Kang YK, Kim TY, et al. Efficacy and Safety of Nivolumab plus Ipilimumab in Patients with Advanced Hepatocellular Carcinoma Previously Treated with Sorafenib: The CheckMate 040 Randomized Clinical Trial. JAMA Oncol. 2020;6(11). doi:10.1001/jamaoncol.2020.4564

106. Chung HC, Ros W, Delord JP, et al. Efficacy and safety of pembrolizumab in previously treated advanced cervical cancer: Results from the phase II KEYNOTE-158 study. J Clin Oncol. 2019;37(17):1470-1478. doi:10.1200/JCO.18.01265

107. Heijerman HGM, McKone EF, Downey DG, et al. Efficacy and safety of the elexacaftor plus tezacaftor plus ivacaftor combination regimen in people with cystic fibrosis homozygous for the F508del mutation: a

© 2023 Siena LM et al. *JAMA Network Open.*

double-blind, randomised, phase 3 trial. Lancet. 2019;394(10212):1940-1948. doi:10.1016/S0140-6736(19)32597-8

108. Baden LR, El Sahly HM, Essink B, et al. Efficacy and Safety of the mRNA-1273 SARS-CoV-2 Vaccine. N Engl J Med. 2021;384(5):403-416. doi:10.1056/nejmoa2035389

109. Jault P, Leclerc T, Jennes S, et al. Efficacy and tolerability of a cocktail of bacteriophages to treat burn wounds infected by Pseudomonas aeruginosa (PhagoBurn): a randomised, controlled, double-blind phase 1/2 trial. Lancet Infect Dis. 2019;19(1):35-45. doi:10.1016/S1473-3099(18)30482-1

110. Marabelle A, Le DT, Ascierto PA, et al. Efficacy of pembrolizumab in patients with noncolorectal high microsatellite instability/ mismatch repair–deficient cancer: Results from the phase II KEYNOTE-158 study. J Clin Oncol. 2020;38(1):1-10. doi:10.1200/JCO.19.02105

111. Herishanu Y, Avivi I, Aharon A, et al. Efficacy of the BNT162b2 mRNA COVID-19 vaccine in patients with chronic lymphocytic leukemia. Blood. 2021;137(23):3165-3173. doi:10.1182/blood.2021011568

112. Madhi SA, Baillie V, Cutland CL, et al. Efficacy of the ChAdOx1 nCoV-19 Covid-19 Vaccine against the B.1.351 Variant. N Engl J Med. 2021;384(20):1885-1898. doi:10.1056/nejmoa2102214

113. Stone JH, Frigault MJ, Serling-Boyd NJ, et al. Efficacy of Tocilizumab in Patients Hospitalized with Covid-19. N Engl J Med. 2020;383(24):2333-2344. doi:10.1056/nejmoa2028836

114. Middleton PG, Mall MA, Dřevínek P, et al. Elexacaftor–Tezacaftor–Ivacaftor for Cystic Fibrosis with a Single Phe508del Allele. N Engl J Med. 2019;381(19):1809-1819. doi:10.1056/nejmoa1908639

115. Anker SD, Butler J, Filippatos G, et al. Empagliflozin in Heart Failure with a Preserved Ejection Fraction. N Engl J Med. 2021;385(16):1451-1461. doi:10.1056/nejmoa2107038

116. Kopetz S, Grothey A, Yaeger R, et al. Encorafenib, Binimetinib, and Cetuximab in BRAF V600E–Mutated Colorectal Cancer . N Engl J Med. 2019;381(17):1632-1643. doi:10.1056/nejmoa1908075

117. Davis ID, Martin AJ, Stockler MR, et al. Enzalutamide with Standard First-Line Therapy in Metastatic Prostate Cancer. N Engl J Med. 2019;381(2):121-131. doi:10.1056/nejmoa1903835

118. Long G V., Dummer R, Hamid O, et al. Epacadostat plus pembrolizumab versus placebo plus pembrolizumab in patients with unresectable or metastatic melanoma (ECHO-301/KEYNOTE-252): a phase 3, randomised, double-blind study. Lancet Oncol. 2019;20(8):1083-1097. doi:10.1016/S1470-2045(19)30274-8

119. Loriot Y, Necchi A, Park SH, et al. Erdafitinib in Locally Advanced or Metastatic Urothelial Carcinoma. N Engl J Med. 2019;381(4):338-348. doi:10.1056/nejmoa1817323

120. Saito H, Fukuhara T, Furuya N, et al. Erlotinib plus bevacizumab versus erlotinib alone in patients with EGFR-positive advanced non-squamous non-small-cell lung cancer (NEJ026): interim analysis of an open-label, randomised, multicentre, phase 3 trial. Lancet Oncol. 2019;20(5):625-635. doi:10.1016/S1470-2045(19)30035-X

121. Mathew MG, Samuel SR, Soni AJ, Roopa KB. Evaluation of adhesion of Streptococcus mutans, plaque accumulation on zirconia and stainless steel crowns, and surrounding gingival inflammation in primary molars: randomized controlled trial. Clin Oral Investig. 2020;24(9):3275-3280. doi:10.1007/s00784-020-03204-9

122. Davar D, Dzutsev AK, McCulloch JA, et al. Fecal microbiota transplant overcomes resistance to anti-PD-1 therapy in melanoma patients. Science (80-). 2021;371(6529):595-602. doi:10.1126/science.abf3363

© 2023 Siena LM et al. *JAMA Network Open.*

123. Baruch EN, Youngster I, Ben-Betzalel G, et al. Fecal microbiota transplant promotes response in immunotherapy-refractory melanoma patients. Science (80-). 2021;371(6529):602-609. doi:10.1126/science.abb5920

124. Janjigian YY, Shitara K, Moehler M, et al. First-line nivolumab plus chemotherapy versus chemotherapy alone for advanced gastric, gastro-oesophageal junction, and oesophageal adenocarcinoma (CheckMate 649): a randomised, open-label, phase 3 trial. Lancet. 2021;398(10294):27-40. doi:10.1016/S0140-6736(21)00797-2

125. Paz-Ares L, Ciuleanu TE, Cobo M, et al. First-line nivolumab plus ipilimumab combined with two cycles of chemotherapy in patients with non-small-cell lung cancer (CheckMate 9LA): an international, randomised, open-label, phase 3 trial. Lancet Oncol. 2021;22(2):198-211. doi:10.1016/S1470-2045(20)30641-0

126. Baas P, Scherpereel A, Nowak AK, et al. First-line nivolumab plus ipilimumab in unresectable malignant pleural mesothelioma (CheckMate 743): a multicentre, randomised, open-label, phase 3 trial. Lancet. 2021;397(10272):375-386. doi:10.1016/S0140-6736(20)32714-8

127. Stone GW, Kappetein AP, Sabik JF, et al. Five-Year Outcomes after PCI or CABG for Left Main Coronary Disease. N Engl J Med. 2019;381(19):1820-1830. doi:10.1056/nejmoa1909406

128. Garon EB, Hellmann MD, Rizvi NA, et al. Five-year overall survival for patients with advanced non-small-cell lung cancer treated with pembrolizumab: Results from the phase i KEYNOTE-001 study. J Clin Oncol. 2019;37(28):2518-2527. doi:10.1200/JCO.19.00934

129. Hamid O, Robert C, Daud A, et al. Five-year survival outcomes for patients with advanced melanoma treated with pembrolizumab in KEYNOTE-001. Ann Oncol. 2019;30(4):582-588. doi:10.1093/annonc/mdz011

130. Larkin J, Chiarion-Sileni V, Gonzalez R, et al. Five-Year Survival with Combined Nivolumab and Ipilimumab in Advanced Melanoma. N Engl J Med. 2019;381(16):1535-1546. doi:10.1056/nejmoa1910836

131. Zhang Y, Chen L, Hu GQ, et al. Gemcitabine and Cisplatin Induction Chemotherapy in Nasopharyngeal Carcinoma. N Engl J Med. 2019;381(12):1124-1135. doi:10.1056/nejmoa1905287

132. Perl AE, Martinelli G, Cortes JE, et al. Gilteritinib or Chemotherapy for Relapsed or Refractory FLT3 - Mutated AML . N Engl J Med. 2019;381(18):1728-1740. doi:10.1056/nejmoa1902688

133. Rastinehad AR, Anastos H, Wajswol E, et al. Gold nanoshell-localized photothermal ablation of prostate tumors in a clinical pilot device study. Proc Natl Acad Sci U S A. 2019;116(37):18590-18596. doi:10.1073/pnas.1906929116

134. van der Leest M, Cornel E, Israël B, et al. Head-to-head Comparison of Transrectal Ultrasound-guided Prostate Biopsy Versus Multiparametric Prostate Resonance Imaging with Subsequent Magnetic Resonance-guided Biopsy in Biopsy-naïve Men with Elevated Prostate-specific Antigen: A Large Prospective Multicenter Clinical Study(Figure presented.). Eur Urol. 2019;75(4):570-578. doi:10.1016/j.eururo.2018.11.023

135. Mariette C, Markar SR, Dabakuyo-Yonli TS, et al. Hybrid Minimally Invasive Esophagectomy for Esophageal Cancer. N Engl J Med. 2019;380(2):152-162. doi:10.1056/nejmoa1805101

136. Gautret P, Lagier JC, Parola P, et al. Hydroxychloroquine and azithromycin as a treatment of COVID-19: results of an open-label non-randomized clinical trial. Int J Antimicrob Agents. 2020;56(1). doi:10.1016/j.ijantimicag.2020.105949

© 2023 Siena LM et al. *JAMA Network Open.*

137. Tang W, Cao Z, Han M, et al. Hydroxychloroquine in patients with mainly mild to moderate coronavirus disease 2019: Open label, randomised controlled trial. BMJ. 2020;369. doi:10.1136/bmj.m1849

138. Cavalcanti AB, Zampieri FG, Rosa RG, et al. Hydroxychloroquine with or without Azithromycin in Mild-to-Moderate Covid-19. N Engl J Med. 2020;383(21):2041-2052. doi:10.1056/nejmoa2019014

139. Murray Brunt A, Haviland JS, Wheatley DA, et al. Hypofractionated breast radiotherapy for 1 week versus 3 weeks (FAST-Forward): 5-year efficacy and late normal tissue effects results from a multicentre, non-inferiority, randomised, phase 3 trial. Lancet. 2020;395(10237):1613-1626. doi:10.1016/S0140-6736(20)30932-6

140. Jain N, Keating M, Thompson P, et al. Ibrutinib and Venetoclax for First-Line Treatment of CLL. N Engl J Med. 2019;380(22):2095-2103. doi:10.1056/nejmoa1900574

141. Moreno C, Greil R, Demirkan F, et al. Ibrutinib plus obinutuzumab versus chlorambucil plus obinutuzumab in first-line treatment of chronic lymphocytic leukaemia (iLLUMINATE): a multicentre, randomised, open-label, phase 3 trial. Lancet Oncol. 2019;20(1):43-56. doi:10.1016/S1470-2045(18)30788-5

142. Shanafelt TD, Wang X V., Kay NE, et al. Ibrutinib–Rituximab or Chemoimmunotherapy for Chronic Lymphocytic Leukemia. N Engl J Med. 2019;381(5):432-443. doi:10.1056/nejmoa1817073

143. Munshi NC, Anderson LD, Shah N, et al. Idecabtagene Vicleucel in Relapsed and Refractory Multiple Myeloma. N Engl J Med. 2021;384(8):705-716. doi:10.1056/nejmoa2024850

144. Voorwerk L, Slagter M, Horlings HM, et al. Immune induction strategies in metastatic triple-negative breast cancer to enhance the sensitivity to PD-1 blockade: the TONIC trial. Nat Med. 2019;25(6):920-928. doi:10.1038/s41591-019-0432-4

145. Zhu FC, Guan XH, Li YH, et al. Immunogenicity and safety of a recombinant adenovirus type-5-vectored COVID-19 vaccine in healthy adults aged 18 years or older: a randomised, double-blind, placebo-controlled, phase 2 trial. Lancet. 2020;396(10249):479-488. doi:10.1016/S0140-6736(20)31605-6

146. Schuetz P, Fehr R, Baechli V, et al. Individualised nutritional support in medical inpatients at nutritional risk: a randomised clinical trial. Lancet. 2019;393(10188):2312-2321. doi:10.1016/S0140-6736(18)32776-4

147. Maron DJ, Hochman JS, Reynolds HR, et al. Initial Invasive or Conservative Strategy for Stable Coronary Disease. N Engl J Med. 2020;382(15):1395-1407. doi:10.1056/nejmoa1915922

148. Sadoff J, Le Gars M, Shukarev G, et al. Interim Results of a Phase 1–2a Trial of Ad26.COV2.S Covid-19 Vaccine. N Engl J Med. 2021;384(19):1824-1835. doi:10.1056/nejmoa2034201

149. Interleukin-6 Receptor Antagonists in Critically Ill Patients with Covid-19. N Engl J Med. 2021;384(16):1491-1502. doi:10.1056/nejmoa2100433

150. Attal M, Richardson PG, Rajkumar SV, et al. Isatuximab plus pomalidomide and low-dose dexamethasone versus pomalidomide and low-dose dexamethasone in patients with relapsed and refractory multiple myeloma (ICARIA-MM): a randomised, multicentre, open-label, phase 3 study. Lancet. 2019;394(10214):2096-2107. doi:10.1016/S0140-6736(19)32556-5

151. Abou-Alfa GK, Macarulla T, Javle MM, et al. Ivosidenib in IDH1-mutant, chemotherapy-refractory cholangiocarcinoma (ClarIDHy): a multicentre, randomised, double-blind, placebo-controlled, phase 3 study. Lancet Oncol. 2020;21(6):796-807. doi:10.1016/S1470-2045(20)30157-1

152. Hong DS, Fakih MG, Strickler JH, et al. KRAS G12C Inhibition with Sotorasib in Advanced Solid Tumors . N Engl J Med. 2020;383(13):1207-1217. doi:10.1056/nejmoa1917239

© 2023 Siena LM et al. *JAMA Network Open.*

153. Wang M, Munoz J, Goy A, et al. KTE-X19 CAR T-Cell Therapy in Relapsed or Refractory Mantle-Cell Lymphoma. N Engl J Med. 2020;382(14):1331-1342. doi:10.1056/nejmoa1914347

154. Perez M V., Mahaffey KW, Hedlin H, et al. Large-Scale Assessment of a Smartwatch to Identify Atrial Fibrillation. N Engl J Med. 2019;381(20):1909-1917. doi:10.1056/nejmoa1901183

155. Motzer R, Alekseev B, Rha SY, et al. Lenvatinib plus Pembrolizumab or Everolimus for Advanced Renal Cell Carcinoma. N Engl J Med. 2021;384(14):1289-1300. doi:10.1056/nejmoa2035716

156. O'Donoghue ML, Fazio S, Giugliano RP, et al. Lipoprotein(a), PCSK9 inhibition, and cardiovascular risk insights from the FOURIER trial. Circulation. 2019;139(12):1483-1492. doi:10.1161/CIRCULATIONAHA.118.037184

157. Abramson JS, Palomba ML, Gordon LI, et al. Lisocabtagene maraleucel for patients with relapsed or refractory large B-cell lymphomas (TRANSCEND NHL 001): a multicentre seamless design study. Lancet. 2020;396(10254):839-852. doi:10.1016/S0140-6736(20)31366-0

158. Gomez DR, Tang C, Zhang J, et al. Local consolidative therapy vs. Maintenance therapy or observation for patients with oligometastatic non–small-cell lung cancer: Long-term results of a multi-institutional, phase II, randomized study. J Clin Oncol. 2019;37(18):1558-1565. doi:10.1200/JCO.19.00201

159. Emens LA, Cruz C, Eder JP, et al. Long-term Clinical Outcomes and Biomarker Analyses of Atezolizumab Therapy for Patients with Metastatic Triple-Negative Breast Cancer: A Phase 1 Study. JAMA Oncol. 2019;5(1):74-82. doi:10.1001/jamaoncol.2018.4224

160. Locke FL, Ghobadi A, Jacobson CA, et al. Long-term safety and activity of axicabtagene ciloleucel in refractory large B-cell lymphoma (ZUMA-1): a single-arm, multicentre, phase 1–2 trial. Lancet Oncol. 2019;20(1):31-42. doi:10.1016/S1470-2045(18)30864-7

161. Horby PW, Mafham M, Bell JL, et al. Lopinavir–ritonavir in patients admitted to hospital with COVID-19 (RECOVERY): a randomised, controlled, open-label, platform trial. Lancet. 2020;396(10259):1345-1352. doi:10.1016/S0140-6736(20)32013-4

162. Ridker PM, Everett BM, Pradhan A, et al. Low-Dose Methotrexate for the Prevention of Atherosclerotic Events. N Engl J Med. 2019;380(8):752-762. doi:10.1056/nejmoa1809798

163. Sartor O, de Bono J, Chi KN, et al. Lutetium-177–PSMA-617 for Metastatic Castration-Resistant Prostate Cancer. N Engl J Med. 2021;385(12):1091-1103. doi:10.1056/nejmoa2107322

164. Golan T, Hammel P, Reni M, et al. Maintenance Olaparib for Germline BRCA -Mutated Metastatic Pancreatic Cancer . N Engl J Med. 2019;381(4):317-327. doi:10.1056/nejmoa1903387

165. Manson JE, Cook NR, Lee IM, et al. Marine n−3 Fatty Acids and Prevention of Cardiovascular Disease and Cancer. N Engl J Med. 2019;380(1):23-32. doi:10.1056/nejmoa1811403

166. Jayk Bernal A, Gomes da Silva MM, Musungaie DB, et al. Molnupiravir for Oral Treatment of Covid-19 in Nonhospitalized Patients. N Engl J Med. 2022;386(6):509-520. doi:10.1056/nejmoa2116044

167. Ahdoot M, Wilbur AR, Reese SE, et al. MRI-Targeted, Systematic, and Combined Biopsy for Prostate Cancer Diagnosis. N Engl J Med. 2020;382(10):917-928. doi:10.1056/nejmoa1910038

168. Cloughesy TF, Mochizuki AY, Orpilla JR, et al. Neoadjuvant anti-PD-1 immunotherapy promotes a survival benefit with intratumoral and systemic immune responses in recurrent glioblastoma. Nat Med. 2019;25(3):477-486. doi:10.1038/s41591-018-0337-7

© 2023 Siena LM et al. *JAMA Network Open.*

169. Mittendorf EA, Zhang H, Barrios CH, et al. Neoadjuvant atezolizumab in combination with sequential nab-paclitaxel and anthracycline-based chemotherapy versus placebo and chemotherapy in patients with early-stage triple-negative breast cancer (IMpassion031): a randomised, double-blind, phase 3 trial. Lancet. 2020;396(10257):1090-1100. doi:10.1016/S0140-6736(20)31953-X

170. Chalabi M, Fanchi LF, Dijkstra KK, et al. Neoadjuvant immunotherapy leads to pathological responses in MMR-proficient and MMR-deficient early-stage colon cancers. Nat Med. 2020;26(4):566-576. doi:10.1038/s41591-020-0805-8

171. Schalper KA, Rodriguez-Ruiz ME, Diez-Valle R, et al. Neoadjuvant nivolumab modifies the tumor immune microenvironment in resectable glioblastoma. Nat Med. 2019;25(3):470-476. doi:10.1038/s41591-018-0339-5

172. Keskin DB, Anandappa AJ, Sun J, et al. Neoantigen vaccine generates intratumoral T cell responses in phase Ib glioblastoma trial. Nature. 2019;565(7738):234-239. doi:10.1038/s41586-018-0792-9

173. Distler O, Highland KB, Gahlemann M, et al. Nintedanib for Systemic Sclerosis–Associated Interstitial Lung Disease. N Engl J Med. 2019;380(26):2518-2528. doi:10.1056/nejmoa1903076

174. Flaherty KR, Wells AU, Cottin V, et al. Nintedanib in Progressive Fibrosing Interstitial Lung Diseases. N Engl J Med. 2019;381(18):1718-1727. doi:10.1056/nejmoa1908681

175. González-Martín A, Pothuri B, Vergote I, et al. Niraparib in Patients with Newly Diagnosed Advanced Ovarian Cancer. N Engl J Med. 2019;381(25):2391-2402. doi:10.1056/nejmoa1910962

176. Choueiri TK, Powles T, Burotto M, et al. Nivolumab plus Cabozantinib versus Sunitinib for Advanced Renal-Cell Carcinoma. N Engl J Med. 2021;384(9):829-841. doi:10.1056/nejmoa2026982

177. Hellmann MD, Paz-Ares L, Bernabe Caro R, et al. Nivolumab plus Ipilimumab in Advanced Non–Small-Cell Lung Cancer. N Engl J Med. 2019;381(21):2020-2031. doi:10.1056/nejmoa1910231

178. Motzer RJ, Rini BI, McDermott DF, et al. Nivolumab plus ipilimumab versus sunitinib in first-line treatment for advanced renal cell carcinoma: extended follow-up of efficacy and safety results from a randomised, controlled, phase 3 trial. Lancet Oncol. 2019;20(10):1370-1385. doi:10.1016/S1470-2045(19)30413-9

179. Kato K, Cho BC, Takahashi M, et al. Nivolumab versus chemotherapy in patients with advanced oesophageal squamous cell carcinoma refractory or intolerant to previous chemotherapy (ATTRACTION-3): a multicentre, randomised, open-label, phase 3 trial. Lancet Oncol. 2019;20(11):1506-1517. doi:10.1016/S1470-2045(19)30626-6

180. Younossi ZM, Ratziu V, Loomba R, et al. Obeticholic acid for the treatment of non-alcoholic steatohepatitis: interim analysis from a multicentre, randomised, placebo-controlled phase 3 trial. Lancet. 2019;394(10215):2184-2196. doi:10.1016/S0140-6736(19)33041-7

181. de Bono J, Mateo J, Fizazi K, et al. Olaparib for Metastatic Castration-Resistant Prostate Cancer. N Engl J Med. 2020;382(22):2091-2102. doi:10.1056/nejmoa1911440

182. Ray-Coquard I, Pautier P, Pignata S, et al. Olaparib plus Bevacizumab as First-Line Maintenance in Ovarian Cancer. N Engl J Med. 2019;381(25):2416-2428. doi:10.1056/nejmoa1911361

183. Wilding JPH, Batterham RL, Calanna S, et al. Once-Weekly Semaglutide in Adults with Overweight or Obesity. N Engl J Med. 2021;384(11):989-1002. doi:10.1056/nejmoa2032183

184. Chari A, Vogl DT, Gavriatopoulou M, et al. Oral Selinexor–Dexamethasone for Triple-Class Refractory Multiple Myeloma. N Engl J Med. 2019;381(8):727-738. doi:10.1056/nejmoa1903455

© 2023 Siena LM et al. *JAMA Network Open.*

185. Husain M, Birkenfeld AL, Donsmark M, et al. Oral Semaglutide and Cardiovascular Outcomes in Patients with Type 2 Diabetes. N Engl J Med. 2019;381(9):841-851. doi:10.1056/nejmoa1901118

186. Wu YL, Tsuboi M, He J, et al. Osimertinib in Resected EGFR -Mutated Non–Small-Cell Lung Cancer . N Engl J Med. 2020;383(18):1711-1723. doi:10.1056/nejmoa2027071

187. Phillips R, Shi WY, Deek M, et al. Outcomes of Observation vs Stereotactic Ablative Radiation for Oligometastatic Prostate Cancer: The ORIOLE Phase 2 Randomized Clinical Trial. JAMA Oncol. 2020;6(5):650-659. doi:10.1001/jamaoncol.2020.0147

188. Ramalingam SS, Vansteenkiste J, Planchard D, et al. Overall Survival with Osimertinib in Untreated, EGFR -Mutated Advanced NSCLC . N Engl J Med. 2020;382(1):41-50. doi:10.1056/nejmoa1913662

189. Im SA, Lu YS, Bardia A, et al. Overall Survival with Ribociclib plus Endocrine Therapy in Breast Cancer. N Engl J Med. 2019;381(4):307-316. doi:10.1056/nejmoa1903765

190. Slamon DJ, Neven P, Chia S, et al. Overall Survival with Ribociclib plus Fulvestrant in Advanced Breast Cancer. N Engl J Med. 2020;382(6):514-524. doi:10.1056/nejmoa1911149

191. van den Brink R, Lommerse I. ‘Uitbehandelen’ endocarditis kan ook oraal. Ned Tijdschr Geneeskd. 2019;163(15). doi:10.1056/NEJMoa1808312

192. Burtness B, Harrington KJ, Greil R, et al. Pembrolizumab alone or with chemotherapy versus cetuximab with chemotherapy for recurrent or metastatic squamous cell carcinoma of the head and neck (KEYNOTE-048): a randomised, open-label, phase 3 study. Lancet. 2019;394(10212):1915-1928. doi:10.1016/S0140-6736(19)32591-7

193. Finn RS, Ryoo BY, Merle P, et al. Pembrolizumab As Second-Line Therapy in Patients With Advanced Hepatocellular Carcinoma in KEYNOTE-240: A Randomized, Double-Blind, Phase III Trial. J Clin Oncol. 2020;38(3):193-202. doi:10.1200/JCO.19.01307

194. Schmid P, Cortes J, Pusztai L, et al. Pembrolizumab for Early Triple-Negative Breast Cancer. N Engl J Med. 2020;382(9):810-821. doi:10.1056/nejmoa1910549

195. André T, Shiu KK, Kim TW, et al. Pembrolizumab in Microsatellite-Instability–High Advanced Colorectal Cancer. N Engl J Med. 2020;383(23):2207-2218. doi:10.1056/nejmoa2017699

196. Adams S, Schmid P, Rugo HS, et al. Pembrolizumab monotherapy for previously treated metastatic triple-negative breast cancer: Cohort A of the phase II KEYNOTE-086 study. Ann Oncol. 2019;30(3):397-404. doi:10.1093/annonc/mdy517

197. Adams S, Loi S, Toppmeyer D, et al. Pembrolizumab monotherapy for previously untreated, PD-L1-positive, metastatic triple-negative breast cancer: Cohort B of the phase II KEYNOTE-086 study. Ann Oncol. 2019;30(3):405-411. doi:10.1093/annonc/mdy518

198. Rini BI, Plimack ER, Stus V, et al. Pembrolizumab plus Axitinib versus Sunitinib for Advanced Renal-Cell Carcinoma. N Engl J Med. 2019;380(12):1116-1127. doi:10.1056/nejmoa1816714

199. Cortes J, Cescon DW, Rugo HS, et al. Pembrolizumab plus chemotherapy versus placebo plus chemotherapy for previously untreated locally recurrent inoperable or metastatic triple-negative breast cancer (KEYNOTE-355): a randomised, placebo-controlled, double-blind, phase 3 clinical trial. Lancet. 2020;396(10265):1817-1828. doi:10.1016/S0140-6736(20)32531-9

200. Mok TSK, Wu YL, Kudaba I, et al. Pembrolizumab versus chemotherapy for previously untreated, PD-L1-expressing, locally advanced or metastatic non-small-cell lung cancer (KEYNOTE-042): a randomised,

© 2023 Siena LM et al. *JAMA Network Open.*

open-label, controlled, phase 3 trial. Lancet. 2019;393(10183):1819-1830. doi:10.1016/S0140-6736(18)32409-7

201. Robert C, Ribas A, Schachter J, et al. Pembrolizumab versus ipilimumab in advanced melanoma (KEYNOTE-006): post-hoc 5-year results from an open-label, multicentre, randomised, controlled, phase 3 study. Lancet Oncol. 2019;20(9):1239-1251. doi:10.1016/S1470-2045(19)30388-2

202. Cohen EEW, Soulières D, Le Tourneau C, et al. Pembrolizumab versus methotrexate, docetaxel, or cetuximab for recurrent or metastatic head-and-neck squamous cell carcinoma (KEYNOTE-040): a randomised, open-label, phase 3 study. Lancet. 2019;393(10167):156-167. doi:10.1016/S0140-6736(18)31999-8

203. Abou-Alfa GK, Sahai V, Hollebecque A, et al. Pemigatinib for previously treated, locally advanced or metastatic cholangiocarcinoma: a multicentre, open-label, phase 2 study. Lancet Oncol. 2020;21(5):671-684. doi:10.1016/S1470-2045(20)30109-1

204. Al-Batran SE, Homann N, Pauligk C, et al. Perioperative chemotherapy with fluorouracil plus leucovorin, oxaliplatin, and docetaxel versus fluorouracil or capecitabine plus cisplatin and epirubicin for locally advanced, resectable gastric or gastro-oesophageal junction adenocarcinoma (FLOT4): a randomised, phase 2/3 trial. Lancet. 2019;393(10184):1948-1957. doi:10.1016/S0140-6736(18)32557-1

205. Keech C, Albert G, Cho I, et al. Phase 1–2 Trial of a SARS-CoV-2 Recombinant Spike Protein Nanoparticle Vaccine. N Engl J Med. 2020;383(24):2320-2332. doi:10.1056/nejmoa2026920

206. Mulligan MJ, Lyke KE, Kitchin N, et al. Phase I/II study of COVID-19 RNA vaccine BNT162b1 in adults. Nature. 2020;586(7830):589-593. doi:10.1038/s41586-020-2639-4

207. Finn RS, Ikeda M, Zhu AX, et al. Phase ib study of lenvatinib plus pembrolizumab in patients with unresectable hepatocellular carcinoma. J Clin Oncol. 2020;38(26):2960-2970. doi:10.1200/JCO.20.00808

208. Le DT, Kim TW, van Cutsem E, et al. Phase II open-label study of pembrolizumab in treatment-refractory, microsatellite instability–high/mismatch repair–deficient metastatic colorectal cancer: KEYNOTE-164. J Clin Oncol. 2020;38(1):11-19. doi:10.1200/JCO.19.02107

209. Sehn LH, Herrera AF, Flowers CR, et al. Polatuzumab vedotin in relapsed or refractory diffuse large B-cell lymphoma. J Clin Oncol. 2020;38(2):155-165. doi:10.1200/JCO.19.00172

210. Versteijne E, Suker M, Groothuis K, et al. Preoperative Chemoradiotherapy Versus Immediate Surgery for Resectable and Borderline Resectable Pancreatic Cancer: Results of the Dutch Randomized Phase III PREOPANC Trial. J Clin Oncol. 2020;38(16):1763-1773. doi:10.1200/JCO.19.02274

211. Hofman MS, Lawrentschuk N, Francis RJ, et al. Prostate-specific membrane antigen PET-CT in patients with high-risk prostate cancer before curative-intent surgery or radiotherapy (proPSMA): a prospective, randomised, multicentre study. Lancet. 2020;395(10231):1208-1216. doi:10.1016/S0140-6736(20)30314-7

212. Gillison ML, Trotti AM, Harris J, et al. Radiotherapy plus cetuximab or cisplatin in human papillomavirus-positive oropharyngeal cancer (NRG Oncology RTOG 1016): a randomised, multicentre, non-inferiority trial. Lancet. 2019;393(10166):40-50. doi:10.1016/S0140-6736(18)32779-X

213. Mehanna H, Robinson M, Hartley A, et al. Radiotherapy plus cisplatin or cetuximab in low-risk human papillomavirus-positive oropharyngeal cancer (De-ESCALaTE HPV): an open-label randomised controlled phase 3 trial. Lancet. 2019;393(10166):51-60. doi:10.1016/S0140-6736(18)32752-1

© 2023 Siena LM et al. *JAMA Network Open.*

214. Zhu AX, Kang YK, Yen CJ, et al. Ramucirumab after sorafenib in patients with advanced hepatocellular carcinoma and increased α-fetoprotein concentrations (REACH-2): a randomised, double-blind, placebo-controlled, phase 3 trial. Lancet Oncol. 2019;20(2):282-296. doi:10.1016/S1470-2045(18)30937-9

215. Nakagawa K, Garon EB, Seto T, et al. Ramucirumab plus erlotinib in patients with untreated, EGFR-mutated, advanced non-small-cell lung cancer (RELAY): a randomised, double-blind, placebo-controlled, phase 3 trial. Lancet Oncol. 2019;20(12):1655-1669. doi:10.1016/S1470-2045(19)30634-5

216. Kojima T, Shah MA, Muro K, et al. Randomized phase III KEYNOTE-181 study of pembrolizumab versus chemotherapy in advanced esophageal cancer. J Clin Oncol. 2020;38(35):4138-4148. doi:10.1200/JCO.20.01888

217. Wang P, Berzin TM, Glissen Brown JR, et al. Real-time automatic detection system increases colonoscopic polyp and adenoma detection rates: A prospective randomised controlled study. Gut. 2019;68(10):1813-1819. doi:10.1136/gutjnl-2018-317500

218. de Koning HJ, van der Aalst CM, de Jong PA, et al. Reduced Lung-Cancer Mortality with Volume CT Screening in a Randomized Trial. N Engl J Med. 2020;382(6):503-513. doi:10.1056/nejmoa1911793

219. Weinreich DM, Sivapalasingam S, Norton T, et al. REGN-COV2, a Neutralizing Antibody Cocktail, in Outpatients with Covid-19. N Engl J Med. 2021;384(3):238-251. doi:10.1056/nejmoa2035002

220. Goldman JD, Lye DCB, Hui DS, et al. Remdesivir for 5 or 10 Days in Patients with Severe Covid-19. N Engl J Med. 2020;383(19):1827-1837. doi:10.1056/nejmoa2015301

221. Beigel JH, Tomashek KM, Dodd LE, et al. Remdesivir for the Treatment of Covid-19 — Final Report. N Engl J Med. 2020;383(19):1813-1826. doi:10.1056/nejmoa2007764

222. Wang Y, Zhang D, Du G, et al. Remdesivir in adults with severe COVID-19: a randomised, double-blind, placebo-controlled, multicentre trial. Lancet. 2020;395(10236):1569-1578. doi:10.1016/S0140-6736(20)31022-9

223. Repurposed Antiviral Drugs for Covid-19 — Interim WHO Solidarity Trial Results. N Engl J Med. 2021;384(6):497-511. doi:10.1056/nejmoa2023184

224. Khorana AA, Soff GA, Kakkar AK, et al. Rivaroxaban for Thromboprophylaxis in High-Risk Ambulatory Patients with Cancer. N Engl J Med. 2019;380(8):720-728. doi:10.1056/nejmoa1814630

225. van der Sluis PC, van der Horst S, May AM, et al. Robot-assisted Minimally Invasive Thoracolaparoscopic Esophagectomy Versus Open Transthoracic Esophagectomy for Resectable Esophageal Cancer: A Randomized Controlled Trial. Ann Surg. 2019;269(4):621-630. doi:10.1097/SLA.0000000000003031

226. Bardia A, Mayer IA, Vahdat LT, et al. Sacituzumab Govitecan-hziy in Refractory Metastatic Triple-Negative Breast Cancer. N Engl J Med. 2019;380(8):741-751. doi:10.1056/nejmoa1814213

227. Logunov DY, Dolzhikova I V., Shcheblyakov D V., et al. Safety and efficacy of an rAd26 and rAd5 vector-based heterologous prime-boost COVID-19 vaccine: an interim analysis of a randomised controlled phase 3 trial in Russia. Lancet. 2021;397(10275):671-681. doi:10.1016/S0140-6736(21)00234-8

228. Ray KK, Bays HE, Catapano AL, et al. Safety and Efficacy of Bempedoic Acid to Reduce LDL Cholesterol. N Engl J Med. 2019;380(11):1022-1032. doi:10.1056/nejmoa1803917

229. Heath PT, Galiza EP, Baxter DN, et al. Safety and Efficacy of NVX-CoV2373 Covid-19 Vaccine. N Engl J Med. 2021;385(13):1172-1183. doi:10.1056/nejmoa2107659

© 2023 Siena LM et al. *JAMA Network Open.*

230. Sadoff J, Gray G, Vandebosch A, et al. Safety and Efficacy of Single-Dose Ad26.COV2.S Vaccine against Covid-19. N Engl J Med. 2021;384(23):2187-2201. doi:10.1056/nejmoa2101544

231. Polack FP, Thomas SJ, Kitchin N, et al. Safety and Efficacy of the BNT162b2 mRNA Covid-19 Vaccine. N Engl J Med. 2020;383(27):2603-2615. doi:10.1056/nejmoa2034577

232. Thomas SJ, Moreira ED, Kitchin N, et al. Safety and Efficacy of the BNT162b2 mRNA Covid-19 Vaccine through 6 Months. N Engl J Med. 2021;385(19):1761-1773. doi:10.1056/nejmoa2110345

233. Xia S, Zhang Y, Wang Y, et al. Safety and immunogenicity of an inactivated SARS-CoV-2 vaccine, BBIBP-CorV: a randomised, double-blind, placebo-controlled, phase 1/2 trial. Lancet Infect Dis. 2021;21(1):39-51. doi:10.1016/S1473-3099(20)30831-8

234. Logunov DY, Dolzhikova I V., Zubkova O V., et al. Safety and immunogenicity of an rAd26 and rAd5 vector-based heterologous prime-boost COVID-19 vaccine in two formulations: two open, non-randomised phase 1/2 studies from Russia. Lancet. 2020;396(10255):887-897. doi:10.1016/S0140-6736(20)31866-3

235. Ramasamy MN, Minassian AM, Ewer KJ, et al. Safety and immunogenicity of ChAdOx1 nCoV-19 vaccine administered in a prime-boost regimen in young and old adults (COV002): a single-blind, randomised, controlled, phase 2/3 trial. Lancet. 2020;396(10267):1979-1993. doi:10.1016/S0140-6736(20)32466-1

236. Anderson EJ, Rouphael NG, Widge AT, et al. Safety and Immunogenicity of SARS-CoV-2 mRNA-1273 Vaccine in Older Adults. N Engl J Med. 2020;383(25):2427-2438. doi:10.1056/nejmoa2028436

237. Folegatti PM, Ewer KJ, Aley PK, et al. Safety and immunogenicity of the ChAdOx1 nCoV-19 vaccine against SARS-CoV-2: a preliminary report of a phase 1/2, single-blind, randomised controlled trial. Lancet. 2020;396(10249):467-478. doi:10.1016/S0140-6736(20)31604-4

238. Walsh EE, Frenck RW, Falsey AR, et al. Safety and Immunogenicity of Two RNA-Based Covid-19 Vaccine Candidates. N Engl J Med. 2020;383(25):2439-2450. doi:10.1056/nejmoa2027906

239. Frenck RW, Klein NP, Kitchin N, et al. Safety, Immunogenicity, and Efficacy of the BNT162b2 Covid-19 Vaccine in Adolescents. N Engl J Med. 2021;385(3):239-250. doi:10.1056/nejmoa2107456

240. Zhu FC, Li YH, Guan XH, et al. Safety, tolerability, and immunogenicity of a recombinant adenovirus type-5 vectored COVID-19 vaccine: a dose-escalation, open-label, non-randomised, first-in-human trial. Lancet. 2020;395(10240):1845-1854. doi:10.1016/S0140-6736(20)31208-3

241. Zhang Y, Zeng G, Pan H, et al. Safety, tolerability, and immunogenicity of an inactivated SARS-CoV-2 vaccine in healthy adults aged 18–59 years: a randomised, double-blind, placebo-controlled, phase 1/2 clinical trial. Lancet Infect Dis. 2021;21(2):181-192. doi:10.1016/S1473-3099(20)30843-4

242. Chen P, Nirula A, Heller B, et al. SARS-CoV-2 Neutralizing Antibody LY-CoV555 in Outpatients with Covid-19. N Engl J Med. 2021;384(3):229-237. doi:10.1056/nejmoa2029849

243. Hickson LTJ, Langhi Prata LGP, Bobart SA, et al. Senolytics decrease senescent cells in humans: Preliminary report from a clinical trial of Dasatinib plus Quercetin in individuals with diabetic kidney disease. EBioMedicine. 2019;47:446-456. doi:10.1016/j.ebiom.2019.08.069

244. Justice JN, Nambiar AM, Tchkonia T, et al. Senolytics in idiopathic pulmonary fibrosis: Results from a first-in-human, open-label, pilot study. EBioMedicine. 2019;40:554-563. doi:10.1016/j.ebiom.2018.12.052

245. Bahadoer RR, Dijkstra EA, van Etten B, et al. Short-course radiotherapy followed by chemotherapy before total mesorectal excision (TME) versus preoperative chemoradiotherapy, TME, and optional adjuvant chemotherapy in locally advanced rectal cancer (RAPIDO): a randomised, open-label, phase 3 trial. Lancet Oncol. 2021;22(1):29-42. doi:10.1016/S1470-2045(20)30555-6

© 2023 Siena LM et al. *JAMA Network Open.*

246. Brown SA, Kovatchev BP, Raghinaru D, et al. Six-Month Randomized, Multicenter Trial of Closed-Loop Control in Type 1 Diabetes. N Engl J Med. 2019;381(18):1707-1717. doi:10.1056/nejmoa1907863

247. Bhatt DL, Szarek M, Pitt B, et al. Sotagliflozin in Patients with Diabetes and Chronic Kidney Disease. N Engl J Med. 2021;384(2):129-139. doi:10.1056/nejmoa2030186

248. Bhatt DL, Szarek M, Steg PG, et al. Sotagliflozin in Patients with Diabetes and Recent Worsening Heart Failure. N Engl J Med. 2021;384(2):117-128. doi:10.1056/nejmoa2030183

249. Skoulidis F, Li BT, Dy GK, et al. Sotorasib for Lung Cancers with KRAS p.G12C Mutation . N Engl J Med. 2021;384(25):2371-2381. doi:10.1056/nejmoa2103695

250. Palma DA, Olson R, Harrow S, et al. Stereotactic ablative radiotherapy for the comprehensive treatment of oligometastatic cancers: Long-term results of the SABR-COMET Phase II randomized trial. J Clin Oncol. 2020;38(25):2830-2838. doi:10.1200/JCO.20.00818

251. Palma DA, Olson R, Harrow S, et al. Stereotactic ablative radiotherapy versus standard of care palliative treatment in patients with oligometastatic cancers (SABR-COMET): a randomised, phase 2, open-label trial. Lancet. 2019;393(10185):2051-2058. doi:10.1016/S0140-6736(18)32487-5

252. Depommier C, Everard A, Druart C, et al. Supplementation with Akkermansia muciniphila in overweight and obese human volunteers: a proof-of-concept exploratory study. Nat Med. 2019;25(7):1096-1103. doi:10.1038/s41591-019-0495-2

253. Ewer KJ, Barrett JR, Belij-Rammerstorfer S, et al. T cell and antibody responses induced by a single dose of ChAdOx1 nCoV-19 (AZD1222) vaccine in a phase 1/2 clinical trial. Nat Med. 2021;27(2):270-278. doi:10.1038/s41591-020-01194-5

254. Ott PA, Bang YJ, Piha-Paul SA, et al. T-cell–inflamed gene-expression profile, programmed death ligand 1 expression, and tumor mutational burden predict efficacy in patients treated with pembrolizumab across 20 cancers: KEYNOTE-028. J Clin Oncol. 2019;37(4):318-327. doi:10.1200/JCO.2018.78.2276

255. Tabrizi SJ, Leavitt BR, Landwehrmeyer GB, et al. Targeting Huntingtin Expression in Patients with Huntington’s Disease. N Engl J Med. 2019;380(24):2307-2316. doi:10.1056/nejmoa1900907

256. Paik PK, Felip E, Veillon R, et al. Tepotinib in Non–Small-Cell Lung Cancer with MET Exon 14 Skipping Mutations . N Engl J Med. 2020;383(10):931-943. doi:10.1056/nejmoa2004407

257. Sledge GW, Toi M, Neven P, et al. The Effect of Abemaciclib Plus Fulvestrant on Overall Survival in Hormone Receptor-Positive, ERBB2-Negative Breast Cancer That Progressed on Endocrine Therapy - MONARCH 2: A Randomized Clinical Trial. JAMA Oncol. 2020;6(1):116-124. doi:10.1001/jamaoncol.2019.4782

258. Therapeutic Anticoagulation with Heparin in Critically Ill Patients with Covid-19. N Engl J Med. 2021;385(9):777-789. doi:10.1056/nejmoa2103417

259. Therapeutic Anticoagulation with Heparin in Noncritically Ill Patients with Covid-19. N Engl J Med. 2021;385(9):790-802. doi:10.1056/nejmoa2105911

260. Ma H, Campbell BCV, Parsons MW, et al. Thrombolysis Guided by Perfusion Imaging up to 9 Hours after Onset of Stroke. N Engl J Med. 2019;380(19):1795-1803. doi:10.1056/nejmoa1813046

261. Schüpke S, Neumann FJ, Menichelli M, et al. Ticagrelor or Prasugrel in Patients with Acute Coronary Syndromes. N Engl J Med. 2019;381(16):1524-1534. doi:10.1056/nejmoa1908973

© 2023 Siena LM et al. *JAMA Network Open.*

262. Mehran R, Baber U, Sharma SK, et al. Ticagrelor with or without Aspirin in High-Risk Patients after PCI. N Engl J Med. 2019;381(21):2032-2042. doi:10.1056/nejmoa1908419

263. Schuster SJ, Bishop MR, Tam CS, et al. Tisagenlecleucel in Adult Relapsed or Refractory Diffuse Large B-Cell Lymphoma. N Engl J Med. 2019;380(1):45-56. doi:10.1056/nejmoa1804980

264. Rosas IO, Bräu N, Waters M, et al. Tocilizumab in Hospitalized Patients with Severe Covid-19 Pneumonia. N Engl J Med. 2021;384(16):1503-1516. doi:10.1056/nejmoa2028700

265. Abani O, Abbas A, Abbas F, et al. Tocilizumab in patients admitted to hospital with COVID-19 (RECOVERY): a randomised, controlled, open-label, platform trial. Lancet. 2021;397(10285):1637-1645. doi:10.1016/S0140-6736(21)00676-0

266. Salama C, Han J, Yau L, et al. Tocilizumab in Patients Hospitalized with Covid-19 Pneumonia. N Engl J Med. 2021;384(1):20-30. doi:10.1056/nejmoa2030340

267. Mack MJ, Leon MB, Thourani VH, et al. Transcatheter Aortic-Valve Replacement with a Balloon-Expandable Valve in Low-Risk Patients. N Engl J Med. 2019;380(18):1695-1705. doi:10.1056/nejmoa1814052

268. Popma JJ, Deeb GM, Yakubov SJ, et al. Transcatheter Aortic-Valve Replacement with a Self-Expanding Valve in Low-Risk Patients. N Engl J Med. 2019;380(18):1706-1715. doi:10.1056/nejmoa1816885

269. Leng Z, Zhu R, Hou W, et al. Transplantation of ACE2- Mesenchymal stem cells improves the outcome of patients with covid-19 pneumonia. Aging Dis. 2020;11(2):216-228. doi:10.14336/AD.2020.0228

270. Modi S, Saura C, Yamashita T, et al. Trastuzumab Deruxtecan in Previously Treated HER2-Positive Breast Cancer. N Engl J Med. 2020;382(7):610-621. doi:10.1056/nejmoa1914510

271. Shitara K, Bang YJ, Iwasa S, et al. Trastuzumab Deruxtecan in Previously Treated HER2-Positive Gastric Cancer. N Engl J Med. 2020;382(25):2419-2430. doi:10.1056/nejmoa2004413

272. von Minckwitz G, Huang CS, Mano MS, et al. Trastuzumab Emtansine for Residual Invasive HER2-Positive Breast Cancer. N Engl J Med. 2019;380(7):617-628. doi:10.1056/nejmoa1814017

273. Matthay MA, Calfee CS, Zhuo H, et al. Treatment with allogeneic mesenchymal stromal cells for moderate to severe acute respiratory distress syndrome (START study): a randomised phase 2a safety trial. Lancet Respir Med. 2019;7(2):154-162. doi:10.1016/S2213-2600(18)30418-1

274. Morand EF, Furie R, Tanaka Y, et al. Trial of Anifrolumab in Active Systemic Lupus Erythematosus. N Engl J Med. 2020;382(3):211-221. doi:10.1056/nejmoa1912196

275. Hung IFN, Lung KC, Tso EYK, et al. Triple combination of interferon beta-1b, lopinavir–ritonavir, and ribavirin in the treatment of patients admitted to hospital with COVID-19: an open-label, randomised, phase 2 trial. Lancet. 2020;395(10238):1695-1704. doi:10.1016/S0140-6736(20)31042-4

276. Murthy RK, Loi S, Okines A, et al. Tucatinib, Trastuzumab, and Capecitabine for HER2-Positive Metastatic Breast Cancer. N Engl J Med. 2020;382(7):597-609. doi:10.1056/nejmoa1914609

277. Widmark A, Gunnlaugsson A, Beckman L, et al. Ultra-hypofractionated versus conventionally fractionated radiotherapy for prostate cancer: 5-year outcomes of the HYPO-RT-PC randomised, non-inferiority, phase 3 trial. Lancet. 2019;394(10196):385-395. doi:10.1016/S0140-6736(19)31131-6

278. Hall KD, Ayuketah A, Brychta R, et al. Ultra-Processed Diets Cause Excess Calorie Intake and Weight Gain: An Inpatient Randomized Controlled Trial of Ad Libitum Food Intake. Cell Metab. 2019;30(1):67-77.e3. doi:10.1016/j.cmet.2019.05.008

© 2023 Siena LM et al. *JAMA Network Open.*

279. Gadgeel S, Rodríguez-Abreu D, Speranza G, et al. Updated analysis from KEYNOTE-189: Pembrolizumab or placebo plus pemetrexed and platinum for previously untreated metastatic nonsquamous non–small-cell lung cancer. J Clin Oncol. 2020;38(14):1505-1517. doi:10.1200/JCO.19.03136

280. Reck M, Rodríguez-Abreu D, Robinson AG, et al. Updated analysis of KEYNOTE-024: Pembrolizumab versus platinum-based chemotherapy for advanced non–small-cell lung cancer with PD-L1 tumor proportion score of 50% or greater. J Clin Oncol. 2019;37(7):537-546. doi:10.1200/JCO.18.00149

281. Liu E, Marin D, Banerjee P, et al. Use of CAR-Transduced Natural Killer Cells in CD19-Positive Lymphoid Tumors. N Engl J Med. 2020;382(6):545-553. doi:10.1056/nejmoa1910607

282. Rouvière O, Puech P, Renard-Penna R, et al. Use of prostate systematic and targeted biopsy on the basis of multiparametric MRI in biopsy-naive patients (MRI-FIRST): a prospective, multicentre, paired diagnostic study. Lancet Oncol. 2019;20(1):100-109. doi:10.1016/S1470-2045(18)30569-2

283. Sands BE, Sandborn WJ, Panaccione R, et al. Ustekinumab as Induction and Maintenance Therapy for Ulcerative Colitis. N Engl J Med. 2019;381(13):1201-1214. doi:10.1056/nejmoa1900750

284. Coleman RL, Fleming GF, Brady MF, et al. Veliparib with First-Line Chemotherapy and as Maintenance Therapy in Ovarian Cancer. N Engl J Med. 2019;381(25):2403-2415. doi:10.1056/nejmoa1909707

285. Fischer K, Al-Sawaf O, Bahlo J, et al. Venetoclax and Obinutuzumab in Patients with CLL and Coexisting Conditions. N Engl J Med. 2019;380(23):2225-2236. doi:10.1056/nejmoa1815281

286. DiNardo CD, Pratz K, Pullarkat V, et al. Venetoclax combined with decitabine or azacitidine in treatment-naive, elderly patients with acute myeloid leukemia. Blood. 2019;133(1):7-17. doi:10.1182/blood-2018-08-868752

287. Wei AH, Strickland SA, Hou JZ, et al. Venetoclax combined with low-dose cytarabine for previously untreated patients with acute myeloid leukemia: Results from a phase Ib/II study. J Clin Oncol. 2019;37(15):1277-1284. doi:10.1200/JCO.18.01600

288. Leopoldt D. Hoffnung bei schwerer Herzinsuffizienz. Dtsch Apotheker Zeitung. 2020;160(21). doi:10.1056/NEJMoa1915928

289. Manson JE, Cook NR, Lee IM, et al. Vitamin D Supplements and Prevention of Cancer and Cardiovascular Disease. N Engl J Med. 2019;380(1):33-44. doi:10.1056/nejmoa1809944

290. Stock W, Luger SM, Advani AS, et al. A pediatric regimen for older adolescents and young adults with acute lymphoblastic leukemia: Results of CALGB 10403. Blood. 2019;133(14):1548-1559. doi:10.1182/blood-2018-10-881961

291. Ott PA, Hu-Lieskovan S, Chmielowski B, et al. A Phase Ib Trial of Personalized Neoantigen Therapy Plus Anti-PD-1 in Patients with Advanced Melanoma, Non-small Cell Lung Cancer, or Bladder Cancer. Cell. 2020;183(2):347-362.e24. doi:10.1016/j.cell.2020.08.053

292. Nunn AJ, Phillips PPJ, Meredith SK, et al. A Trial of a Shorter Regimen for Rifampin-Resistant Tuberculosis. N Engl J Med. Published online 2019. doi:10.1056/nejmoa1811867

293. Sezer A, Kilickap S, Gümüş M, et al. Cemiplimab monotherapy for first-line treatment of advanced non-small-cell lung cancer with PD-L1 of at least 50%: a multicentre, open-label, global, phase 3, randomised, controlled trial. Lancet. 2021;397(10274):592-604. doi:10.1016/S0140-6736(21)00228-2

294. Quénet F, Elias D, Roca L, et al. Cytoreductive surgery plus hyperthermic intraperitoneal chemotherapy versus cytoreductive surgery alone for colorectal peritoneal metastases (PRODIGE 7): a multicentre,

© 2023 Siena LM et al. *JAMA Network Open.*

randomised, open-label, phase 3 trial. Lancet Oncol. 2021;22(2):256-266. doi:10.1016/S1470-2045(20)30599-4

295. Mazzaferro V, El-Rayes BF, Droz dit Busset M, et al. Derazantinib (ARQ 087) in advanced or inoperable FGFR2 gene fusion-positive intrahepatic cholangiocarcinoma. Br J Cancer. 2019;120(2):165-171. doi:10.1038/s41416-018-0334-0

296. Fleshman J, Branda ME, Sargent DJ, et al. Disease-free Survival and Local Recurrence for Laparoscopic Resection Compared With Open Resection of Stage II to III Rectal Cancer: Follow-up Results of the ACOSOG Z6051 Randomized Controlled Trial. Ann Surg. 2019;269(4):589-595. doi:10.1097/SLA.0000000000003002

297. Cahn P, Madero JS, Arribas JR, et al. Dolutegravir plus lamivudine versus dolutegravir plus tenofovir disoproxil fumarate and emtricitabine in antiretroviral-naive adults with HIV-1 infection (GEMINI-1 and GEMINI-2): week 48 results from two multicentre, double-blind, randomised, non-inferiority, phase 3 trials. Lancet. 2019;393(10167):143-155. doi:10.1016/S0140-6736(18)32462-0

298. Goldberg AC, Leiter LA, Stroes ESG, et al. Effect of Bempedoic Acid vs Placebo Added to Maximally Tolerated Statins on Low-Density Lipoprotein Cholesterol in Patients at High Risk for Cardiovascular Disease: The CLEAR Wisdom Randomized Clinical Trial. JAMA - J Am Med Assoc. 2019;322(18):1780-1788. doi:10.1001/jama.2019.16585

299. Rosenzwajg M, Lorenzon R, Cacoub P, et al. Immunological and clinical effects of low-dose interleukin-2 across 11 autoimmune diseases in a single, open clinical trial. Ann Rheum Dis. 2019;78(2):209-217. doi:10.1136/annrheumdis-2018-214229

300. Macdougall IC, White C, Anker SD, et al. Intravenous Iron in Patients Undergoing Maintenance Hemodialysis. N Engl J Med. 2019;380(5):447-458. doi:10.1056/nejmoa1810742

301. Calais J, Ceci F, Eiber M, et al. 18F-fluciclovine PET-CT and 68Ga-PSMA-11 PET-CT in patients with early biochemical recurrence after prostatectomy: a prospective, single-centre, single-arm, comparative imaging trial. Lancet Oncol. 2019;20(9):1286-1294. doi:10.1016/S1470-2045(19)30415-2

302. Hofman MS, Emmett L, Sandhu S, et al. [177Lu]Lu-PSMA-617 versus cabazitaxel in patients with metastatic castration-resistant prostate cancer (TheraP): a randomised, open-label, phase 2 trial. Lancet. 2021;397(10276):797-804. doi:10.1016/S0140-6736(21)00237-3

303. Hugosson J, Roobol MJ, Månsson M, et al. A 16-yr Follow-up of the European Randomized study of Screening for Prostate Cancer(Figure presented.). Eur Urol. 2019;76(1):43-51. doi:10.1016/j.eururo.2019.02.009

304. Amarenco P, Kim JS, Labreuche J, et al. A Comparison of Two LDL Cholesterol Targets after Ischemic Stroke. N Engl J Med. 2020;382(1):9-19. doi:10.1056/nejmoa1910355

305. Dangas GD, Tijssen JGP, Wöhrle J, et al. A Controlled Trial of Rivaroxaban after Transcatheter Aortic-Valve Replacement. N Engl J Med. 2020;382(2):120-129. doi:10.1056/nejmoa1911425

306. Parletta N, Zarnowiecki D, Cho J, et al. A Mediterranean-style dietary intervention supplemented with fish oil improves diet quality and mental health in people with depression: A randomized controlled trial (HELFIMED). Nutr Neurosci. 2019;22(7):474-487. doi:10.1080/1028415X.2017.1411320

307. A Neutralizing Monoclonal Antibody for Hospitalized Patients with Covid-19. N Engl J Med. 2021;384(10):905-914. doi:10.1056/nejmoa2033130

308. Vichinsky E, Hoppe CC, Ataga KI, et al. A Phase 3 Randomized Trial of Voxelotor in Sickle Cell Disease. N Engl J Med. 2019;381(6):509-519. doi:10.1056/nejmoa1903212

© 2023 Siena LM et al. *JAMA Network Open.*

309. Harter P, Sehouli J, Lorusso D, et al. A Randomized Trial of Lymphadenectomy in Patients with Advanced Ovarian Neoplasms. N Engl J Med. 2019;380(9):822-832. doi:10.1056/nejmoa1808424

310. Paz-Ares L, Vicente D, Tafreshi A, et al. A Randomized, Placebo-Controlled Trial of Pembrolizumab Plus Chemotherapy in Patients With Metastatic Squamous NSCLC: Protocol-Specified Final Analysis of KEYNOTE-407. J Thorac Oncol. 2020;15(10):1657-1669. doi:10.1016/j.jtho.2020.06.015

311. Ying Z, Huang XF, Xiang X, et al. A safe and potent anti-CD19 CAR T cell therapy. Nat Med. 2019;25(6):947-953. doi:10.1038/s41591-019-0421-7

312. Huang AC, Orlowski RJ, Xu X, et al. A single dose of neoadjuvant PD-1 blockade predicts clinical outcomes in resectable melanoma. Nat Med. 2019;25(3):454-461. doi:10.1038/s41591-019-0357-y

313. McMurray JJV, DeMets DL, Inzucchi SE, et al. A trial to evaluate the effect of the sodium–glucose co-transporter 2 inhibitor dapagliflozin on morbidity and mortality in patients with heart failure and reduced left ventricular ejection fraction (DAPA-HF). Eur J Heart Fail. 2019;21(5):665-675. doi:10.1002/ejhf.1432

314. Johnston SRD, Harbeck N, Hegg R, et al. Abemaciclib combined with endocrine therapy for the adjuvant treatment of HR1, HER22, node-positive, high-risk, early breast cancer (monarchE). J Clin Oncol. 2020;38(34):3987-3998. doi:10.1200/JCO.20.02514

315. Fizazi K, Tran NP, Fein L, et al. Abiraterone acetate plus prednisone in patients with newly diagnosed high-risk metastatic castration-sensitive prostate cancer (LATITUDE): final overall survival analysis of a randomised, double-blind, phase 3 trial. Lancet Oncol. 2019;20(5):686-700. doi:10.1016/S1470-2045(19)30082-8

316. Sharman JP, Egyed M, Jurczak W, et al. Acalabrutinib with or without obinutuzumab versus chlorambucil and obinutuzmab for treatment-naive chronic lymphocytic leukaemia (ELEVATE TN): a randomised, controlled, phase 3 trial. Lancet. 2020;395(10232):1278-1291. doi:10.1016/S0140-6736(20)30262-2

317. Smith M, Parker C, Saad F, et al. Addition of radium-223 to abiraterone acetate and prednisone or prednisolone in patients with castration-resistant prostate cancer and bone metastases (ERA 223): a randomised, double-blind, placebo-controlled, phase 3 trial. Lancet Oncol. 2019;20(3):408-419. doi:10.1016/S1470-2045(18)30860-X

318. Felip E, Altorki N, Zhou C, et al. Adjuvant atezolizumab after adjuvant chemotherapy in resected stage IB–IIIA non-small-cell lung cancer (IMpower010): a randomised, multicentre, open-label, phase 3 trial. Lancet. 2021;398(10308):1344-1357. doi:10.1016/S0140-6736(21)02098-5

319. de Boer SM, Powell ME, Mileshkin L, et al. Adjuvant chemoradiotherapy versus radiotherapy alone in women with high-risk endometrial cancer (PORTEC-3): patterns of recurrence and post-hoc survival analysis of a randomised phase 3 trial. Lancet Oncol. 2019;20(9):1273-1285. doi:10.1016/S1470-2045(19)30395-X

320. Matei D, Filiaci V, Randall ME, et al. Adjuvant Chemotherapy plus Radiation for Locally Advanced Endometrial Cancer. N Engl J Med. 2019;380(24):2317-2326. doi:10.1056/nejmoa1813181

321. Yannopoulos D, Bartos J, Raveendran G, et al. Advanced reperfusion strategies for patients with out-of-hospital cardiac arrest and refractory ventricular fibrillation (ARREST): a phase 2, single centre, open-label, randomised controlled trial. Lancet. 2020;396(10265):1807-1816. doi:10.1016/S0140-6736(20)32338-2

322. Shaw AT, Solomon BJ, Besse B, et al. ALK resistance mutations and efficacy of lorlatinib in advanced anaplastic lymphoma kinase-positive non–small-cell lung cancer. J Clin Oncol. 2019;37(16):1370-1379. doi:10.1200/JCO.18.02236

© 2023 Siena LM et al. *JAMA Network Open.*

323. Sahin U, Oehm P, Derhovanessian E, et al. An RNA vaccine drives immunity in checkpoint-inhibitor-treated melanoma. Nature. 2020;585(7823):107-112. doi:10.1038/s41586-020-2537-9

324. Everett BM, Cornel JH, Lainscak M, et al. Anti-inflammatory therapy with canakinumab for the prevention of hospitalization for heart failure. Circulation. 2019;139(10):1289-1299. doi:10.1161/CIRCULATIONAHA.118.038010

325. Xu J, Zhang Y, Jia R, et al. Anti-PD-1 antibody SHR-1210 combined with apatinib for advanced hepatocellular carcinoma, gastric, or esophagogastric junction cancer: An Open-label, Dose Escalation and Expansion Study. Clin Cancer Res. 2019;25(2):515-523. doi:10.1158/1078-0432.CCR-18-2484

326. Hagan T, Cortese M, Rouphael N, et al. Antibiotics-Driven Gut Microbiome Perturbation Alters Immunity to Vaccines in Humans. Cell. 2019;178(6):1313-1328.e13. doi:10.1016/j.cell.2019.08.010

327. Yasuda S, Kaikita K, Akao M, et al. Antithrombotic Therapy for Atrial Fibrillation with Stable Coronary Disease. N Engl J Med. 2019;381(12):1103-1113. doi:10.1056/nejmoa1904143

328. Modi S, Park H, Murthy RK, et al. Antitumor activity and safety of trastuzumab deruxtecan in patients with HER2-low–expressing advanced breast cancer: Results from a phase Ib study. J Clin Oncol. 2020;38(17):1887-1896. doi:10.1200/JCO.19.02318

329. McBane RD, Wysokinski WE, Le-Rademacher JG, et al. Apixaban and dalteparin in active malignancy-associated venous thromboembolism: The ADAM VTE trial. J Thromb Haemost. 2020;18(2):411-421. doi:10.1111/jth.14662

330. Turk AS, Siddiqui A, Fifi JT, et al. Aspiration thrombectomy versus stent retriever thrombectomy as first-line approach for large vessel occlusion (COMPASS): a multicentre, randomised, open label, blinded outcome, non-inferiority trial. Lancet. 2019;393(10175):998-1008. doi:10.1016/S0140-6736(19)30297-1

331. Jotte R, Cappuzzo F, Vynnychenko I, et al. Atezolizumab in Combination With Carboplatin and Nab-Paclitaxel in Advanced Squamous NSCLC (IMpower131): Results From a Randomized Phase III Trial. J Thorac Oncol. 2020;15(8):1351-1360. doi:10.1016/j.jtho.2020.03.028

332. Lee MS, Ryoo BY, Hsu CH, et al. Atezolizumab with or without bevacizumab in unresectable hepatocellular carcinoma (GO30140): an open-label, multicentre, phase 1b study. Lancet Oncol. 2020;21(6):808-820. doi:10.1016/S1470-2045(20)30156-X

333. Galsky MD, Arija JÁA, Bamias A, et al. Atezolizumab with or without chemotherapy in metastatic urothelial cancer (IMvigor130): a multicentre, randomised, placebo-controlled phase 3 trial. Lancet. 2020;395(10236):1547-1557. doi:10.1016/S0140-6736(20)30230-0

334. Eng C, Kim TW, Bendell J, et al. Atezolizumab with or without cobimetinib versus regorafenib in previously treated metastatic colorectal cancer (IMblaze370): a multicentre, open-label, phase 3, randomised, controlled trial. Lancet Oncol. 2019;20(6):849-861. doi:10.1016/S1470-2045(19)30027-0

335. Gutzmer R, Stroyakovskiy D, Gogas H, et al. Atezolizumab, vemurafenib, and cobimetinib as first-line treatment for unresectable advanced BRAFV600 mutation-positive melanoma (IMspire150): primary analysis of the randomised, double-blind, placebo-controlled, phase 3 trial. Lancet. 2020;395(10240):1835-1844. doi:10.1016/S0140-6736(20)30934-X

336. Heerspink HJL, Parving HH, Andress DL, et al. Atrasentan and renal events in patients with type 2 diabetes and chronic kidney disease (SONAR): a double-blind, randomised, placebo-controlled trial. Lancet. 2019;393(10184):1937-1947. doi:10.1016/S0140-6736(19)30772-X

© 2023 Siena LM et al. *JAMA Network Open.*

337. Furtado RHM, Berwanger O, Fonseca HA, et al. Azithromycin in addition to standard of care versus standard of care alone in the treatment of patients admitted to the hospital with severe COVID-19 in Brazil (COALITION II): a randomised clinical trial. Lancet. 2020;396(10256):959-967. doi:10.1016/S0140-6736(20)31862-6

338. Dougan M, Nirula A, Azizad M, et al. Bamlanivimab plus Etesevimab in Mild or Moderate Covid-19. N Engl J Med. 2021;385(15):1382-1392. doi:10.1056/nejmoa2102685

339. Guttman-Yassky E, Silverberg JI, Nemoto O, et al. Baricitinib in adult patients with moderate-to-severe atopic dermatitis: A phase 2 parallel, double-blinded, randomized placebo-controlled multiple-dose study. J Am Acad Dermatol. 2019;80(4):913-921.e9. doi:10.1016/j.jaad.2018.01.018

340. Taggart DP, Benedetto U, Gerry S, et al. Bilateral versus Single Internal-Thoracic-Artery Grafts at 10 Years. N Engl J Med. 2019;380(5):437-446. doi:10.1056/nejmoa1808783

341. Qin S, Ren Z, Meng Z, et al. Camrelizumab in patients with previously treated advanced hepatocellular carcinoma: a multicentre, open-label, parallel-group, randomised, phase 2 trial. Lancet Oncol. 2020;21(4):571-580. doi:10.1016/S1470-2045(20)30011-5

342. Huang J, Xu J, Chen Y, et al. Camrelizumab versus investigator's choice of chemotherapy as second-line therapy for advanced or metastatic oesophageal squamous cell carcinoma (ESCORT): a multicentre, randomised, open-label, phase 3 study. Lancet Oncol. 2020;21(6):832-842. doi:10.1016/S1470-2045(20)30110-8

343. Teerlink JR, Diaz R, Felker GM, et al. Cardiac Myosin Activation with Omecamtiv Mecarbil in Systolic Heart Failure. N Engl J Med. 2021;384(2):105-116. doi:10.1056/nejmoa2025797

344. Pitt B, Filippatos G, Agarwal R, et al. Cardiovascular Events with Finerenone in Kidney Disease and Type 2 Diabetes. N Engl J Med. 2021;385(24):2252-2263. doi:10.1056/nejmoa2110956

345. Berdeja JG, Madduri D, Usmani SZ, et al. Ciltacabtagene autoleucel, a B-cell maturation antigen-directed chimeric antigen receptor T-cell therapy in patients with relapsed or refractory multiple myeloma (CARTITUDE-1): a phase 1b/2 open-label study. Lancet. 2021;398(10297):314-324. doi:10.1016/S0140-6736(21)00933-8

346. Powles T, Kockx M, Rodriguez-Vida A, et al. Clinical efficacy and biomarker analysis of neoadjuvant atezolizumab in operable urothelial carcinoma in the ABACUS trial. Nat Med. 2019;25(11):1706-1714. doi:10.1038/s41591-019-0628-7

347. Massarelli E, William W, Johnson F, et al. Combining Immune Checkpoint Blockade and Tumor-Specific Vaccine for Patients with Incurable Human Papillomavirus 16-Related Cancer: A Phase 2 Clinical Trial. JAMA Oncol. 2019;5(1):67-73. doi:10.1001/jamaoncol.2018.4051

348. Bex A, Mulders P, Jewett M, et al. Comparison of Immediate vs Deferred Cytoreductive Nephrectomy in Patients with Synchronous Metastatic Renal Cell Carcinoma Receiving Sunitinib: The SURTIME Randomized Clinical Trial. JAMA Oncol. 2019;5(2):164-170. doi:10.1001/jamaoncol.2018.5543

349. Suzuki K, Saji H, Aokage K, et al. Comparison of pulmonary segmentectomy and lobectomy: Safety results of a randomized trial. J Thorac Cardiovasc Surg. 2019;158(3):895-907. doi:10.1016/j.jtcvs.2019.03.090

350. Conservative Oxygen Therapy during Mechanical Ventilation in the ICU. N Engl J Med. 2020;382(11):989-998. doi:10.1056/nejmoa1903297

© 2023 Siena LM et al. *JAMA Network Open.*

351. Beasley R, Holliday M, Reddel HK, et al. Controlled Trial of Budesonide–Formoterol as Needed for Mild Asthma. N Engl J Med. 2019;380(21):2020-2030. doi:10.1056/nejmoa1901963

352. Abani O, Abbas A, Abbas F, et al. Convalescent plasma in patients admitted to hospital with COVID-19 (RECOVERY): a randomised controlled, open-label, platform trial. Lancet. 2021;397(10289):2049-2059. doi:10.1016/S0140-6736(21)00897-7

353. Lemkes JS, Janssens GN, van der Hoeven NW, et al. Coronary Angiography after Cardiac Arrest without ST-Segment Elevation. N Engl J Med. 2019;380(15):1397-1407. doi:10.1056/nejmoa1816897

354. Shaw AT, Riely GJ, Bang YJ, et al. Crizotinib in ROS1-rearranged advanced non-small-cell lung cancer (NSCLC): updated results, including overall survival, from PROFILE 1001. Ann Oncol. 2019;30(7):1121-1126. doi:10.1093/annonc/mdz131

355. Levine A, Wine E, Assa A, et al. Crohn's Disease Exclusion Diet Plus Partial Enteral Nutrition Induces Sustained Remission in a Randomized Controlled Trial. Gastroenterology. 2019;157(2):440-450.e8. doi:10.1053/j.gastro.2019.04.021

356. Andrade JG, Wells GA, Deyell MW, et al. Cryoablation or Drug Therapy for Initial Treatment of Atrial Fibrillation. N Engl J Med. 2021;384(4):305-315. doi:10.1056/nejmoa2029980

357. Andrade JG, Champagne J, Dubuc M, et al. Cryoballoon or Radiofrequency Ablation for Atrial Fibrillation Assessed by Continuous Monitoring: A Randomized Clinical Trial. Circulation. 2019;140(22):1779-1788. doi:10.1161/CIRCULATIONAHA.119.042622

358. Johnson AJ, Vangay P, Al-Ghalith GA, et al. Daily Sampling Reveals Personalized Diet-Microbiome Associations in Humans. Cell Host Microbe. 2019;25(6):789-802.e5. doi:10.1016/j.chom.2019.05.005

359. Nassif ME, Windsor S, Tang F, et al. Dapagliflozin effects on biomarkers, symptoms, and functional status in patients with heart failure with reduced ejection fraction. Circulation. 2019;140(18). doi:10.1161/CIRCULATIONAHA.119.042929

360. Voorhees PM, Kaufman JL, Laubach J, et al. Daratumumab, lenalidomide, bortezomib, and dexamethasone for transplant-eligible newly diagnosed multiple myeloma: The GRIFFIN trial. Blood. 2020;136(8):936-945. doi:10.1182/blood.2020005288

361. Suo T, Liu X, Feng J, et al. ddPCR: a more accurate tool for SARS-CoV-2 detection in low viral load specimens. Emerg Microbes Infect. 2020;9(1):1259-1268. doi:10.1080/22221751.2020.1772678

362. Venter WDF, Moorhouse M, Sokhela S, et al. Dolutegravir plus Two Different Prodrugs of Tenofovir to Treat HIV. N Engl J Med. 2019;381(9):803-815. doi:10.1056/nejmoa1902824

363. Mintun MA, Lo AC, Duggan Evans C, et al. Donanemab in Early Alzheimer's Disease. N Engl J Med. 2021;384(18):1691-1704. doi:10.1056/NEJMoa2100708

364. Gerstein HC, Colhoun HM, Dagenais GR, et al. Dulaglutide and renal outcomes in type 2 diabetes: an exploratory analysis of the REWIND randomised, placebo-controlled trial. Lancet. 2019;394(10193):131-138. doi:10.1016/S0140-6736(19)31150-X

365. Guttman-Yassky E, Bissonnette R, Ungar B, et al. Dupilumab progressively improves systemic and cutaneous abnormalities in patients with atopic dermatitis. J Allergy Clin Immunol. 2019;143(1):155-172. doi:10.1016/j.jaci.2018.08.022

366. Lean MEJ, Leslie WS, Barnes AC, et al. Durability of a primary care-led weight-management intervention for remission of type 2 diabetes: 2-year results of the DiRECT open-label, cluster-randomised trial. Lancet Diabetes Endocrinol. 2019;7(5):344-355. doi:10.1016/S2213-8587(19)30068-3

© 2023 Siena LM et al. *JAMA Network Open.*

367. Søndergaard L, Ihlemann N, Capodanno D, et al. Durability of Transcatheter and Surgical Bioprosthetic Aortic Valves in Patients at Lower Surgical Risk. J Am Coll Cardiol. 2019;73(5):546-553. doi:10.1016/j.jacc.2018.10.083

368. Nghiem P, Bhatia S, Lipson EJ, et al. Durable tumor regression and overall survival in patients with advanced Merkel cell carcinoma receiving pembrolizumab as first-line therapy. J Clin Oncol. 2019;37(9):693-702. doi:10.1200/JCO.18.01896

369. O'Reilly EM, Oh DY, Dhani N, et al. Durvalumab with or Without Tremelimumab for Patients with Metastatic Pancreatic Ductal Adenocarcinoma: A Phase 2 Randomized Clinical Trial. JAMA Oncol. 2019;5(10):1431-1438. doi:10.1001/jamaoncol.2019.1588

370. Rizvi NA, Cho BC, Reinmuth N, et al. Durvalumab with or Without Tremelimumab vs Standard Chemotherapy in First-line Treatment of Metastatic Non-Small Cell Lung Cancer: The MYSTIC Phase 3 Randomized Clinical Trial. JAMA Oncol. 2020;6(5):661-674. doi:10.1001/jamaoncol.2020.0237

371. Gottlieb RL, Vaca CE, Paredes R, et al. Early Remdesivir to Prevent Progression to Severe Covid-19 in Outpatients. N Engl J Med. 2022;386(4):305-315. doi:10.1056/nejmoa2116846

372. Shehabi Y, Howe BD, Bellomo R, et al. Early Sedation with Dexmedetomidine in Critically Ill Patients. N Engl J Med. 2019;380(26):2506-2517. doi:10.1056/nejmoa1904710

373. Fadel R, Morrison AR, Vahia A, et al. Early Short-Course Corticosteroids in Hospitalized Patients with COVID-19. Clin Infect Dis. 2020;71(16):2114-2120. doi:10.1093/cid/ciaa601

374. Kang DH, Park SJ, Lee SA, et al. Early Surgery or Conservative Care for Asymptomatic Aortic Stenosis. N Engl J Med. 2020;382(2):111-119. doi:10.1056/nejmoa1912846

375. Jamshed H, Beyl RA, Manna DLD, Yang ES, Ravussin E, Peterson CM. Early time-restricted feeding improves 24-hour glucose levels and affects markers of the circadian clock, aging, and autophagy in humans. Nutrients. 2019;11(6). doi:10.3390/nu11061234

376. Murai IH, Fernandes AL, Sales LP, et al. Effect of a Single High Dose of Vitamin D3on Hospital Length of Stay in Patients with Moderate to Severe COVID-19: A Randomized Clinical Trial. JAMA - J Am Med Assoc. 2021;325(11):1053-1060. doi:10.1001/jama.2020.26848

377. Mark DB, Anstrom KJ, Sheng S, et al. Effect of Catheter Ablation vs Medical Therapy on Quality of Life among Patients with Atrial Fibrillation: The CABANA Randomized Clinical Trial. JAMA - J Am Med Assoc. 2019;321(13):1275-1285. doi:10.1001/jama.2019.0692

378. Deftereos SG, Giannopoulos G, Vrachatis DA, et al. Effect of Colchicine vs Standard Care on Cardiac and Inflammatory Biomarkers and Clinical Outcomes in Patients Hospitalized with Coronavirus Disease 2019: The GRECCO-19 Randomized Clinical Trial. JAMA Netw Open. 2020;3(6). doi:10.1001/jamanetworkopen.2020.13136

379. Petrie MC, Verma S, Docherty KF, et al. Effect of Dapagliflozin on Worsening Heart Failure and Cardiovascular Death in Patients with Heart Failure with and Without Diabetes. JAMA - J Am Med Assoc. 2020;323(14):1353-1368. doi:10.1001/jama.2020.1906

380. Wildes TS, Mickle AM, Abdallah A Ben, et al. Effect of electroencephalography-guided anesthetic administration on postoperative delirium among older adults undergoing major surgery the engages randomized clinical trial. JAMA - J Am Med Assoc. 2019;321(5):473-483. doi:10.1001/jama.2018.22005

© 2023 Siena LM et al. *JAMA Network Open.*

381. Verma S, Mazer CD, Yan AT, et al. Effect of Empagliflozin on Left Ventricular Mass in Patients with Type 2 Diabetes Mellitus and Coronary Artery Disease: The EMPA-HEART CardioLink-6 Randomized Clinical Trial. Circulation. 2019;140(21):1693-1702. doi:10.1161/CIRCULATIONAHA.119.042375

382. Nicholls SJ, Lincoff AM, Garcia M, et al. Effect of High-Dose Omega-3 Fatty Acids vs Corn Oil on Major Adverse Cardiovascular Events in Patients at High Cardiovascular Risk: The STRENGTH Randomized Clinical Trial. JAMA - J Am Med Assoc. 2020;324(22):2268-2280. doi:10.1001/jama.2020.22258

383. Dequin PF, Heming N, Meziani F, et al. Effect of Hydrocortisone on 21-Day Mortality or Respiratory Support among Critically Ill Patients with COVID-19: A Randomized Clinical Trial. JAMA - J Am Med Assoc. 2020;324(13):1298-1306. doi:10.1001/jama.2020.16761

384. Self WH, Semler MW, Leither LM, et al. Effect of Hydroxychloroquine on Clinical Status at 14 Days in Hospitalized Patients with COVID-19: A Randomized Clinical Trial. JAMA - J Am Med Assoc. 2020;324(21):2165-2176. doi:10.1001/jama.2020.22240

385. Kim HH, Han SU, Kim MC, et al. Effect of Laparoscopic Distal Gastrectomy vs Open Distal Gastrectomy on Long-term Survival Among Patients With Stage I Gastric Cancer: The KLASS-01 Randomized Clinical Trial. JAMA Oncol. 2019;5(4):506-513. doi:10.1001/jamaoncol.2018.6727

386. Rosenstock J, Kahn SE, Johansen OE, et al. Effect of Linagliptin vs Glimepiride on Major Adverse Cardiovascular Outcomes in Patients with Type 2 Diabetes: The CAROLINA Randomized Clinical Trial. JAMA - J Am Med Assoc. 2019;322(12):1155-1166. doi:10.1001/jama.2019.13772

387. Hahn JY, Song Y Bin, Oh JH, et al. Effect of P2Y12 Inhibitor Monotherapy vs Dual Antiplatelet Therapy on Cardiovascular Events in Patients Undergoing Percutaneous Coronary Intervention: The SMART-CHOICE Randomized Clinical Trial. JAMA - J Am Med Assoc. 2019;321(24):2428-2437. doi:10.1001/jama.2019.8146

388. Nanda R, Liu MC, Yau C, et al. Effect of Pembrolizumab Plus Neoadjuvant Chemotherapy on Pathologic Complete Response in Women with Early-Stage Breast Cancer: An Analysis of the Ongoing Phase 2 Adaptively Randomized I-SPY2 Trial. JAMA Oncol. 2020;6(5):676-684. doi:10.1001/jamaoncol.2019.6650

389. Kim BK, Hong SJ, Cho YH, et al. Effect of Ticagrelor Monotherapy vs Ticagrelor with Aspirin on Major Bleeding and Cardiovascular Events in Patients with Acute Coronary Syndrome: The TICO Randomized Clinical Trial. JAMA - J Am Med Assoc. 2020;323(23):2407-2416. doi:10.1001/jama.2020.7580

390. Veiga VC, Prats JAGG, Farias DLC, et al. Effect of tocilizumab on clinical outcomes at 15 days in patients with severe or critical coronavirus disease 2019: Randomised controlled trial. BMJ. 2021;372. doi:10.1136/bmj.n84

391. Fujii T, Luethi N, Young PJ, et al. Effect of Vitamin C, Hydrocortisone, and Thiamine vs Hydrocortisone Alone on Time Alive and Free of Vasopressor Support among Patients with Septic Shock: The VITAMINS Randomized Clinical Trial. JAMA - J Am Med Assoc. 2020;323(5):423-431. doi:10.1001/jama.2019.22176

392. Boevé LMS, Hulshof MCCM, Vis AN, et al. Effect on Survival of Androgen Deprivation Therapy Alone Compared to Androgen Deprivation Therapy Combined with Concurrent Radiation Therapy to the Prostate in Patients with Primary Bone Metastatic Prostate Cancer in a Prospective Randomised Clinical Trial: Data from the HORRAD Trial. Eur Urol. 2019;75(3):410-418. doi:10.1016/j.eururo.2018.09.008

393. Ramadurai N, Gurunathan D, Samuel AV, Subramanian E, Rodrigues SJL. Effectiveness of 2% Articaine as an anesthetic agent in children: randomized controlled trial. Clin Oral Investig. 2019;23(9):3543-3550. doi:10.1007/s00784-018-2775-5

© 2023 Siena LM et al. *JAMA Network Open.*

394. Wan Y, Wang F, Yuan J, et al. Effects of dietary fat on gut microbiota and faecal metabolites, and their relationship with cardiometabolic risk factors: a 6-month randomised controlled-feeding trial. Gut. 2019;68(8):1417-1429. doi:10.1136/gutjnl-2018-317609

395. Gehrig JL, Venkatesh S, Chang HW, et al. Effects of microbiota-directed foods in gnotobiotic animals and undernourished children. Science (80-). 2019;365(6449). doi:10.1126/science.aau4732

396. Solomon SD, Adams D, Kristen A, et al. Effects of Patisiran, an RNA Interference Therapeutic, on Cardiac Parameters in Patients with Hereditary Transthyretin-Mediated Amyloidosis: Analysis of the APOLLO Study. Circulation. 2019;139(4):431-443. doi:10.1161/CIRCULATIONAHA.118.035831

397. Davis AK, Barrett FS, May DG, et al. Effects of Psilocybin-Assisted Therapy on Major Depressive Disorder: A Randomized Clinical Trial. JAMA Psychiatry. 2021;78(5):481-489. doi:10.1001/jamapsychiatry.2020.3285

398. Disis ML, Taylor MH, Kelly K, et al. Efficacy and Safety of Avelumab for Patients with Recurrent or Refractory Ovarian Cancer: Phase 1b Results from the JAVELIN Solid Tumor Trial. JAMA Oncol. 2019;5(3):393-401. doi:10.1001/jamaoncol.2018.6258

399. Marconi VC, Ramanan A V., de Bono S, et al. Efficacy and safety of baricitinib for the treatment of hospitalised adults with COVID-19 (COV-BARRIER): a randomised, double-blind, parallel-group, placebo-controlled phase 3 trial. Lancet Respir Med. 2021;9(12):1407-1418. doi:10.1016/S2213-2600(21)00331-3

400. Bassetti M, Echols R, Matsunaga Y, et al. Efficacy and safety of cefiderocol or best available therapy for the treatment of serious infections caused by carbapenem-resistant Gram-negative bacteria (CREDIBLE-CR): a randomised, open-label, multicentre, pathogen-focused, descriptive, phase 3 trial. Lancet Infect Dis. 2021;21(2):226-240. doi:10.1016/S1473-3099(20)30796-9

401. Simpson EL, Paller AS, Siegfried EC, et al. Efficacy and Safety of Dupilumab in Adolescents with Uncontrolled Moderate to Severe Atopic Dermatitis: A Phase 3 Randomized Clinical Trial. JAMA Dermatology. 2020;156(1):44-56. doi:10.1001/jamadermatol.2019.3336

402. Popova V, Daly EJ, Trivedi M, et al. Efficacy and safety of flexibly dosed esketamine nasal spray combined with a newly initiated oral antidepressant in treatment-resistant depression: A randomized double-blind active-controlled study. Am J Psychiatry. 2019;176(6):428-438. doi:10.1176/appi.ajp.2019.19020172

403. Hu K, Guan W jie, Bi Y, et al. Efficacy and safety of Lianhuaqingwen capsules, a repurposed Chinese herb, in patients with coronavirus disease 2019: A multicenter, prospective, randomized controlled trial. Phytomedicine. 2021;85. doi:10.1016/j.phymed.2020.153242

404. Hill MD, Goyal M, Menon BK, et al. Efficacy and safety of nerinetide for the treatment of acute ischaemic stroke (ESCAPE-NA1): a multicentre, double-blind, randomised controlled trial. Lancet. 2020;395(10227):878-887. doi:10.1016/S0140-6736(20)30258-0

405. Shah MA, Kojima T, Hochhauser D, et al. Efficacy and Safety of Pembrolizumab for Heavily Pretreated Patients with Advanced, Metastatic Adenocarcinoma or Squamous Cell Carcinoma of the Esophagus: The Phase 2 KEYNOTE-180 Study. JAMA Oncol. 2019;5(4):546-550. doi:10.1001/jamaoncol.2018.5441

406. Shitara K, Van Cutsem E, Bang YJ, et al. Efficacy and Safety of Pembrolizumab or Pembrolizumab plus Chemotherapy vs Chemotherapy Alone for Patients with First-line, Advanced Gastric Cancer: The KEYNOTE-062 Phase 3 Randomized Clinical Trial. JAMA Oncol. 2020;6(10):1571-1580. doi:10.1001/jamaoncol.2020.3370

© 2023 Siena LM et al. *JAMA Network Open.*

407. Böhm M, Kario K, Kandzari DE, et al. Efficacy of catheter-based renal denervation in the absence of antihypertensive medications (SPYRAL HTN-OFF MED Pivotal): a multicentre, randomised, sham-controlled trial. Lancet. 2020;395(10234):1444-1451. doi:10.1016/S0140-6736(20)30554-7

408. Emary KRW, Golubchik T, Aley PK, et al. Efficacy of ChAdOx1 nCoV-19 (AZD1222) vaccine against SARS-CoV-2 variant of concern 202012/01 (B.1.1.7): an exploratory analysis of a randomised controlled trial. Lancet. 2021;397(10282):1351-1362. doi:10.1016/S0140-6736(21)00628-0

409. Daly EJ, Trivedi MH, Janik A, et al. Efficacy of Esketamine Nasal Spray Plus Oral Antidepressant Treatment for Relapse Prevention in Patients with Treatment-Resistant Depression: A Randomized Clinical Trial. JAMA Psychiatry. 2019;76(9):893-903. doi:10.1001/jamapsychiatry.2019.1189

410. Shinde V, Bhikha S, Hoosain Z, et al. Efficacy of NVX-CoV2373 Covid-19 Vaccine against the B.1.351 Variant. N Engl J Med. 2021;384(20):1899-1909. doi:10.1056/nejmoa2103055

411. Drilon A, Oxnard GR, Tan DSW, et al. Efficacy of Selpercatinib in RET Fusion–Positive Non–Small-Cell Lung Cancer . N Engl J Med. 2020;383(9):813-824. doi:10.1056/nejmoa2005653

412. Wirth LJ, Sherman E, Robinson B, et al. Efficacy of Selpercatinib in RET -Altered Thyroid Cancers . N Engl J Med. 2020;383(9):825-835. doi:10.1056/nejmoa2005651

413. Daver N, Garcia-Manero G, Basu S, et al. Efficacy, safety, and biomarkers of response to azacitidine and nivolumab in relapsed/ refractory acute myeloid leukemia: A nonrandomized, open-label, phase II study. Cancer Discov. 2019;9(3):370-383. doi:10.1158/2159-8290.CD-18-0774

414. Werner YB, Hakanson B, Martinek J, et al. Endoscopic or Surgical Myotomy in Patients with Idiopathic Achalasia. N Engl J Med. 2019;381(23):2219-2229. doi:10.1056/nejmoa1905380

415. Yang P, Zhang Y, Zhang L, et al. Endovascular Thrombectomy with or without Intravenous Alteplase in Acute Stroke. N Engl J Med. 2020;382(21):1981-1993. doi:10.1056/nejmoa2001123

416. Liu X, Dai Q, Ye R, et al. Endovascular treatment versus standard medical treatment for vertebrobasilar artery occlusion (BEST): an open-label, randomised controlled trial. Lancet Neurol. 2020;19(2):115-122. doi:10.1016/S1474-4422(19)30395-3

417. Powles T, Rosenberg JE, Sonpavde GP, et al. Enfortumab Vedotin in Previously Treated Advanced Urothelial Carcinoma. N Engl J Med. 2021;384(12):1125-1135. doi:10.1056/nejmoa2035807

418. Ghorashian S, Kramer AM, Onuoha S, et al. Enhanced CAR T cell expansion and prolonged persistence in pediatric patients with ALL treated with a low-affinity CD19 CAR. Nat Med. 2019;25(9):1408-1414. doi:10.1038/s41591-019-0549-5

419. Raal FJ, Rosenson RS, Reeskamp LF, et al. Evinacumab for Homozygous Familial Hypercholesterolemia. *N Engl J Med*. 2020;383(8):711-720. doi:10.1056/NEJMoa2004215

420. Xu J, Chen LJ, Yang SS, et al. Exploratory trial of a biepitopic CAR T-targeting B cell maturation antigen in relapsed/refractory multiple myeloma. Proc Natl Acad Sci U S A. 2019;116(19):9543-9551. doi:10.1073/pnas.1819745116

421. Ponikowski P, Kirwan BA, Anker SD, et al. Ferric carboxymaltose for iron deficiency at discharge after acute heart failure: a multicentre, double-blind, randomised, controlled trial. Lancet. 2020;396(10266):1895-1904. doi:10.1016/S0140-6736(20)32339-4

422. Newsome PN, Sasso M, Deeks JJ, et al. FibroScan-AST (FAST) score for the non-invasive identification of patients with non-alcoholic steatohepatitis with significant activity and fibrosis: a prospective derivation

and global validation study. Lancet Gastroenterol Hepatol. 2020;5(4):362-373. doi:10.1016/S2468-1253(19)30383-8

423. Munir T, Brown JR, O'Brien S, et al. Final analysis from RESONATE: Up to six years of follow-up on ibrutinib in patients with previously treated chronic lymphocytic leukemia or small lymphocytic lymphoma. Am J Hematol. 2019;94(12):1353-1363. doi:10.1002/ajh.25638

424. Abrahao A, Meng Y, Llinas M, et al. First-in-human trial of blood–brain barrier opening in amyotrophic lateral sclerosis using MR-guided focused ultrasound. Nat Commun. 2019;10(1). doi:10.1038/s41467-019-12426-9

425. Sikic BI, Lakhani N, Patnaik A, et al. First-in-human, first-in-class phase i trial of the anti-CD47 antibody Hu5F9-G4 in patients with advanced cancers. J Clin Oncol. 2019;37(12):946-953. doi:10.1200/JCO.18.02018

426. Shaw AT, Bauer TM, de Marinis F, et al. First-Line Lorlatinib or Crizotinib in Advanced ALK -Positive Lung Cancer . N Engl J Med. 2020;383(21):2018-2029. doi:10.1056/nejmoa2027187

427. Ready N, Hellmann MD, Awad MM, et al. First-line nivolumab plus ipilimumab in advanced non-small-cell lung cancer (CheckMate 568): Outcomes by programmed death ligand 1 and tumor mutational burden as biomarkers. J Clin Oncol. 2019;37(12):992-1000. doi:10.1200/JCO.18.01042

428. Makkar RR, Thourani VH, Mack MJ, et al. Five-Year Outcomes of Transcatheter or Surgical Aortic-Valve Replacement. N Engl J Med. 2020;382(9):799-809. doi:10.1056/nejmoa1910555

429. Topalian SL, Hodi FS, Brahmer JR, et al. Five-Year Survival and Correlates among Patients with Advanced Melanoma, Renal Cell Carcinoma, or Non-Small Cell Lung Cancer Treated with Nivolumab. JAMA Oncol. 2019;5(10):1411-1420. doi:10.1001/jamaoncol.2019.2187

430. Lenze EJ, Mattar C, Zorumski CF, et al. Fluvoxamine vs Placebo and Clinical Deterioration in Outpatients with Symptomatic COVID-19: A Randomized Clinical Trial. JAMA - J Am Med Assoc. 2020;324(22):2292-2300. doi:10.1001/jama.2020.22760

431. Wei D, Liu JY, Sun Y, et al. Frozen versus fresh single blastocyst transfer in ovulatory women: a multicentre, randomised controlled trial. Lancet. 2019;393(10178):1310-1318. doi:10.1016/S0140-6736(18)32843-5

432. Hosomi Y, Morita S, Sugawara S, et al. Gefitinib alone versus gefitinib plus chemotherapy for non–small-cell lung cancer with mutated epidermal growth factor receptor: NEJ009 study. J Clin Oncol. 2020;38(2):115-123. doi:10.1200/JCO.19.01488

433. Noronha V, Patil VM, Joshi A, et al. Gefitinib versus gefitinib plus pemetrexed and carboplatin chemotherapy in EGFR-mutated lung cancer. J Clin Oncol. 2020;38(2):124-136. doi:10.1200/JCO.19.01154

434. Edeline J, Benabdelghani M, Bertaut A, et al. Gemcitabine and oxaliplatin chemotherapy or surveillance in resected biliary tract cancer (Prodige 12-accord 18-Unicancer GI): A randomized phase III study. J Clin Oncol. 2019;37(8):658-667. doi:10.1200/JCO.18.00050

435. Shroff RT, Javle MM, Xiao L, et al. Gemcitabine, Cisplatin, and nab-Paclitaxel for the Treatment of Advanced Biliary Tract Cancers: A Phase 2 Clinical Trial. JAMA Oncol. 2019;5(6):824-830. doi:10.1001/jamaoncol.2019.0270

436. Rodon J, Soria JC, Berger R, et al. Genomic and transcriptomic profiling expands precision cancer medicine: the WINTHER trial. Nat Med. 2019;25(5):751-758. doi:10.1038/s41591-019-0424-4

437. Spertus JA, Jones PG, Maron DJ, et al. Health-Status Outcomes with Invasive or Conservative Care in Coronary Disease. N Engl J Med. 2020;382(15):1408-1419. doi:10.1056/nejmoa1916370

© 2023 Siena LM et al. *JAMA Network Open.*

438. Woolley AE, Singh SK, Goldberg HJ, et al. Heart and Lung Transplants from HCV-Infected Donors to Uninfected Recipients. N Engl J Med. 2019;380(17):1606-1617. doi:10.1056/nejmoa1812406

439. Brown PD, Gondi V, Pugh S, et al. Hippocampal avoidance during whole-brain radiotherapy plus memantine for patients with brain metastases: Phase III trial NRG oncology CC001. J Clin Oncol. 2020;38(10):1019-1029. doi:10.1200/JCO.19.02767

440. Berry SE, Valdes AM, Drew DA, et al. Human postprandial responses to food and potential for precision nutrition. Nat Med. 2020;26(6):964-973. doi:10.1038/s41591-020-0934-0

441. Skipper CP, Pastick KA, Engen NW, et al. Hydroxychloroquine in nonhospitalized adults with early covid-19: A randomized trial. Ann Intern Med. 2020;173(8):623-631. doi:10.7326/M20-4207

442. Dankiewicz J, Cronberg T, Lilja G, et al. Hypothermia versus Normothermia after Out-of-Hospital Cardiac Arrest. N Engl J Med. 2021;384(24):2283-2294. doi:10.1056/nejmoa2100591

443. Rozeman EA, Menzies AM, van Akkooi ACJ, et al. Identification of the optimal combination dosing schedule of neoadjuvant ipilimumab plus nivolumab in macroscopic stage III melanoma (OpACIN-neo): a multicentre, phase 2, randomised, controlled trial. Lancet Oncol. 2019;20(7):948-960. doi:10.1016/S1470-2045(19)30151-2

444. Gilbert PB, Montefiori DC, McDermott AB, et al. Immune correlates analysis of the mRNA-1273 COVID-19 vaccine efficacy clinical trial. Science (80-). 2022;375(6576):43-50. doi:10.1126/science.abm3425

445. Borobia AM, Carcas AJ, Pérez-Olmeda M, et al. Immunogenicity and reactogenicity of BNT162b2 booster in ChAdOx1-S-primed participants (CombiVacS): a multicentre, open-label, randomised, controlled, phase 2 trial. Lancet. 2021;398(10295):121-130. doi:10.1016/S0140-6736(21)01420-3

446. Wang Z, Bergeron N, Levison BS, et al. Impact of chronic dietary red meat, white meat, or non-meat protein on trimethylamine N-oxide metabolism and renal excretion in healthy men and women. Eur Heart J. 2019;40(7):583-594. doi:10.1093/eurheartj/ehy799

447. Basir MB, Kapur NK, Patel K, et al. Improved Outcomes Associated with the use of Shock Protocols: Updates from the National Cardiogenic Shock Initiative. Catheter Cardiovasc Interv. 2019;93(7):1173-1183. doi:10.1002/ccd.28307

448. Raal FJ, Kallend D, Ray KK, et al. Inclisiran for the Treatment of Heterozygous Familial Hypercholesterolemia. N Engl J Med. 2020;382(16):1520-1530. doi:10.1056/nejmoa1913805

449. Humphrey JH, Mbuya MNN, Ntozini R, et al. Independent and combined effects of improved water, sanitation, and hygiene, and improved complementary feeding, on child stunting and anaemia in rural Zimbabwe: a cluster-randomised trial. Lancet Glob Heal. 2019;7(1):e132-e147. doi:10.1016/S2214-109X(18)30374-7

450. Cree BAC, Bennett JL, Kim HJ, et al. Inebilizumab for the treatment of neuromyelitis optica spectrum disorder (N-MOmentum): a double-blind, randomised placebo-controlled phase 2/3 trial. Lancet. 2019;394(10206):1352-1363. doi:10.1016/S0140-6736(19)31817-3

451. Ramakrishnan S, Nicolau D V., Langford B, et al. Inhaled budesonide in the treatment of early COVID-19 (STOIC): a phase 2, open-label, randomised controlled trial. Lancet Respir Med. 2021;9(7):763-772. doi:10.1016/S2213-2600(21)00160-0

452. Brand DH, Tree AC, Ostler P, et al. Intensity-modulated fractionated radiotherapy versus stereotactic body radiotherapy for prostate cancer (PACE-B): acute toxicity findings from an international, randomised,

© 2023 Siena LM et al. *JAMA Network Open.*

open-label, phase 3, non-inferiority trial. Lancet Oncol. 2019;20(11):1531-1543. doi:10.1016/S1470-2045(19)30569-8

453. Zhou Q, Chen V, Shannon CP, et al. Interferon-α2b Treatment for COVID-19. Front Immunol. 2020;11. doi:10.3389/fimmu.2020.01061

454. Lin NU, Borges V, Anders C, et al. Intracranial efficacy and survival with tucatinib plus trastuzumab and capecitabine for previously treated HER2-positive breast cancer with brain metastases in the HER2CLIMB trial. J Clin Oncol. 2020;38(23):2610-2619. doi:10.1200/JCO.20.00775

455. van Hilst J, De Rooij T, Bosscha K, et al. Laparoscopic versus open pancreatoduodenectomy for pancreatic or periampullary tumours (LEOPARD-2): a multicentre, patient-blinded, randomised controlled phase 2/3 trial. Lancet Gastroenterol Hepatol. 2019;4(3):199-207. doi:10.1016/S2468-1253(19)30004-4

456. Osmancik P, Herman D, Neuzil P, et al. Left Atrial Appendage Closure Versus Direct Oral Anticoagulants in High-Risk Patients With Atrial Fibrillation. J Am Coll Cardiol. 2020;75(25):3122-3135. doi:10.1016/j.jacc.2020.04.067

457. Makker V, Taylor MH, Aghajanian C, et al. Lenvatinib plus pembrolizumab in patients with advanced endometrial cancer. J Clin Oncol. 2020;38(26):2981-2992. doi:10.1200/JCO.19.02627

458. Makker V, Rasco D, Vogelzang NJ, et al. Lenvatinib plus pembrolizumab in patients with advanced endometrial cancer: an interim analysis of a multicentre, open-label, single-arm, phase 2 trial. Lancet Oncol. 2019;20(5):711-718. doi:10.1016/S1470-2045(19)30020-8

459. Herrlinger U, Tzaridis T, Mack F, et al. Lomustine-temozolomide combination therapy versus standard temozolomide therapy in patients with newly diagnosed glioblastoma with methylated MGMT promoter (CeTeG/NOA–09): a randomised, open-label, phase 3 trial. Lancet. 2019;393(10172):678-688. doi:10.1016/S0140-6736(18)31791-4

460. Burger JA, Barr PM, Robak T, et al. Long-term efficacy and safety of first-line ibrutinib treatment for patients with CLL/SLL: 5 years of follow-up from the phase 3 RESONATE-2 study. Leukemia. 2020;34(3):787-798. doi:10.1038/s41375-019-0602-x

461. Bradley JD, Hu C, Komaki RR, et al. Long-term results of NRG oncology RTOG 0617: Standard-versus high-dose chemoradiotherapy with or without cetuximab for unresectable stage III non-small-cell lung cancer. J Clin Oncol. 2020;38(7):706-714. doi:10.1200/JCO.19.01162

462. Yam JC, Jiang Y, Tang SM, et al. Low-Concentration Atropine for Myopia Progression (LAMP) Study: A Randomized, Double-Blinded, Placebo-Controlled Trial of 0.05%, 0.025%, and 0.01% Atropine Eye Drops in Myopia Control. Ophthalmology. 2019;126(1):113-124. doi:10.1016/j.ophtha.2018.05.029

463. Fenaux P, Platzbecker U, Mufti GJ, et al. Luspatercept in Patients with Lower-Risk Myelodysplastic Syndromes. N Engl J Med. 2020;382(2):140-151. doi:10.1056/nejmoa1908892

464. Nagel E, Greenwood JP, McCann GP, et al. Magnetic Resonance Perfusion or Fractional Flow Reserve in Coronary Disease. N Engl J Med. 2019;380(25):2418-2428. doi:10.1056/nejmoa1716734

465. Olivotto I, Oreziak A, Barriales-Villa R, et al. Mavacamten for treatment of symptomatic obstructive hypertrophic cardiomyopathy (EXPLORER-HCM): a randomised, double-blind, placebo-controlled, phase 3 trial. Lancet. 2020;396(10253):759-769. doi:10.1016/S0140-6736(20)31792-X

466. Ghosh TS, Rampelli S, Jeffery IB, et al. Mediterranean diet intervention alters the gut microbiome in older people reducing frailty and improving health status: The NU-AGE 1-year dietary intervention across five European countries. Gut. 2020;69(7):1218-1228. doi:10.1136/gutjnl-2019-319654

© 2023 Siena LM et al. *JAMA Network Open.*

467. Jeronimo CMP, Farias MEL, Val FFA, et al. Methylprednisolone as Adjunctive Therapy for Patients Hospitalized With Coronavirus Disease 2019 (COVID-19; Metcovid): A Randomized, Double-blind, Phase IIb, Placebo-controlled Trial. Clin Infect Dis. 2021;72(9):E373-E381. doi:10.1093/cid/ciaa1177

468. De Rooij T, Van Hilst J, Van Santvoort H, et al. Minimally Invasive Versus Open Distal Pancreatectomy (LEOPARD): A Multicenter Patient-blinded Randomized Controlled Trial. Ann Surg. 2019;269(1):2-9. doi:10.1097/SLA.0000000000002979

469. Stein EM, DiNardo CD, Fathi AT, et al. Molecular remission and response patterns in patients with mutant-IDH2 acute myeloid leukemia treated with enasidenib. Blood. 2019;133(7):676-687. doi:10.1182/blood-2018-08-869008

470. Johnston S, Martin M, Di Leo A, et al. MONARCH 3 final PFS: a randomized study of abemaciclib as initial therapy for advanced breast cancer. npj Breast Cancer. 2019;5(1). doi:10.1038/s41523-018-0097-z

471. Gong Q, Zhang P, Wang J, et al. Morbidity and mortality after lifestyle intervention for people with impaired glucose tolerance: 30-year results of the Da Qing Diabetes Prevention Outcome Study. Lancet Diabetes Endocrinol. 2019;7(6):452-461. doi:10.1016/S2213-8587(19)30093-2

472. Pasi KJ, Rangarajan S, Mitchell N, et al. Multiyear Follow-up of AAV5-hFVIII-SQ Gene Therapy for Hemophilia A. N Engl J Med. 2020;382(1):29-40. doi:10.1056/nejmoa1908490

473. Radonovich LJ, Simberkoff MS, Bessesen MT, et al. N95 respirators vs medical masks for preventing influenza among health care personnel: A randomized clinical trial. JAMA - J Am Med Assoc. 2019;322(9):824-833. doi:10.1001/jama.2019.11645

474. Hollon TC, Pandian B, Adapa AR, et al. Near real-time intraoperative brain tumor diagnosis using stimulated Raman histology and deep neural networks. Nat Med. 2020;26(1):52-58. doi:10.1038/s41591-019-0715-9

475. Shu CA, Gainor JF, Awad MM, et al. Neoadjuvant atezolizumab and chemotherapy in patients with resectable non-small-cell lung cancer: an open-label, multicentre, single-arm, phase 2 trial. Lancet Oncol. 2020;21(6):786-795. doi:10.1016/S1470-2045(20)30140-6

476. Provencio M, Nadal E, Insa A, et al. Neoadjuvant chemotherapy and nivolumab in resectable non-small-cell lung cancer (NADIM): an open-label, multicentre, single-arm, phase 2 trial. Lancet Oncol. 2020;21(11):1413-1422. doi:10.1016/S1470-2045(20)30453-8

477. Conroy T, Bosset JF, Etienne PL, et al. Neoadjuvant chemotherapy with FOLFIRINOX and preoperative chemoradiotherapy for patients with locally advanced rectal cancer (UNICANCER-PRODIGE 23): a multicentre, randomised, open-label, phase 3 trial. Lancet Oncol. 2021;22(5):702-715. doi:10.1016/S1470-2045(21)00079-6

478. Saura C, Oliveira M, Feng YH, et al. Neratinib plus capecitabine versus lapatinib plus capecitabine in HER2-positive metastatic breast cancer previously treated with ≥ 2 HER2-directed regimens: Phase III NALA trial. J Clin Oncol. 2020;38(27):3138-3149. doi:10.1200/JCO.20.00147

479. McCann ME, Berde C, Soriano S, et al. Neurodevelopmental outcome at 5 years of age after general anaesthesia or awake-regional anaesthesia in infancy (GAS): an international, multicentre, randomised, controlled equivalence trial. Lancet. 2019;393(10172):664-677. doi:10.1016/S0140-6736(18)32485-1

480. Wells AU, Flaherty KR, Brown KK, et al. Nintedanib in patients with progressive fibrosing interstitial lung diseases—subgroup analyses by interstitial lung disease diagnosis in the INBUILD trial: a randomised, double-blind, placebo-controlled, parallel-group trial. Lancet Respir Med. 2020;8(5):453-460. doi:10.1016/S2213-2600(20)30036-9

© 2023 Siena LM et al. *JAMA Network Open.*

481. Moore KN, Secord AA, Geller MA, et al. Niraparib monotherapy for late-line treatment of ovarian cancer (QUADRA): a multicentre, open-label, single-arm, phase 2 trial. Lancet Oncol. 2019;20(5):636-648. doi:10.1016/S1470-2045(19)30029-4

482. Scherpereel A, Mazieres J, Greillier L, et al. Nivolumab or nivolumab plus ipilimumab in patients with relapsed malignant pleural mesothelioma (IFCT-1501 MAPS2): a multicentre, open-label, randomised, non-comparative, phase 2 trial. Lancet Oncol. 2019;20(2):239-253. doi:10.1016/S1470-2045(18)30765-4

483. De Vivo DC, Bertini E, Swoboda KJ, et al. Nusinersen initiated in infants during the presymptomatic stage of spinal muscular atrophy: Interim efficacy and safety results from the Phase 2 NURTURE study. Neuromuscul Disord. 2019;29(11):842-856. doi:10.1016/j.nmd.2019.09.007

484. Mateo J, Porta N, Bianchini D, et al. Olaparib in patients with metastatic castration-resistant prostate cancer with DNA repair gene aberrations (TOPARP-B): a multicentre, open-label, randomised, phase 2 trial. Lancet Oncol. 2020;21(1):162-174. doi:10.1016/S1470-2045(19)30684-9

485. Robson ME, Tung N, Conte P, et al. OlympiAD final overall survival and tolerability results: Olaparib versus chemotherapy treatment of physician's choice in patients with a germline BRCA mutation and HER2-negative metastatic breast cancer. Ann Oncol. 2019;30(4):558-566. doi:10.1093/annonc/mdz012

486. Hammond J, Leister-Tebbe H, Gardner A, et al. Oral Nirmatrelvir for High-Risk, Nonhospitalized Adults with Covid-19. N Engl J Med. 2022;386(15):1397-1408. doi:10.1056/nejmoa2118542

487. Pratley R, Amod A, Hoff ST, et al. Oral semaglutide versus subcutaneous liraglutide and placebo in type 2 diabetes (PIONEER 4): a randomised, double-blind, phase 3a trial. Lancet. 2019;394(10192):39-50. doi:10.1016/S0140-6736(19)31271-1

488. Li HK, Rombach I, Zambellas R, et al. Oral versus Intravenous Antibiotics for Bone and Joint Infection. N Engl J Med. 2019;380(5):425-436. doi:10.1056/nejmoa1710926

489. Sequist L V., Han JY, Ahn MJ, et al. Osimertinib plus savolitinib in patients with EGFR mutation-positive, MET-amplified, non-small-cell lung cancer after progression on EGFR tyrosine kinase inhibitors: interim results from a multicentre, open-label, phase 1b study. Lancet Oncol. 2020;21(3):373-386. doi:10.1016/S1470-2045(19)30785-5

490. Goldberg SB, Schalper KA, Gettinger SN, et al. Pembrolizumab for management of patients with NSCLC and brain metastases: long-term results and biomarker analysis from a non-randomised, open-label, phase 2 trial. Lancet Oncol. 2020;21(5):655-663. doi:10.1016/S1470-2045(20)30111-X

491. Antonarakis ES, Piulats JM, Gross-Goupil M, et al. Pembrolizumab for treatment-refractory metastatic castration-resistant prostate cancer: Multicohort, open-label phase II KEYNOTE-199 study. J Clin Oncol. 2020;38(5):395-405. doi:10.1200/JCO.19.01638

492. Rudin CM, Awad MM, Navarro A, et al. Pembrolizumab or placebo plus etoposide and platinum as first-line therapy for extensive-stage small-cell lung cancer: Randomized, double-blind, phase III KEYNOTE-604 Study. J Clin Oncol. 2020;38(21):2369-2379. doi:10.1200/JCO.20.00793

493. Powles T, Plimack ER, Soulières D, et al. Pembrolizumab plus axitinib versus sunitinib monotherapy as first-line treatment of advanced renal cell carcinoma (KEYNOTE-426): extended follow-up from a randomised, open-label, phase 3 trial. Lancet Oncol. 2020;21(12):1563-1573. doi:10.1016/S1470-2045(20)30436-8

494. Sun JM, Shen L, Shah MA, et al. Pembrolizumab plus chemotherapy versus chemotherapy alone for first-line treatment of advanced oesophageal cancer (KEYNOTE-590): a randomised, placebo-controlled, phase 3 study. Lancet. 2021;398(10302):759-771. doi:10.1016/S0140-6736(21)01234-4

© 2023 Siena LM et al. *JAMA Network Open.*

495. Loi S, Giobbie-Hurder A, Gombos A, et al. Pembrolizumab plus trastuzumab in trastuzumab-resistant, advanced, HER2-positive breast cancer (PANACEA): a single-arm, multicentre, phase 1b–2 trial. Lancet Oncol. 2019;20(3):371-382. doi:10.1016/S1470-2045(18)30812-X

496. Holm NR, Mäkikallio T, Lindsay MM, et al. Percutaneous coronary angioplasty versus coronary artery bypass grafting in the treatment of unprotected left main stenosis: updated 5-year outcomes from the randomised, non-inferiority NOBLE trial. Lancet. 2020;395(10219):191-199. doi:10.1016/S0140-6736(19)32972-1

497. Thuijs DJFM, Kappetein AP, Serruys PW, et al. Percutaneous coronary intervention versus coronary artery bypass grafting in patients with three-vessel or left main coronary artery disease: 10-year follow-up of the multicentre randomised controlled SYNTAX trial. Lancet. 2019;394(10206):1325-1334. doi:10.1016/S0140-6736(19)31997-X

498. Meric-Bernstam F, Hurwitz H, Raghav KPS, et al. Pertuzumab plus trastuzumab for HER2-amplified metastatic colorectal cancer (MyPathway): an updated report from a multicentre, open-label, phase 2a, multiple basket study. Lancet Oncol. 2019;20(4):518-530. doi:10.1016/S1470-2045(18)30904-5

499. Swain SM, Miles D, Kim SB, et al. Pertuzumab, trastuzumab, and docetaxel for HER2-positive metastatic breast cancer (CLEOPATRA): end-of-study results from a double-blind, randomised, placebo-controlled, phase 3 study. Lancet Oncol. 2020;21(4):519-530. doi:10.1016/S1470-2045(19)30863-0

500. Hong DS, Kang YK, Borad M, et al. Phase 1 study of MRX34, a liposomal miR-34a mimic, in patients with advanced solid tumours. Br J Cancer. 2020;122(11):1630-1637. doi:10.1038/s41416-020-0802-1

501. Miller T, Cudkowicz M, Shaw PJ, et al. Phase 1–2 Trial of Antisense Oligonucleotide Tofersen for SOD1 ALS . N Engl J Med. 2020;383(2):109-119. doi:10.1056/nejmoa2003715

502. Falsey AR, Sobieszczyk ME, Hirsch I, et al. Phase 3 Safety and Efficacy of AZD1222 (ChAdOx1 nCoV-19) Covid-19 Vaccine. N Engl J Med. 2021;385(25):2348-2360. doi:10.1056/nejmoa2105290

503. Balwani M, Sardh E, Ventura P, et al. Phase 3 Trial of RNAi Therapeutic Givosiran for Acute Intermittent Porphyria. N Engl J Med. 2020;382(24):2289-2301. doi:10.1056/nejmoa1913147

504. Robinson CG, Samson PP, Moore KMS, et al. Phase I/II Trial of Electrophysiology-Guided Noninvasive Cardiac Radioablation for Ventricular Tachycardia. Circulation. 2019;139(3):313-321. doi:10.1161/CIRCULATIONAHA.118.038261

505. Sciascia S, Aprà F, Baffa A, et al. Pilot prospective open, single-arm multicentre study on off-label use of tocilizumab in patients with severe COVID-19. Clin Exp Rheumatol. 2020;38(3):529-532.

506. Maher TM, Corte TJ, Fischer A, et al. Pirfenidone in patients with unclassifiable progressive fibrosing interstitial lung disease: a double-blind, randomised, placebo-controlled, phase 2 trial. Lancet Respir Med. 2020;8(2):147-157. doi:10.1016/S2213-2600(19)30341-8

507. Rosenberg JE, O'Donnell PH, Balar A V., et al. Pivotal trial of enfortumab vedotin in urothelial carcinoma after platinum and anti-programmed death 1/programmed death ligand 1 therapy. J Clin Oncol. 2019;37(29):2592-2600. doi:10.1200/JCO.19.01140

508. Walsh M, Merkel PA, Peh CA, et al. Plasma Exchange and Glucocorticoids in Severe ANCA-Associated Vasculitis. N Engl J Med. 2020;382(7):622-631. doi:10.1056/nejmoa1803537

509. Munné S, Kaplan B, Frattarelli JL, et al. Preimplantation genetic testing for aneuploidy versus morphology as selection criteria for single frozen-thawed embryo transfer in good-prognosis patients: a

© 2023 Siena LM et al. *JAMA Network Open.*

multicenter randomized clinical trial. Fertil Steril. 2019;112(6):1071-1079.e7. doi:10.1016/j.fertnstert.2019.07.1346

510. Pastorino U, Silva M, Sestini S, et al. Prolonged lung cancer screening reduced 10-year mortality in the MILD trial: new confirmation of lung cancer screening efficacy. Ann Oncol. 2019;30(7):1162-1169. doi:10.1093/annonc/mdz117

511. Armstrong AJ, Halabi S, Luo J, et al. Prospective multicenter validation of androgen receptor splice variant 7 and hormone therapy resistance in high-risk castration-resistant prostate cancer: The PROPHECY study. J Clin Oncol. 2019;37(13):1120-1129. doi:10.1200/JCO.18.01731

512. Cortes JE, Khaled S, Martinelli G, et al. Quizartinib versus salvage chemotherapy in relapsed or refractory FLT3-ITD acute myeloid leukaemia (QuANTUM-R): a multicentre, randomised, controlled, open-label, phase 3 trial. Lancet Oncol. 2019;20(7):984-997. doi:10.1016/S1470-2045(19)30150-0

513. Nichols AC, Theurer J, Prisman E, et al. Radiotherapy versus transoral robotic surgery and neck dissection for oropharyngeal squamous cell carcinoma (ORATOR): an open-label, phase 2, randomised trial. Lancet Oncol. 2019;20(10):1349-1359. doi:10.1016/S1470-2045(19)30410-3

514. Kudo M, Ueshima K, Ikeda M, et al. Randomised, multicentre prospective trial of transarterial chemoembolisation (TACE) plus sorafenib as compared with TACE alone in patients with hepatocellular carcinoma: TACTICS trial. Gut. 2020;69(8):1492-1501. doi:10.1136/gutjnl-2019-318934

515. Cortes JE, Heidel FH, Hellmann A, et al. Randomized comparison of low dose cytarabine with or without glasdegib in patients with newly diagnosed acute myeloid leukemia or high-risk myelodysplastic syndrome. Leukemia. 2019;33(2):379-389. doi:10.1038/s41375-018-0312-9

516. Fokas E, Allgäuer M, Polat B, et al. Randomized phase II trial of chemoradiotherapy plus induction or consolidation chemotherapy as total neoadjuvant therapy for locally advanced rectal cancer: CAO/ArO/AIO-12. J Clin Oncol. 2019;37(34):3212-3222. doi:10.1200/JCO.19.00308

517. Fradet Y, Bellmunt J, Vaughn DJ, et al. Randomized phase III KEYNOTE-045 trial of pembrolizumab versus paclitaxel, docetaxel, or vinflunine in recurrent advanced urothelial cancer: Results of >2 years of follow-up. Ann Oncol. 2019;30(6):970-976. doi:10.1093/annonc/mdz127

518. Younes A, Sehn LH, Johnson P, et al. Randomized phase III trial of ibrutinib and rituximab plus cyclophosphamide, doxorubicin, vincristine, and prednisone in non–germinal center B-cell diffuse large B-cell lymphoma. J Clin Oncol. 2019;37(15):1285-1295. doi:10.1200/JCO.18.02403

519. Caraballo R. Randomized trial of three anticonvulsant medications for status epilepticus. Arch Argent Pediatr. 2020;118:E211-E216. doi:10.1056/nejmoa1905795

520. Egan MF, Kost J, Voss T, et al. Randomized Trial of Verubecestat for Prodromal Alzheimer's Disease. N Engl J Med. 2019;380(15):1408-1420. doi:10.1056/nejmoa1812840

521. Palhano-Fontes F, Barreto D, Onias H, et al. Rapid antidepressant effects of the psychedelic ayahuasca in treatment-resistant depression: A randomized placebo-controlled trial. Psychol Med. 2019;49(4):655-663. doi:10.1017/S0033291718001356

522. Turakhia MP, Desai M, Hedlin H, et al. Rationale and design of a large-scale, app-based study to identify cardiac arrhythmias using a smartwatch: The Apple Heart Study. Am Heart J. 2019;207:66-75. doi:10.1016/j.ahj.2018.09.002

523. Weinreich DM, Sivapalasingam S, Norton T, et al. REGEN-COV Antibody Combination and Outcomes in Outpatients with Covid-19. N Engl J Med. 2021;385(23):e81. doi:10.1056/nejmoa2108163

© 2023 Siena LM et al. *JAMA Network Open.*

524. Fukuoka S, Hara H, Takahashi N, et al. Regorafenib plus Nivolumab in Patients with Advanced Gastric or Colorectal Cancer: An Open-Label, Dose-Escalation, and Dose-Expansion Phase Ib Trial (REGONIVO, EPOC1603). J Clin Oncol. 2020;38(18):2053-2061. doi:10.1200/JCO.19.03296

525. Harrison SA, Bashir MR, Guy CD, et al. Resmetirom (MGL-3196) for the treatment of non-alcoholic steatohepatitis: a multicentre, randomised, double-blind, placebo-controlled, phase 2 trial. Lancet. 2019;394(10213):2012-2024. doi:10.1016/S0140-6736(19)32517-6

526. Liu K, Zhang W, Yang Y, Zhang J, Li Y, Chen Y. Respiratory rehabilitation in elderly patients with COVID-19: A randomized controlled study. Complement Ther Clin Pract. 2020;39. doi:10.1016/j.ctcp.2020.101166

527. Bonaca MP, Bauersachs RM, Anand SS, et al. Rivaroxaban in Peripheral Artery Disease after Revascularization. N Engl J Med. 2020;382(21):1994-2004. doi:10.1056/nejmoa2000052

528. Chen N, Hao C, Peng X, et al. Roxadustat for Anemia in Patients with Kidney Disease Not Receiving Dialysis. N Engl J Med. 2019;381(11):1001-1010. doi:10.1056/nejmoa1813599

529. Chen N, Hao C, Liu BC, et al. Roxadustat Treatment for Anemia in Patients Undergoing Long-Term Dialysis. N Engl J Med. 2019;381(11):1011-1022. doi:10.1056/nejmoa1901713

530. Abida W, Patnaik A, Campbell D, et al. Rucaparib in Men with Metastatic Castration-Resistant Prostate Cancer Harboring a BRCA1 or BRCA2 Gene Alteration. J Clin Oncol. 2020;38(32):3763-3772. doi:10.1200/JCO.20.01035

531. Zeiser R, von Bubnoff N, Butler J, et al. Ruxolitinib for Glucocorticoid-Refractory Acute Graft-versus-Host Disease. N Engl J Med. 2020;382(19):1800-1810. doi:10.1056/nejmoa1917635

532. Cao Y, Wei J, Zou L, et al. Ruxolitinib in treatment of severe coronavirus disease 2019 (COVID-19): A multicenter, single-blind, randomized controlled trial. J Allergy Clin Immunol. 2020;146(1):137-146.e3. doi:10.1016/j.jaci.2020.05.019

533. Bardia A, Hurvitz SA, Tolaney SM, et al. Sacituzumab Govitecan in Metastatic Triple-Negative Breast Cancer. N Engl J Med. 2021;384(16):1529-1541. doi:10.1056/nejmoa2028485

534. Monk PD, Marsden RJ, Tear VJ, et al. Safety and efficacy of inhaled nebulised interferon beta-1a (SNG001) for treatment of SARS-CoV-2 infection: a randomised, double-blind, placebo-controlled, phase 2 trial. Lancet Respir Med. 2021;9(2):196-206. doi:10.1016/S2213-2600(20)30511-7

535. Ella R, Vadrevu KM, Jogdand H, et al. Safety and immunogenicity of an inactivated SARS-CoV-2 vaccine, BBV152: a double-blind, randomised, phase 1 trial. Lancet Infect Dis. 2021;21(5):637-646. doi:10.1016/S1473-3099(20)30942-7

536. Liu X, Shaw RH, Stuart ASV, et al. Safety and immunogenicity of heterologous versus homologous prime-boost schedules with an adenoviral vectored and mRNA COVID-19 vaccine (Com-COV): a single-blind, randomised, non-inferiority trial. Lancet. 2021;398(10303):856-869. doi:10.1016/S0140-6736(21)01694-9

537. Munro APS, Janani L, Cornelius V, et al. Safety and immunogenicity of seven COVID-19 vaccines as a third dose (booster) following two doses of ChAdOx1 nCov-19 or BNT162b2 in the UK (COV-BOOST): a blinded, multicentre, randomised, controlled, phase 2 trial. Lancet. 2021;398(10318):2258-2276. doi:10.1016/S0140-6736(21)02717-3

538. Moayyedi P, Eikelboom JW, Bosch J, et al. Safety of Proton Pump Inhibitors Based on a Large, Multi-Year, Randomized Trial of Patients Receiving Rivaroxaban or Aspirin. Gastroenterology. 2019;157(3):682-691.e2. doi:10.1053/j.gastro.2019.05.056

© 2023 Siena LM et al. *JAMA Network Open.*

539. Wang F, Wei XL, Wang FH, et al. Safety, efficacy and tumor mutational burden as a biomarker of overall survival benefit in chemo-refractory gastric cancer treated with toripalimab, a PD-1 antibody in phase Ib/II clinical trial NCT02915432. Ann Oncol. 2019;30(9):1479-1486. doi:10.1093/annonc/mdz197

540. Wu Z, Hu Y, Xu M, et al. Safety, tolerability, and immunogenicity of an inactivated SARS-CoV-2 vaccine (CoronaVac) in healthy adults aged 60 years and older: a randomised, double-blind, placebo-controlled, phase 1/2 clinical trial. Lancet Infect Dis. 2021;21(6):803-812. doi:10.1016/S1473-3099(20)30987-7

541. Gazzard G, Konstantakopoulou E, Garway-Heath D, et al. Selective laser trabeculoplasty versus eye drops for first-line treatment of ocular hypertension and glaucoma (LiGHT): a multicentre randomised controlled trial. Lancet. 2019;393(10180):1505-1516. doi:10.1016/S0140-6736(18)32213-X

542. Fangusaro J, Onar-Thomas A, Young Poussaint T, et al. Selumetinib in paediatric patients with BRAF-aberrant or neurofibromatosis type 1-associated recurrent, refractory, or progressive low-grade glioma: a multicentre, phase 2 trial. Lancet Oncol. 2019;20(7):1011-1022. doi:10.1016/S1470-2045(19)30277-3

543. Lee HJ, Hyung WJ, Yang HK, et al. Short-term outcomes of a multicenter randomized controlled trial comparing laparoscopic distal gastrectomy with D2 lymphadenectomy to open distal gastrectomy for locally advanced gastric cancer (KLASS-02-RCT). Ann Surg. 2019;270(6):983-991. doi:10.1097/SLA.0000000000003217

544. Konstantinopoulos PA, Waggoner S, Vidal GA, et al. Single-Arm Phases 1 and 2 Trial of Niraparib in Combination with Pembrolizumab in Patients with Recurrent Platinum-Resistant Ovarian Carcinoma. JAMA Oncol. 2019;5(8):1141-1149. doi:10.1001/jamaoncol.2019.1048

545. Paramsothy S, Nielsen S, Kamm MA, et al. Specific Bacteria and Metabolites Associated With Response to Fecal Microbiota Transplantation in Patients With Ulcerative Colitis. Gastroenterology. 2019;156(5):1440-1454.e2. doi:10.1053/j.gastro.2018.12.001

546. Ball D, Mai GT, Vinod S, et al. Stereotactic ablative radiotherapy versus standard radiotherapy in stage 1 non-small-cell lung cancer (TROG 09.02 CHISEL): a phase 3, open-label, randomised controlled trial. Lancet Oncol. 2019;20(4):494-503. doi:10.1016/S1470-2045(18)30896-9

547. O'Brien MP, Forleo-Neto E, Musser BJ, et al. Subcutaneous REGEN-COV Antibody Combination to Prevent Covid-19. N Engl J Med. 2021;385(13):1184-1195. doi:10.1056/nejmoa2109682

548. Bakker MF, de Lange S V., Pijnappel RM, et al. Supplemental MRI Screening for Women with Extremely Dense Breast Tissue. N Engl J Med. 2019;381(22):2091-2102. doi:10.1056/nejmoa1903986

549. Smeland S, Bielack SS, Whelan J, et al. Survival and prognosis with osteosarcoma: outcomes in more than 2000 patients in the EURAMOS-1 (European and American Osteosarcoma Study) cohort. Eur J Cancer. 2019;109:36-50. doi:10.1016/j.ejca.2018.11.027

550. Ascierto PA, Long G V., Robert C, et al. Survival Outcomes in Patients with Previously Untreated BRAF Wild-Type Advanced Melanoma Treated with Nivolumab Therapy: Three-Year Follow-up of a Randomized Phase 3 Trial. JAMA Oncol. 2019;5(2):187-194. doi:10.1001/jamaoncol.2018.4514

551. Hussain M, Mateo J, Fizazi K, et al. Survival with Olaparib in Metastatic Castration-Resistant Prostate Cancer. N Engl J Med. 2020;383(24):2345-2357. doi:10.1056/nejmoa2022485

552. Salles G, Duell J, González Barca E, et al. Tafasitamab plus lenalidomide in relapsed or refractory diffuse large B-cell lymphoma (L-MIND): a multicentre, prospective, single-arm, phase 2 study. Lancet Oncol. 2020;21(7):978-988. doi:10.1016/S1470-2045(20)30225-4

© 2023 Siena LM et al. *JAMA Network Open.*

553. Pemmaraju N, Lane AA, Sweet KL, et al. Tagraxofusp in Blastic Plasmacytoid Dendritic-Cell Neoplasm. N Engl J Med. 2019;380(17):1628-1637. doi:10.1056/nejmoa1815105

554. Lascarrou JB, Merdji H, Le Gouge A, et al. Targeted Temperature Management for Cardiac Arrest with Nonshockable Rhythm. N Engl J Med. 2019;381(24):2327-2337. doi:10.1056/nejmoa1906661

555. Wilkinson MJ, Manoogian ENC, Zadourian A, et al. Ten-Hour Time-Restricted Eating Reduces Weight, Blood Pressure, and Atherogenic Lipids in Patients with Metabolic Syndrome. Cell Metab. 2020;31(1):92-104.e5. doi:10.1016/j.cmet.2019.11.004

556. Douglas RS, Kahaly GJ, Patel A, et al. Teprotumumab for the Treatment of Active Thyroid Eye Disease. N Engl J Med. 2020;382(4):341-352. doi:10.1056/nejmoa1910434

557. Lopes RD, de Barros e Silva PGM, Furtado RHM, et al. Therapeutic versus prophylactic anticoagulation for patients admitted to hospital with COVID-19 and elevated D-dimer concentration (ACTION): an open-label, multicentre, randomised, controlled trial. Lancet. 2021;397(10291):2253-2263. doi:10.1016/S0140-6736(21)01203-4

558. Ready N, Farago AF, de Braud F, et al. Third-Line Nivolumab Monotherapy in Recurrent SCLC: CheckMate 032. J Thorac Oncol. 2019;14(2):237-244. doi:10.1016/j.jtho.2018.10.003

559. Gray JE, Villegas A, Daniel D, et al. Three-Year Overall Survival with Durvalumab after Chemoradiotherapy in Stage III NSCLC—Update from PACIFIC. J Thorac Oncol. 2020;15(2):288-293. doi:10.1016/j.jtho.2019.10.002

560. Frías JP, Davies MJ, Rosenstock J, et al. Tirzepatide versus Semaglutide Once Weekly in Patients with Type 2 Diabetes. N Engl J Med. 2021;385(6):503-515. doi:10.1056/nejmoa2107519

561. Guimarães PO, Quirk D, Furtado RH, et al. Tofacitinib in Patients Hospitalized with Covid-19 Pneumonia. N Engl J Med. 2021;385(5):406-415. doi:10.1056/nejmoa2101643

562. Murphy JE, Wo JY, Ryan DP, et al. Total Neoadjuvant Therapy with FOLFIRINOX in Combination with Losartan Followed by Chemoradiotherapy for Locally Advanced Pancreatic Cancer: A Phase 2 Clinical Trial. JAMA Oncol. 2019;5(7):1020-1027. doi:10.1001/jamaoncol.2019.0892

563. Nickenig G, Weber M, Lurz P, et al. Transcatheter edge-to-edge repair for reduction of tricuspid regurgitation: 6-month outcomes of the TRILUMINATE single-arm study. Lancet. 2019;394(10213):2002-2011. doi:10.1016/S0140-6736(19)32600-5

564. Wollenberg A, Howell MD, Guttman-Yassky E, et al. Treatment of atopic dermatitis with tralokinumab, an anti–IL-13 mAb. J Allergy Clin Immunol. 2019;143(1):135-141. doi:10.1016/j.jaci.2018.05.029

565. Conradie F, Diacon AH, Ngubane N, et al. Treatment of Highly Drug-Resistant Pulmonary Tuberculosis. N Engl J Med. 2020;382(10):893-902. doi:10.1056/nejmoa1901814

566. Carhart-Harris R, Giribaldi B, Watts R, et al. Trial of Psilocybin versus Escitalopram for Depression. N Engl J Med. 2021;384(15):1402-1411. doi:10.1056/nejmoa2032994

567. Yamamura T, Kleiter I, Fujihara K, et al. Trial of Satralizumab in Neuromyelitis Optica Spectrum Disorder. N Engl J Med. 2019;381(22):2114-2124. doi:10.1056/nejmoa1901747

568. Furie R, Rovin BH, Houssiau F, et al. Two-Year, Randomized, Controlled Trial of Belimumab in Lupus Nephritis. N Engl J Med. 2020;383(12):1117-1128. doi:10.1056/nejmoa2001180

569. Camidge DR, Dziadziuszko R, Peters S, et al. Updated Efficacy and Safety Data and Impact of the EML4-ALK Fusion Variant on the Efficacy of Alectinib in Untreated ALK-Positive Advanced Non–Small Cell Lung

© 2023 Siena LM et al. *JAMA Network Open.*

Cancer in the Global Phase III ALEX Study. J Thorac Oncol. 2019;14(7):1233-1243. doi:10.1016/j.jtho.2019.03.007

570. Choueiri TK, Motzer RJ, Rini BI, et al. Updated efficacy results from the JAVELIN Renal 101 trial: first-line avelumab plus axitinib versus sunitinib in patients with advanced renal cell carcinoma. Ann Oncol. 2020;31(8):1030-1039. doi:10.1016/j.annonc.2020.04.010

571. Sands BE, Peyrin-Biroulet L, Loftus E V., et al. Vedolizumab versus Adalimumab for Moderate-to-Severe Ulcerative Colitis. N Engl J Med. 2019;381(13):1215-1226. doi:10.1056/nejmoa1905725

572. Wei AH, Montesinos P, Ivanov V, et al. Venetoclax plus LDAC for newly diagnosed AML ineligible for intensive chemotherapy: a phase 3 randomized placebo-controlled trial. Blood. 2020;135(24):2137-2145. doi:10.1182/BLOOD.2020004856

573. Pittas AG, Dawson-Hughes B, Sheehan P, et al. Vitamin D Supplementation and Prevention of Type 2 Diabetes. N Engl J Med. 2019;381(6):520-530. doi:10.1056/nejmoa1900906

574. Witztum JL, Gaudet D, Freedman SD, et al. Volanesorsen and Triglyceride Levels in Familial Chylomicronemia Syndrome. N Engl J Med. 2019;381(6):531-542. doi:10.1056/nejmoa1715944

575. Munch Roager H, Vogt JK, Kristensen M, et al. Whole grain-rich diet reduces body weight and systemic low-grade inflammation without inducing major changes of the gut microbiome: A randomised cross-over trial. Gut. 2019;68(1):83-93. doi:10.1136/gutjnl-2017-314786

576. Halliday BP, Wassall R, Lota AS, et al. Withdrawal of pharmacological treatment for heart failure in patients with recovered dilated cardiomyopathy (TRED-HF): an open-label, pilot, randomised trial. Lancet. 2019;393(10166):61-73. doi:10.1016/S0140-6736(18)32484-X

577. Villanueva C, Albillos A, Genescà J, et al. β blockers to prevent decompensation of cirrhosis in patients with clinically significant portal hypertension (PREDESCI): a randomised, double-blind, placebo-controlled, multicentre trial. Lancet. 2019;393(10181):1597-1608. doi:10.1016/S0140-6736(18)31875-0

578. Mateos MV, Cavo M, Blade J, et al. Overall survival with daratumumab, bortezomib, melphalan, and prednisone in newly diagnosed multiple myeloma (ALCYONE): a randomised, open-label, phase 3 trial. Lancet. 2020;395(10218):132-141. doi:10.1016/S0140-6736(19)32956-3

579. Mok T, Camidge DR, Gadgeel SM, et al. Updated overall survival and final progression-free survival data for patients with treatment-naive advanced ALK-positive non-small-cell lung cancer in the ALEX study. Ann Oncol. 2020;31(8):1056-1064. doi:10.1016/j.annonc.2020.04.478

580. Rabinowich L, Grupper A, Baruch R, et al. Low immunogenicity to SARS-CoV-2 vaccination among liver transplant recipients. J Hepatol. 2021;75(2):435-438. doi:10.1016/j.jhep.2021.04.020

581. Bezjak A, Paulus R, Gaspar LE, et al. Safety and efficacy of a five-fraction stereotactic body radiotherapy schedule for centrally located non–small-cell lung cancer: NRG Oncology/RTOG 0813 trial. J Clin Oncol. 2019;37(15):1316-1325. doi:10.1200/JCO.18.00622

582. Hammerich L, Marron TU, Upadhyay R, et al. Systemic clinical tumor regressions and potentiation of PD1 blockade with in situ vaccination. Nat Med. 2019;25(5):814-824. doi:10.1038/s41591-019-0410-x

583. Vicini FA, Cecchini RS, White JR, et al. Long-term primary results of accelerated partial breast irradiation after breast-conserving surgery for early-stage breast cancer: a randomised, phase 3, equivalence trial. Lancet. 2019;394(10215):2155-2164. doi:10.1016/S0140-6736(19)32514-0

© 2023 Siena LM et al. *JAMA Network Open.*

584. Leon-Castillo A, De Boer SM, Powell ME, et al. Molecular classification of the PORTEC-3 trial for high-risk endometrial cancer: Impact on prognosis and benefit from adjuvant therapy. J Clin Oncol. 2020;38(29):3388-3397. doi:10.1200/JCO.20.00549

585. Timing of Initiation of Renal-Replacement Therapy in Acute Kidney Injury. N Engl J Med. 2020;383(3):240-251. doi:10.1056/nejmoa2000741

586. Gross AM, Wolters PL, Dombi E, et al. Selumetinib in Children with Inoperable Plexiform Neurofibromas. N Engl J Med. 2020;382(15):1430-1442. doi:10.1056/nejmoa1912735

587. Johnston SC, Amarenco P, Denison H, et al. Ticagrelor and Aspirin or Aspirin Alone in Acute Ischemic Stroke or TIA. N Engl J Med. 2020;383(3):207-217. doi:10.1056/nejmoa1916870

588. Fleischmann R, Pangan AL, Song IH, et al. Upadacitinib Versus Placebo or Adalimumab in Patients With Rheumatoid Arthritis and an Inadequate Response to Methotrexate: Results of a Phase III, Double-Blind, Randomized Controlled Trial. Arthritis Rheumatol. 2019;71(11):1788-1800. doi:10.1002/art.41032

589. Lamarca A, Palmer DH, Wasan HS, et al. Second-line FOLFOX chemotherapy versus active symptom control for advanced biliary tract cancer (ABC-06): a phase 3, open-label, randomised, controlled trial. Lancet Oncol. 2021;22(5):690-701. doi:10.1016/S1470-2045(21)00027-9

590. Davoudi-Monfared E, Rahmani H, Khalili H, et al. A randomized clinical trial of the efficacy and safety of interferon β-1a in treatment of severe COVID-19. Antimicrob Agents Chemother. 2020;64(9). doi:10.1128/AAC.01061-20

591. Migden MR, Khushalani NI, Chang ALS, et al. Cemiplimab in locally advanced cutaneous squamous cell carcinoma: results from an open-label, phase 2, single-arm trial. Lancet Oncol. 2020;21(2):294-305. doi:10.1016/S1470-2045(19)30728-4

592. Andreux PA, Blanco-Bose W, Ryu D, et al. The mitophagy activator urolithin A is safe and induces a molecular signature of improved mitochondrial and cellular health in humans. Nat Metab. 2019;1(6):595-603. doi:10.1038/s42255-019-0073-4

593. Wu YL, Lu S, Cheng Y, et al. Nivolumab Versus Docetaxel in a Predominantly Chinese Patient Population With Previously Treated Advanced NSCLC: CheckMate 078 Randomized Phase III Clinical Trial. J Thorac Oncol. 2019;14(5):867-875. doi:10.1016/j.jtho.2019.01.006

594. Hirano I, Dellon ES, Hamilton JD, et al. Efficacy of Dupilumab in a Phase 2 Randomized Trial of Adults With Active Eosinophilic Esophagitis. Gastroenterology. 2020;158(1):111-122.e10. doi:10.1053/j.gastro.2019.09.042

595. Lu Y, Xue J, Deng T, et al. Safety and feasibility of CRISPR-edited T cells in patients with refractory non-small-cell lung cancer. Nat Med. 2020;26(5):732-740. doi:10.1038/s41591-020-0840-5

596. Bangalore S, Maron DJ, O'Brien SM, et al. Management of Coronary Disease in Patients with Advanced Kidney Disease. N Engl J Med. 2020;382(17):1608-1618. doi:10.1056/nejmoa1915925

597. Kim RD, Chung V, Alese OB, et al. A Phase 2 Multi-institutional Study of Nivolumab for Patients with Advanced Refractory Biliary Tract Cancer. JAMA Oncol. 2020;6(6):888-894. doi:10.1001/jamaoncol.2020.0930

598. Tawbi HA, Schadendorf D, Lipson EJ, et al. Relatlimab and Nivolumab versus Nivolumab in Untreated Advanced Melanoma. N Engl J Med. 2022;386(1):24-34. doi:10.1056/nejmoa2109970

© 2023 Siena LM et al. *JAMA Network Open.*

599. Kater AP, Seymour JF, Hillmen P, et al. Fixed duration of venetoclax-rituximab in relapsed/refractory chronic lymphocytic leukemia eradicates minimal residual disease and prolongs survival: Post-treatment follow-up of the Murano phase III study. J Clin Oncol. 2019;37(4):269-277. doi:10.1200/JCO.18.01580

600. Hauser SL, Bar-Or A, Cohen JA, et al. Ofatumumab versus Teriflunomide in Multiple Sclerosis. N Engl J Med. 2020;383(6):546-557. doi:10.1056/nejmoa1917246

© 2023 Siena LM et al. *JAMA Network Open.*
