# Supplementary material for: Industry Involvement and Transparency in the Most Cited Clinical Trials, 2019-2022
Source: JAMA Netw Open. 2023 Nov 14;6(11):e2343425. doi: 10.1001/jamanetworkopen.2023.43425 (PMC10646728; doi:10.1001/jamanetworkopen.2023.43425)
Supplement: Supplement 2. — Data Sharing Statement [file jamanetwopen-e2343425-s002.pdf]

Data Sharing Statement

Siena. Industry Involvement and Transparency in the Most Cited Clinical Trials, 2019-2022. *JAMA Netw Open*. Published November 16, 2023. doi:10.1001/jamanetworkopen.2023.43425

Data
Data available: Yes
Data types: Data (not involving human participants)
How to access data: Data will be made available by the authors (jioannid@stanford.edu) upon request
When available: With publication

Supporting Documents
Document types: None

Additional Information
Who can access the data: Researchers whose proposed use of the data has been approved
Types of analyses: Any purpose
Mechanisms of data availability: After approval
